# Genome-Wide Characterization of Transcriptional Patterns in High and Low Antibody Responders to Rubella Vaccination

**DOI:** 10.1371/journal.pone.0062149

**Published:** 2013-05-01

**Authors:** Iana H. Haralambieva, Ann L. Oberg, Inna G. Ovsyannikova, Richard B. Kennedy, Diane E. Grill, Sumit Middha, Brian M. Bot, Vivian W. Wang, David I. Smith, Robert M. Jacobson, Gregory A. Poland

**Affiliations:** 1 Vaccine Research Group, Mayo Clinic, Rochester, Minnesota, United States of America; 2 Program in Translational Immunovirology and Biodefense, Mayo Clinic, Rochester, Minnesota, United States of America; 3 Department of Health Sciences Research, Division of Biomedical Statistics and Informatics, Mayo Clinic, Rochester, Minnesota, United States of America; 4 Department of Pediatric and Adolescent Medicine, Mayo Clinic, Rochester, Minnesota, United States of America; 5 Sage Bionetworks, Seattle, Washington, United States of America; 6 Department of Laboratory Medicine and Pathology, Mayo Clinic, Rochester, Minnesota, United States of America; Federal University of São Paulo, Brazil

## Abstract

Immune responses to current rubella vaccines demonstrate significant inter-individual variability. We performed mRNA-Seq profiling on PBMCs from high and low antibody responders to rubella vaccination to delineate transcriptional differences upon viral stimulation. Generalized linear models were used to assess the per gene fold change (FC) for stimulated versus unstimulated samples or the interaction between outcome and stimulation. Model results were evaluated by both FC and p-value. Pathway analysis and self-contained gene set tests were performed for assessment of gene group effects.

Of 17,566 detected genes, we identified 1,080 highly significant differentially expressed genes upon viral stimulation (p<1.00E^−15^, FDR<1.00E^−14^), including various immune function and inflammation-related genes, genes involved in cell signaling, cell regulation and transcription, and genes with unknown function. Analysis by immune outcome and stimulation status identified 27 genes (p≤0.0006 and FDR≤0.30) that responded differently to viral stimulation in high vs. low antibody responders, including major histocompatibility complex (MHC) class I genes (*HLA-A*, *HLA-B* and *B2M* with p = 0.0001, p = 0.0005 and p = 0.0002, respectively), and two genes related to innate immunity and inflammation (*EMR3* and *MEFV* with p = 1.46E^−08^ and p = 0.0004, respectively). Pathway and gene set analysis also revealed transcriptional differences in antigen presentation and innate/inflammatory gene sets and pathways between high and low responders. Using mRNA-Seq genome-wide transcriptional profiling, we identified antigen presentation and innate/inflammatory genes that may assist in explaining rubella vaccine-induced immune response variations. Such information may provide new scientific insights into vaccine-induced immunity useful in rational vaccine development and immune response monitoring.

## Introduction

We and others have demonstrated the potential of next-generation sequencing (NGS) technology to provide a more detailed multidimensional view of host-pathogen interactions and immune response, and for adding new insights into infection pathogenesis, immunity and vaccine development [1], [2].

The influence of host genetic determinants on susceptibility to infections and inter-individual variability in vaccine-induced immune responses has been previously recognized [3]–[5]. Given the finding of high heritability (45.7%) of immune responses to rubella vaccine [6], we demonstrated that HLA polymorphisms (including haplotypes and supertypes), cytokine and cytokine receptor, Toll-like receptor, vitamin A and D receptors, antiviral effector, and other innate immunity gene polymorphisms significantly influence immune responses following live rubella viral immunization, but do not fully account for all the observed immune response variability [7]–[18]. Thus, our findings and the literature support the importance of applying a more comprehensive approach to carefully and thoroughly delineate which genes and pathways have the largest impact on variations in immunity to the current live rubella vaccine [19], [20]. The present work applies cutting-edge technology (mRNA-Seq) and sophisticated bioinformatics/statistical analyses to define transcriptional changes that characterize immune phenotypes following rubella vaccination.

## Materials and Methods

### Subjects

The methods described herein are similar or identical to those previously published by us [14]–[16], [18]. The recruitment of a large, population-based, age-stratified random sample of 738 healthy children and young adults, immunized with two doses of measles-mumps-rubella/MMR-II vaccine, (containing the Wistar RA 27/3-strain of rubella virus) was previously reported [14]–[16], [18]. Twenty-five study subjects representing the extremes of the humoral immune responses to rubella vaccine in this cohort (12 high antibody responders with a median titer of 138 IU/mL and 13 low responders with a median titer of 10 IU/mL) were selected for whole transcriptome mRNA-Seq profiling [21]. The subjects' peripheral blood mononuclear cells/PBMC samples (50 samples, 25 rubella virus-stimulated and 25 unstimulated samples) were randomized to balance immune response and stimulation status for cell culture setup, library preparation, and flow cell/lane run on the Illumina Genome Analyzer GAIIx instrument.

### Ethics statement

The Mayo Clinic Institutional Review Board granted approval for the study. Written, informed consent and assent (from minors) from subjects and/or parents/guardians was obtained at the time of enrollment [14]–[16], [18].

### Immune measures

Rubella-specific IgG antibody levels, rubella-specific IFNγ and IL10 Elispot measures, and secreted cytokines from stimulated PBMC cultures, were quantified as previously reported [16].

### PBMC culture, stimulation and total RNA extraction (isolation)

PBMC culture, stimulation and total RNA extraction were performed as described previously [21]. Subjects' PBMC were thawed and stimulated (or left unstimulated) with live rubella virus (W-Therien strain, a kind gift from Dr. Teryl Frey) at a multiplicity of infection/MOI = 5 for 48 hours. Total RNA was extracted from stabilized cells (RNAprotect cell reagent, Qiagen, Valencia, CA) using RNeasy Plus mini kit (Qiagen, Valencia, CA), as described previously [22], [23]. RNA concentration and quality were assessed by Nanodrop spectrophotometry (Thermo Fisher Scientific, Wilmington, DE) and Nano Chip kit analysis on an Agilent 2100 Bioanalyzer (Agilent, Palo Alto, CA), respectively. Fifty samples from 25 subjects were completed for culture, RNA extraction and RNA quality control. All samples successfully passed the RNA QA/QC with adequate concentration and purity (lack of DNA contamination), as well as good RNA integrity and lack of RNA degradation (RNA Integrity Number, RIN between 9 and 10).

### Library preparation and sequencing

Library preparation and sequencing were performed as described previously [21]. Briefly, libraries were prepared using the mRNA-Seq 8 Sample Prep Kit (Illimina, San Diego, CA) following the manufacturer's instructions and standard molecular biology techniques. Sample libraries were prepared in seven PCR batches. Polyadenylated RNA was isolated from 1 µg of total RNA using hybridization to oligo-dT magnetic beads for two rounds. The mRNA samples were chemically fragmented, reverse transcribed and converted into double-stranded cDNA. Unique Illumina adaptors (Illimina, San Diego, CA) were ligated to the DNA fragments after end repair (to produce blunt ends) and A-base tailing. Fragments of approximately 200 bp were gel purified and PCR enriched. The libraries were validated and quantified on an Agilent 2100 Bioanalyzer (Agilent, Palo Alto, CA) using the DNA 1000 Nano Chip kits.

Sequencing was carried out on a Genome Analyzer GAIIx (Illimina, San Diego, CA) using Illumina's proven sequencing by synthesis (SBS) technology. Libraries were loaded on the channels of the flow cell at 5–7 pM concentration. Samples were sequenced as single end reads using Illumina's Single Read Cluster Generation kit (v2) and 50 Cycle Illumina Sequencing Kit (v3) following the manufacturer's instructions. Flow cells were analyzed using Sequencing Control Studio (SCS) v 2.01 and SCS v 2.4. Each sample was sequenced in duplicate on two separate lanes within a single flow cell, which produced technical replicates for each sample; a total of 13 flow cells were used for sequencing.

The images from the sequencing cycles were processed using the Illumina Pipeline Software v1.5 using FireCrest, Bustard, ELAND (using genome build 36 and exon junction databases) and CASAVA. Full details on this methodology are provided elsewhere [21]. Viral replication was assessed using the Bowtie alignment tool to map mRNA-Seq reads to the rubella virus genome.

### Statistical methods

The statistical methods described below are similar or identical to those published in our mRNA-Seq methodology paper [21]. Specimens were randomly allocated to assay processing such that response and stimulation status were balanced over library preparation batch, flow cells and lanes. The primary endpoint used for differential expression was the number of reads (i.e., counts) per gene. Reads are defined as short fragments of cDNA representing the sample. Reads are sequenced and aligned back to the reference genome, and their number (aligned to a region) is an indication of the expression level. Minus versus average (MVA) and scatter plots were used to assess presence and functional form of global bias. Quantile-quantile (QQ) plots of Pearson goodness-of-fit statistics (assuming technical gene count variation follows a Poisson distribution) were created for each pair of technical replicates; the appropriate chi-square distribution was used for the expected quantiles.

Technical variation was found to be generally Poisson [21], thus, technical replicate gene counts were summed for analysis as has been done previously [24]. Generalized linear models (GLMs) [25] assuming the Negative Binomial distribution were utilized to assess statistical significance on a per-gene basis [21]. Empirical Bayes-like moderated estimates of the over-dispersion parameter were obtained using edgeR with n.prior = 3 [26] in R [27]. The 75% gene count per specimen was used as an offset in the generalized linear model in order to normalize and/or account for varying distributions of counts between specimens [28]. This results in the gene counts being interpreted as a rate with respect to the sequencing depth within that lane [29]. The full rationale supporting our modeling assumptions is described elsewhere [21]. The model contained indicator variables for high/low response status, unstimulated/stimulated status and the two-way interaction. Generalized estimating equations in SAS® (http://www.sas.com/) were used to test hypotheses to account for the correlation between paired specimens. We subset our gene expression analyses to genes having an average of at least five counts per sample due to model instability with fewer counts; genes with fewer counts were filtered out after computing the normalization factor. Since the objective of our study was to perform differential gene expression analyses between the groups of interest and in these analyses any per-gene corrections such as gene length cancel out in the test statistic, no per-gene adjustments were made. Gene expression was evaluated based on p-values and false discovery rate (FDR, to account for multiple comparisons)[30], [31], which were used to rank genes in order of significance. Self-contained gene set testing was performed using gene set definitions (G2 or second generation modules) based on other immune diseases [32], [33] and Fisher's method for combining p-values [34] together with restandardization [35]. Additional information on used gene sets (G2 modules), such as gene module content, annotation and functional interpretation is available at http://www.biir.net/public_wikis/module_annotation/V2_Trial_8_Modules [32], [33].

Pathway analysis was performed using both Ingenuity® Systems IPA software (Ingenuity Systems, Inc., Redwood City, CA), and the MetaCore™ software from GeneGo, Inc. (St. Joseph, MI).

## Results

### 1. Immune characteristics of the study subjects

To minimize potential confounding effects and variability, all selected study subjects were female non-Hispanic Caucasians with a median age at enrollment of 14 years (inter-quartile range/IQR 13, 16) and a median time since last rubella vaccination (blood draw) of 5.7 years (IQR 2.8, 7.1). The immune characteristics of the study subjects (representing the extremes of the humoral immune response to rubella vaccine out of 738 subjects) are summarized in Table 1. We observed a robust rubella-specific inflammatory cytokine response characterized by high levels of secreted IL-6, GM-CSF, and TNFα. We also detected low levels of Th1 cytokines IL-2 and IFNγ, while Th2 cytokines, IL-12p40 and Elispot responses were hardly detectable [17]. The median antibody level for the high antibody group of subjects was 138 IU/mL (IQR 121, 217) and the median antibody level for the low antibody group was 10 IU/mL (IQR 8, 11). Statistical analysis also demonstrated that the high antibody group secreted more rubella-specific IL-6 compared to the low antibody group (p = 0.002), while the differences among all other immune measures were not significant (Table 1).

**Table 1:** Immune characteristics of the study subjects.

ImmuneOutcome	Ab Response Category	Median (IQR)^a^	p-value^b^
Antibody (Ab)	High	138 (121, 217)	**<0.001**
	Low	10 (8, 11)	
IFNγ	High	12 (3, 27)	0.213
	Low	4 (1, 6)	
IL-10	High	6 (4, 7)	0.950
	Low	6 (4, 8)	
IL-2	High	14 (5, 24)	0.365
	Low	24 (11, 26)	
IL-6	High	3962 (3789, 4329)	**0.002**
	Low	3596 (3129, 3695)	
IL-4	High	0.1 (−0.2, 0.6)	0.604
	Low	0.3 (−0.3, 1.2)	
IL-5	High	0.5 (−0.2, 1.2)	0.544
	Low	0.8 (0.5, 0.9)	
IL-12p40	High	0 (−12, 8)	0.841
	Low	−6 (−8, 2)	
TNFα	High	64 (19, 115)	0.984
	Low	57 (25, 72)	
GM-CSF	High	32 (27, 37)	0.474
	Low	30 (27, 32)	
IFNγ Elispot	High	−2 (−9, 3)	0.649
	Low	−3 (−7, 0)	
IL10 Elispot	High	0 (−7, 4)	0.357
	Low	4 (−6, 9)	

a Values reported are in IU/mL for antibody responses, cytokine spot-forming units (SFUs) per 2×10^5^ cells for Elispot responses and pg/mL for secreted cytokines ± IQR, inter-quartile range with 25% and 75% quartiles.

Elispot response and secreted cytokine response is defined as the subject-specific median rubella virus-stimulated response (measured in triplicates) minus the median unstimulated response (also in triplicates). Negative values indicate that stimulated values were on average smaller than unstimulated values.

b P-value from multivariate analysis, statistically adjusting for all other variables (age at enrollment, age at first and second immunization, time since second rubella immunization to blood draw).

### 2. Overall gene expression in response to rubella virus stimulation (all stimulated vs. all unstimulated samples)

Sequencing failed for one high responder subject, and data failed QC for a second high responder subject, resulting in a final sample size of 10 high and 13 low responders to vaccination. 17,566 unique genes were detected with at least one count per gene per sample, and the total counts (total reads) per lane/sample ranged from 3.7 million to 10.7 million [21]. We evaluated overall host gene transcriptional changes after live rubella virus stimulation. More than 11,000 genes (11,167) had a p-value<0.05 and false discovery rate/FDR<0.07 for the test of upregulation or downregulation upon antigenic stimulation. Using a significance criterion more appropriate for a high dimensional study in order to limit the risk of false positives, we observed 1,080 differentially expressed genes with p<1.00E^−15^ (FDR<1.00E^−14^, Table S1). Table 2 demonstrates the results for 46 of those genes (42 upregulated genes and four downregulated genes), with the highest level of up/downregulation (differential expression) upon rubella virus stimulation (FC>4 or FC<0.25). These top host genes with substantial transcriptional changes included various immune function and host response/inflammation-related genes (such as *IL1B*, *CXCL6, CCL18, CCL23, SERPINE1, TREM1, GPR120, FN1, SLC39A8, NRP1, PROCR, SERPINA1, OLR1, ENPP2, SIGLEC11, TNFRSF12A, VSIG4, CLEC1A*), as well as other genes involved in cell signaling, cell regulation and transcription, and genes with unknown function (Table 2). Interestingly, most of these genes also displayed differences in up/downregulation between high and low antibody responders (Table 2).

**Table 2:** Overall response to rubella virus stimulation in PBMC samples of vaccines.

Gene symbol^a^	Gene description^a^	FC^b^	P-value^c^	FDR^c^	FC_low^d^	FC_high^e^
*MLXIPL*	MLX interacting protein-like, Williams-Beuren syndrome chromosomal region 14 protein	6.30	<1.0E-15	<1.0E-14	9.60	4.00
*CXCL6*	Chemokine (C-X-C motif) ligand 6;*a proinflammatory granulocyte chemotactic protein and activator with established role in innate immune defense*	11.25	<1.0E-15	<1.0E-14	7.73	19.47
*FAM135B*	Family with sequence similarity 135, member B	14.09	<1.0E-15	<1.0E-14	23.73	9.27
*PPP1R14C*	Protein phosphatase 1, regulatory (inhibitor) subunit 14C	4.34	<1.0E-15	<1.0E-14	5.32	3.32
*TNFAIP8L3*	Tumor necrosis factor, alpha-induced protein 8-like 3	5.75	<1.0E-15	<1.0E-14	7.24	4.31
*SHROOM4*	Shroom family member 4	8.92	<1.0E-15	<1.0E-14	11.76	6.38
*S1PR3*	Sphingosine-1-phosphate receptor 3, G protein-coupled receptor, endothelial differentiation gene-3	9.26	<1.0E-15	<1.0E-14	6.93	13.60
*RGNEF*	190 kDa guanine nucleotide exchange factor, Rho interacting protein 2	7.10	<1.0E-15	<1.0E-14	9.34	5.07
*FGD5*	FYVE, RhoGEF and PH domain containing 5	16.92	<1.0E-15	<1.0E-14	25.06	10.60
*ADAMDEC1*	ADAM-like, decysin 1, a disintegrin and metalloproteinase domain-like protein decysin-1; *a protein with a role in dendritic cell maturation and interaction with T cells*	0.14	<1.0E-15	<1.0E-14	0.11	0.18
*ARRDC4*	Arrestin domain containing 4	4.71	<1.0E-15	<1.0E-14	5.36	4.01
*DSC1*	Desmocollin 1, s a member of the desmocollin subfamily of the cadherin superfamily	0.16	<1.0E-15	<1.0E-14	0.19	0.13
*PKD2L1*	Polycystic kidney disease 2-like 1	0.16	<1.0E-15	<1.0E-14	0.13	0.19
*SGMS2*	Sphingomyelin synthase 2, SM synthase, phosphatidylcholine:ceramide cholinephosphotransferase 2	6.43	<1.0E-15	<1.0E-14	7.64	5.19
*CCL18*	Chemokine (C-C motif) ligand 18 (pulmonary and activation-regulated); *a protein with chemotactic activity for naïve T cells, CD4+ and CD8+ cells, and with role in both humoral and cell-mediated immune responses*	9.12	<1.0E-15	<1.0E-14	7.52	11.66
*ARMC9*	Armadillo repeat containing 9, NS21, armadillo/beta-catenin-like repeats, lisH domain-containing protein ARMC9	0.20	<1.0E-15	<1.0E-14	0.17	0.24
*DLC1*	Deleted in liver cancer 1, Rho-GTPase-activating protein 7	4.69	<1.0E-15	<1.0E-14	5.23	4.11
*SERPINE1*	Serpin peptidase inhibitor, clade E (nexin, plasminogen activator inhibitor type 1), member 1	12.65	<1.0E-15	<1.0E-14	15.82	9.61
*CCL23*	Chemokine (C-C motif) ligand 23, macrophage inflammatory protein 3, myeloid progenitor inhibitory factor 1; *a protein with chemotactic activity on monocytes and resting T cells*	16.41	<1.0E-15	<1.0E-14	19.04	13.48
*TREM1*	Triggering receptor expressed on myeloid cells 1, triggering receptor expressed on monocytes 1; triggering-receptor TREM1; *a protein, expressed on myeloid cells that increases the release of proinflammatory cytokines/chemokines and the expression of cell activation markers*	4.69	<1.0E-15	<1.0E-14	5.22	4.07
*TIMP4*	TIMP metallopeptidase inhibitor 4	6.60	<1.0E-15	<1.0E-14	7.60	5.53
*GPR120*	Omega-3 fatty acid receptor 1	9.12	<1.0E-15	<1.0E-14	10.80	7.43
*FAM20A*	Family with sequence similarity 20, member A	5.19	<1.0E-15	<1.0E-14	5.84	4.49
*SPINT1*	Serine peptidase inhibitor, Kunitz type	4.49	<1.0E-15	<1.0E-14	5.01	3.92
*PCOLCE2*	Procollagen C-endopeptidase enhancer 2,	17.28	<1.0E-15	<1.0E-14	20.29	13.92
*FN1*	Fibronectin 1, migration-stimulating factor; *a protein involved in cell adhesion, migration and host defense*	10.18	<1.0E-15	<1.0E-14	9.15	11.62
*SLC39A8*	Solute carrier family 39 (zinc transporter), member 8; *a zinc transporter found in membranes, that imports zinc during inflammation, recently shown to influence the expression of IFNγ in activated T cells*	4.34	<1.0E-15	<1.0E-14	4.65	3.98
*LYVE1*	Lymphatic vessel endothelial hyaluronan receptor 1	12.75	<1.0E-15	<1.0E-14	13.77	10.69
*PDPN*	Podoplanin	6.32	<1.0E-15	<1.0E-14	5.85	6.97
*TMTC1*	Transmembrane and tetratricopeptide repeat containing 1 [	4.21	<1.0E-15	<1.0E-14	4.07	4.41
*C12orf59*	Chromosome 12 open reading frame 59, uncharacterized protein C12orf59	9.54	<1.0E-15	<1.0E-14	10.39	8.55
*NRP1*	Neuropilin 1; *a protein supporting angiogenesis, cell migration, cell survival and cell attraction, involved in mediating the contact between dendritic cells and T cells*	4.68	<1.0E-15	<1.0E-14	5.00	4.30
*PROCR*	Protein C receptor, endothelial	4.64	<1.0E-15	<1.0E-14	4.93	4.31
*PPIC*	Peptidylprolyl isomerase C (cyclophilin C)	7.83	<1.0E-15	<1.0E-14	7.29	8.84
*SERPINA1*	Serpin peptidase inhibitor, clade A (alpha-1 antiproteinase, antitrypsin), member 1	6.44	<1.0E-15	<1.0E-14	6.78	6.04
*GNG12*	Guanine nucleotide binding protein (G protein), gamma 12	4.04	<1.0E-15	<1.0E-14	3.95	4.18
*OLR1*	Oxidized low density lipoprotein (lectin-like) receptor 1; *a protein involved in Fas-induced apoptosis*	9.90	<1.0E-15	<1.0E-14	9.98	9.81
*ENPP2*	Ectonucleotide pyrophosphatase/phosphodiesterase 2	6.17	<1.0E-15	<1.0E-14	6.11	6.24
*IL1B*	Interleukin 1, beta; *an important inflammatory mediator requiring inflammasome-mediated processing, involved in cell proliferation, cell differentiation and apoptosis*	5.94	<1.0E-15	<1.0E-14	5.97	5.91
*B3GNT5*	UDP-GlcNAc:betaGal beta-1,3-N-acetylglucosaminyltransferase 5	4.68	<1.0E-15	<1.0E-14	4.70	4.65
*SIGLEC11*	Sialic acid binding Ig-like lectin 11; *a mediator of anti-inflammatory/immunosuppressive signaling*	6.15	2.22E-16	3.40E-15	7.80	4.61
*TNFRSF12A*	Tumor necrosis factor receptor superfamily, member 12A; *initially recognized as a fibroblast growth factor inducible immediate early response gene; a receptor for TNF-like weak inducer of apoptosis/TWEAK with role in inflammation and systemic autoimmunity*	5.84	2.22E-16	3.40E-15	6.55	5.07
*VSIG4*	V-set and immunoglobulin domain containing 4; *a protein with immune regulatory functions, a negative regulator of T cell responses and a receptor for the complement component C3b*	6.22	2.22E-16	3.40E-15	6.89	5.47
*SPRED1*	Sprouty-related, EVH1 domain containing 1	5.05	4.44E-16	6.33E-15	5.73	4.33
*CLEC1A*	C-type lectin domain family 1, member A; *a protein with diverse functions in inflammation and immune response including cell adhesion, cell signaling and regulation of dendritic cell function*	4.39	6.66E-16	9.21E-15	4.90	3.82
*AQP9*	Aquaporin 9	8.53	8.88E-16	1.18E-14	10.30	6.75

a Gene symbol and gene description are provided for gene identification, information on immune function-related genes is provided in italic font.

b Fold change for overall response to stimulation analysis (all stimulated samples vs. all unstimulated samples; Stim/Unstim, S/U).

c P-value and false discovery rate (FDR) for the overall analysis.

d Fold change for High responders, stimulated vs. unstimulated samples (HS/HU).

e Fold change for Low responders, stimulated vs. unstimulated samples (LS/LU).

### 3. Response to viral stimulation in high vs. low antibody responders to rubella vaccination (interaction analysis)

**Figure 1:**
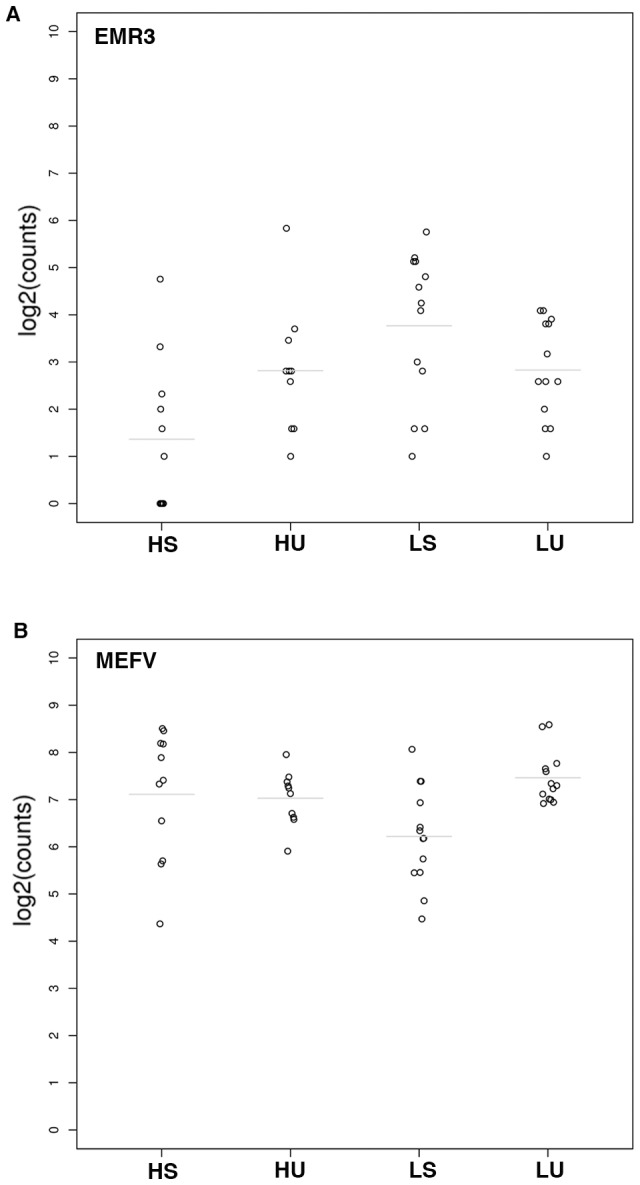
Dotplots of mRNA-Seq gene expression counts for A (EMR3, EGF-like module containing, mucin-like, gene) and B (MEFV, Mediterranean fever, gene), demonstrating differences in gene expression in high antibody responders compared to low antibody responders to rubella vaccination. Lines indicate the mean value of the counts within groups. Vertical axis is log_2_(gene counts). HU-gene counts for unstimulated PBMCs of high responders; HS-gene counts for rubella virus-stimulated PBMCs of high responders; LU-gene counts for unstimulated PBMCs of low responders; LS-gene counts for rubella virus-stimulated PBMCs of low responders.

We assessed and compared gene expression changes in response to *in vitro* viral stimulation in subjects representing the immune extremes of humoral immune responses following rubella vaccination. We identified 27 genes (p≤0.0006 and FDR≤0.30) that responded differently to viral stimulation in high vs. low antibody responders to rubella vaccine (Table 3). These genes included major histocompatibility complex (MHC) class I molecules (*HLA-A* and *HLA-B* with p = 0.0001 and p = 0.0005, respectively) and beta-2-microglobulin (*B2M*, p = 0.0002), other genes with unknown function and/or relation to immunity, and two genes related to innate immunity and inflammation (*EMR3* [EGF-like module-containing mucin-like hormone receptor-like 3 gene] and *MEFV* [Mediterranean fever gene] with p = 1.46E^−08^ and p = 0.0004 respectively, Table 3, Fig. 1A and 1B).

**Table 3:** Differential response to rubella virus stimulation in high vs. low antibody responders to rubella vaccination.

Gene symbol^a^	Gene description^a^	FC_int^b^	P-value_int^c^	FDR_int^c^	FC_low^d^	FC_high^e^
*EMR3*	EGF-like module containing, mucin-like, hormone receptor-like 3	0.18	1.46E-08	0.0002	2.29	0.41
*HIPK4*	Homeodomain interacting protein kinase 4	0.46	3.49E-07	0.002	1.46	0.67
*C11orf9*	Chromosome 11 open reading frame 9, myelin gene regulatory factor	0.76	2.40E-06	0.01	0.70	0.53
*RAMP1*	Receptor (G protein-coupled) activity modifying protein 1, calcitonin receptor-like receptor activity-modifying protein 1	0.67	2.49E-06	0.01	1.13	0.76
*RHEBL1*	Ras homolog enriched in brain like 1, GTPase RhebL1	1.57	7.53E-06	0.02	0.53	0.83
*TRPV6*	Transient receptor potential cation channel, subfamily V, member 6, calcium transport protein 1	0.45	3.05E-05	0.06	0.76	0.34
*MKRN1*	Makorin ring finger protein 1, E3 ubiquitin-protein ligase makorin-1; RING finger protein 61	1.19	3.17E-05	0.06	0.78	0.93
*NINJ1*	Ninjurin 1, nerve injury-induced protein 1	1.67	7.90E-05	0.14	0.58	0.97
*S100A13*	S100 calcium binding protein A13, S100 calcium-binding protein A13	0.52	9.89E-05	0.15	1.64	0.86
*HLA-A*	Major histocompatibility complex, class I, A	1.48	0.0001	0.15	0.59	0.87
*ASS1*	Argininosuccinate synthase 1	0.28	0.0002	0.20	1.45	0.41
*DLL1*	Delta-like 1 (Drosophila), delta-like protein 1; drosophila Delta homolog 1	2.13	0.0002	0.20	0.27	0.57
*MYL5*	Myosin, light chain 5, regulatory, myosin regulatory light chain 5	0.77	0.0002	0.21	1.14	0.87
*B2M*	Beta-2-microglobulin, beta chain of MHC class I molecules	1.30	0.0002	0.22	0.71	0.92
*WDR45L*	WDR45-like, WD repeat domain phosphoinositide-interacting protein 3	1.13	0.0003	0.26	0.90	1.02
*TRAPPC4*	trafficking protein particle complex 4, TRS23 homolog; hematopoietic stem/progenitor cell protein 172	1.15	0.0003	0.26	0.90	1.03
*TEAD3*	TEA domain family member 3, transcriptional enhancer factor 5; transcriptional enhancer factor TEF-5	0.55	0.0003	0.26	1.54	0.85
*TMEM176A*	Transmembrane protein 176A	2.26	0.0003	0.26	0.95	2.14
*ASAH1*	N-acylsphingosine amidohydrolase (acid ceramidase) 1	1.23	0.0004	0.26	0.80	0.98
*GLI3*	GLI family zinc finger 3, transcriptional activator GLI3; zinc finger protein GLI3	0.41	0.0004	0.26	1.26	0.52
*LOC401399*	PRRT4, proline-rich transmembrane protein 4	2.18	0.0004	0.27	0.52	1.13
*MEFV*	Mediterranean fever, FMF; MEF; TRIM20	2.72	0.0004	0.27	0.49	1.34
*HLA-B*	Major histocompatibility complex, class I, B, HLA class I histocompatibility antigen, B alpha chain	1.35	0.0005	0.29	0.78	1.06
*EGLN3*	egl nine homolog 3 (C. elegans), HIF prolyl hydroxylase 3	1.67	0.0005	0.29	0.66	1.11
*FLJ45422*	HLA-L, major histocompatibility complex, class I, L (pseudogene)	1.51	0.0005	0.29	0.71	1.07
*NWD1*	NACHT and WD repeat domain containing 1	0.22	0.0005	0.29	7.35	1.65
*TMEM176B*	Transmembrane protein 176B,	2.09	0.0006	0.29	0.83	1.73

a Gene symbol and gene description are provided for gene identification.

b Fold change for the interaction (HS/HU)/(LS/LU).

c P-value and false discovery rate for the interaction.

d Fold change in High responders, stimulated vs. unstimulated samples (HS/HU).

e Fold change in Low responders, stimulated vs. unstimulated samples (LS/LU).

### 4. Pathway analysis

We used both Ingenuity® software (http://www.ingenuity.com/) from Ingenuity® Systems (Redwood City, CA), and Metacore™ software (http://www.genego.com/metacore.php) from GeneGo, Inc.(St. Joseph, MI), which apply a competitive test that explores known canonical pathway maps to identify significant pathways with FDR<0.05, affected by the differentially expressed genes (in our data set), and found similar results. To explore overall changes after viral stimulation, we used the most significant 1,080 genes (p<1.00E^−15^, Table S1) as target genes. For the pathway analysis comparing response to viral stimulation between high vs. low antibody responders, we used the top 647 genes with p-value <0.05 (Table S2) as target genes. Table 4 lists the most significant overlapping pathways enriched after rubella virus stimulation with p-values using Ingenuity®. Two important immune pathways (“Antigen presentation pathway,” p = 1.21E^−04^ and “Complement system pathway,” including classical, alternative and lectin-induced complement cascades, p = 4.67E^−04^) were found to be enriched when comparing high vs. low antibody responders to rubella vaccination (Table 4).

**Table 4:** Significant pathways differentially expressed in rubella vaccine recipients.

No.	Pathway	P-value
	***Pathways enriched in overall analysis (all stimulated vs. all unstimulated samples)***	
1	Oxidative phosphorylation^a^	8.79E-08
	***Pathways enriched in interaction analysis (high vs. low antibody responders)***	
1	Antigen presentation pathway^a^	1.21E-04
2	Complement system pathway^a^	4.67E-04

a All presented pathways passed the FDR<0.05.

### 5. Gene set analysis

**Figure 2:**
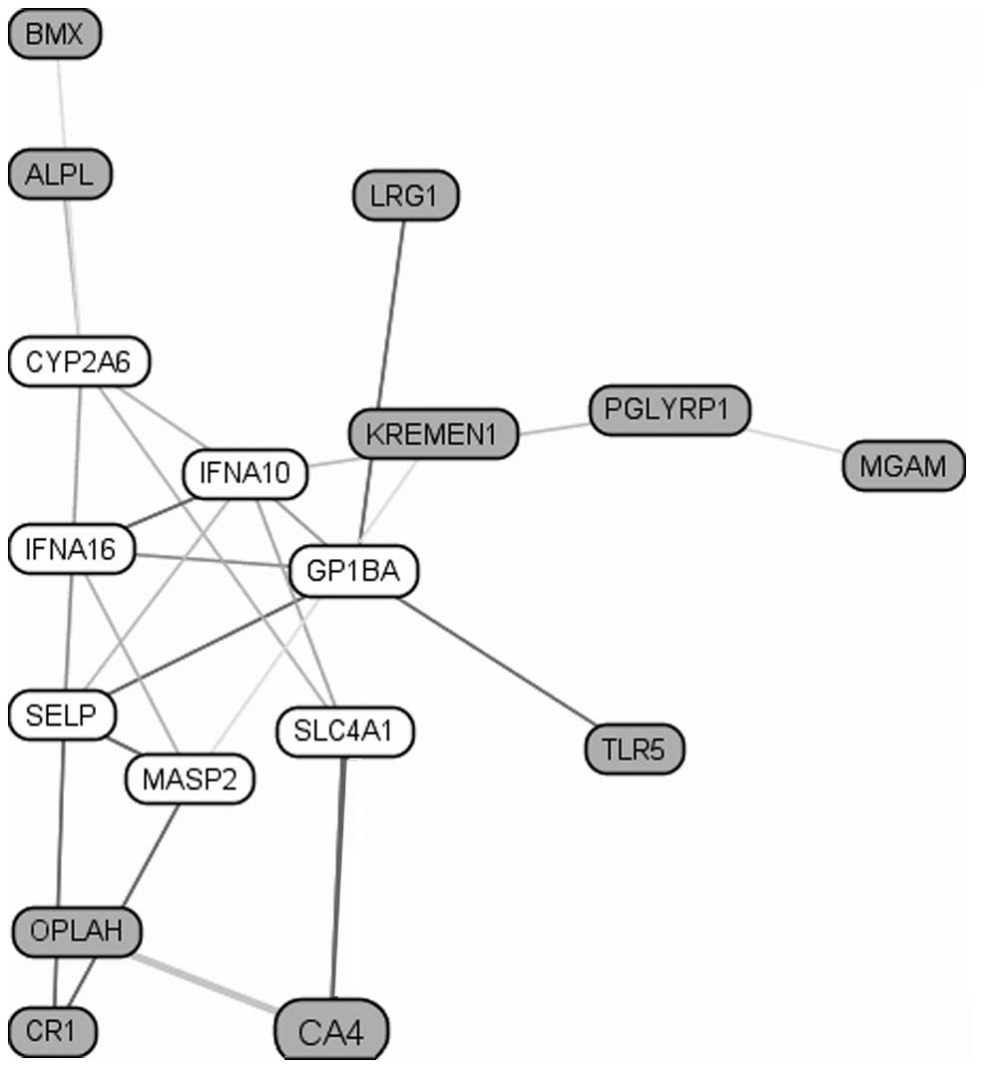
Local functional relationship networks of M4.2 (gene set, for gene annotation, please see Table 5) genes MGAM, ALPL, LOC728519, ANXA3, CR1, TLR5, CA4, BMX, PGLYRP1, OPLAH, LRG1, C19orf59, KREMEN1 for the context of Global Immune Network (ImmuNet tool http://tsb.mssm.edu/primeportal/?q=immuneNET). The functional relationship network was generated via Bayesian integration of diverse functional genomic data using a gold standard specific to immune system. The top 20 genes connected to the query set with connection weight higher than 0.339 are displayed. Darker lines indicate stronger functional relationships.

In addition to conventional pathway analysis, we also performed gene set tests using gene set modules reported by Chaussabel *et al.* [32], [33]. We applied a self-contained test by testing the hypothesis that a particular gene set as a whole is associated with immune response. Our analysis found two gene set modules that were marginally associated with a differential response to viral stimulation in high vs. low antibody responders (M4.2 and M8.101 with p-value of 0.07 and 0.08, respectively), and these gene sets also demonstrated a significant p-value in the overall analysis for response to viral stimulation (0.001 and 0.0007 for M4.2 and M8.101, respectively). Most of the genes comprising these two gene sets (Table 5) have diverse or unknown functions, although three genes were related to immune function (*TLR5*, *CR1* and *IL4I1*) and the M4.2 gene set is associated with inflammatory response [32], [33]. Functional relationships between the M4.2 genes are shown in Fig. 2 using the Global Immune Network/ImmuNet tool (http://tsb.mssm.edu/primeportal/?q=immuneNET).

**Table 5:** Gene sets with the highest significance based on the interaction model (p_1_) and overall gene expression (stimulated vs. unstimulated, p_2_) model.

Gene module	Gene	Gene description
	*LOC728519*	Similar to Baculoviral IAP repeat-containing protein 1, predicted neuronal apoptosis inhibitory protein (LOC728519)
	*ANXA3*	Annexin A3 (ANXA3)
	*TLR5*	Toll-like receptor 5 (TLR5)
	*KREMEN1*	Kringle containing transmembrane protein 1 (KREMEN1), transcript variant 4
	*CR1*	Complement component (3b/4b) receptor 1 (Knops blood group) (CR1), transcript variant S
	*BMX*	BMX non-receptor tyrosine kinase (BMX)
**M4.2**	*LRG1*	Leucine-rich alpha-2-glycoprotein 1 (LRG1)
p_1_ = 0.07^a^	*OPLAH*	5-oxoprolinase (ATP-hydrolysing) (OPLAH)
p_2_ = 0.001^b^	*ALPL*	Alkaline phosphatase, liver/bone/kidney (ALPL)
	*C19orf59*	Chromosome 19 open reading frame 59 (C19orf59)
	*LOC642112*	Similar to maltase-glucoamylase, intestinal (LOC642112), predicted
	*PGLYRP1*	Peptidoglycan recognition protein 1 (PGLYRP1)
	*CA4*	Carbonic anhydrase IV (CA4)
	*LOC642684*	Similar to maltase-glucoamylase, intestinal (LOC642684), predicted
	*MGAM*	Maltase-glucoamylase (alpha-glucosidase) (MGAM)
	*CYSLTR1*	Cysteinyl leukotriene receptor 1 (CYSLTR1)
	*DLST*	Dihydrolipoamide S-succinyltransferase (E2 component of 2-oxo-glutarate complex) (DLST)
	*C1orf124*	Chromosome 1 open reading frame 124 (C1orf124), transcript variant 2
**M8.101**	*TM2D3*	TM2 domain containing 3 (TM2D3), transcript variant 1
p_1_ = 0.08^a^	*C12orf49*	Chromosome 12 open reading frame 49 (C12orf49)
p_2_ = 0.0007^b^	*C1orf31*	Chromosome 1 open reading frame 31 (C1orf31)
	*LOC650034*	Similar to uncharacterized protein family UPF0227 member RGD1359682 (LOC650034), predicted
	*IL4I1*	Interleukin 4 induced 1 (IL4I1), transcript variant 2
	*KCTD5*	Potassium channel tetramerisation domain containing 5 (KCTD5)
	*LOC134145*	Hypothetical protein LOC134145 (LOC134145)
	*MED8*	Mediator of RNA polymerase II transcription, subunit 8 homolog (S. cerevisiae), (MED8) transcript variant 1, mRNA.

a p_1_ is the p-value from gene set-level analysis, analyzing interaction in high versus low antibody responders.

b p_2_ is the p-value from gene set-level analysis, analyzing overall all stimulated compared to all unstimulated samples.

### 6. Evaluation of viral gene expression in high vs. low responder

**Figure 3:**
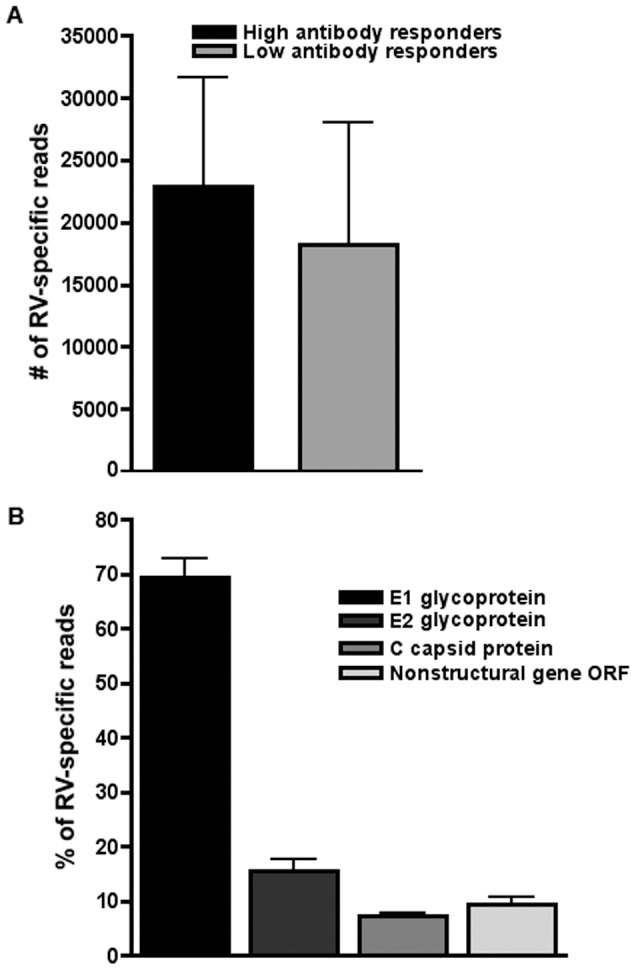
Analysis of mRNA-Seq reads/transcripts, mapping to Rubella virus genome. Quantification of viral transcripts in the high and low antibody responder groups was done using the Bowtie alignment tool, with alignment of reads to the Rubella virus strain Wistar RA 27/3, complete genome GenBank: FJ211588.1. **A** Mapping of rubella virus (RV)-specific reads in high antibody responders compared to low antibody responders to rubella vaccination; **B** Mapping of RV-specific reads across different rubella virus proteins. Bars represent mean ± SD.

In addition to host gene expression, mRNA-Seq technology allows simultaneous quantification of viral gene expression capacity in the cells of high and low antibody responders to rubella vaccination. As shown in Fig. 3A, the number of mRNA-Seq rubella virus-specific sequences was higher in the high antibody responders (mean 22,856 reads, 0.18% of all reads) compared to the low antibody responders (mean 18,263 reads, 0.14% of all reads), although this observed difference did not reach statistical significance (p = 0.08). Of the detected rubella virus-specific sequences, approximately 69% mapped to the E1 surface glycoprotein, 15% mapped to E2, 7% mapped to the capsid protein C and only 9% mapped to the rest of the genome (the nonstructural gene ORF) with no observed differences between high and low antibody responders (Fig. 3B).

## Discussion

The generation and maintenance of protective immunity to microorganisms, including natural infection and vaccine-induced immunity, is a complex process that entails transcriptional changes occurring in multiple interrelated genes, pathways and networks. Immune responses to vaccines are known to depend on demographic and clinical variables including age, gender, race, ethnicity and host genetic factors. We have previously determined that the heritability of rubella vaccine-induced antibody is high, reaching approximately 50% [6], and that the Human Leukocyte Antigen (HLA) loci explains only 19% of the overall genetic variation in rubella vaccine humoral immunity [12]. We have also demonstrated that immune responses to rubella vaccination are the result of multigenic influences and not a single dominant gene/allele model with the contribution of multiple immune genes important to the observed immune phenotype [11], [12], [14], [16], [18], [36]–[38]. In this study, we comprehensively assessed mRNA-Seq host transcriptome alterations in response to rubella virus and viral gene expression capacity in high and low rubella antibody vaccine responders and identified genes that were associated with antibody phenotypes at the extremes of the biological response.

Our overall analysis across all study subjects reveals novel and known immune genes with significant differential regulation after rubella virus stimulation (absolute FC above 4, Table 2). Our findings point to the transcriptional activation of genes involved mainly in innate and inflammatory immune responses and adaptive immune response priming. Only a few microarray studies have systematically examined rubella virus-induced host gene expression changes using human fetal and/or adult fibroblasts and human endothelial cells stimulated with different rubella virus strains, and they report differential expression of genes related to innate/inflammatory and immune response such as interferon (IFN) and IFN-stimulated genes with antiviral and immunoregulatory activities, HLA genes, cytokines, chemokines and genes associated with apoptosis [39], [40]. Several interesting immunity-related genes from our transcriptomics data in human peripheral blood mononuclear cells (*NRP1, SLC39A8*, *ARMC9, CXCL6*) were found to be differentially expressed in the same direction (all upregulated with the exception of *ARMC9*), as in the study by Adamo *et al.* in human fibroblasts [39]. Neuropilin-1 (*NRP1*) encodes a protein supporting angiogenesis, cell migration, cell survival and cell attraction. This protein is also reported to mediate the contact between dendritic cells and T cells to instigate primary immune responses [41], while solute carrier family 39 member 8 (*SLC39A8*) encodes a zinc transporter found in the plasma membrane, mitochondria and lysosome, that imports zinc during inflammation and was recently shown to influence the expression of IFNγ in activated T cells [42]. Chemokine (C-X-C motif) ligand 6 (*CXCL6*, upregulated more than 11-fold in our data set, Table 2), is a pro-inflammatory chemotactic protein for neutrophils with established innate immune defense and antibacterial activities [43], whereas the function of the armadillo repeat containing 9 (*ARMC9*) gene is still unknown. The discovery of these differentially expressed genes, with a plausible relation to host response and adaptive immunity in two independent rubella gene expression studies, points to the high likelihood that they may indeed account for rubella virus-induced immune activation, including transcriptional activation induced by live rubella virus vaccine.

To elucidate the genetic basis for the observed heterogeneity in immune responses, our mRNA-Seq analysis compared transcriptional patterns observed in high and low antibody responders to rubella vaccination, identifying 27 genes with significantly different expression between the studied immune phenotypic extremes (high and low/non-protective antibody response). The identified class I major histocompatibility complex genes (*HLA-A* and *HLA-B*; p-value = 0.0001 and p-value = 0.0005,respectively, Table 3), and the beta-2-microglobulin gene (*B2M*, p-value = 0.0002) are historically known for their role in cell-mediated and antiviral immunity by presenting peptides derived from the endoplasmic reticulum to cytotoxic T cells (CTLs), but their relation to humoral immunity is unclear. We have previously demonstrated and replicated independent HLA class I associations, particularly B*2705 (p-value<0.001), class I B (B27) supertype associations (p = 0.008), as well as extended HLA class I-class II A-C-B-LTA-TNF-LST1-DRB1-DQA1-DQB1-DPA1-DPB1 haplotype (A*02-C*03-B*15-AAAACGGGGC-DRB1*04-DQA1*03-DQB1*03-DPA1*01-DPB1*04, p = 0.002) associations with rubella vaccine-induced antibody responses [12], [44], [45], which clearly shows that polymorphisms in the MHC class I locus influence humoral immunity, although the molecular mechanisms have yet to be delineated. Our current findings, using mRNA-Seq global gene expression of immune extreme phenotypes, further strengthen the evidence for the effect of MHC class I genes in the observed rubella vaccine-induced immune response variations.

The top gene differentially expressed in high versus low antibody responders in our study, *EMR3* (p = 1.46E^−08^, FDR = 0.0002, Table 3), is an epidermal growth factor-seven transmembrane receptor family (EGF-TM7) member, expressed predominantly on granulocytes, monocytes and macrophages. This gene was significantly downregulated (FC = 0.41, Fig. 1A) in high antibody vaccine responders and significantly upregulated (FC = 2.29, Fig. 1A) in low antibody vaccine responders, and may be a factor involved in cell migration, “cross-talk” between myeloid cells, and modulation of inflammatory and immune responses [46], [47]. Another intriguing finding is the identification of the gene MEFV responsible for the most common inheritable fever syndrome, familial Mediterranean fever (characterized by recurrent fever and inflammation). The *MEFV* gene on chromosome 16p13, was associated with differential gene expression between high and low antibody responders in our study (p = 0.0004, FDR = 0.27; gene expression upregulated in high responders, FC = 1.34, and downregulated in low responders, FC = 0.49; Table 3, Fig. 1B). The encoded protein, pyrin, is a central immune modulator of innate and inflammatory response by regulation of inflammasome activation and/or direct activation of inflammatory pathways [48], and therefore is a highly plausible genetic regulator of inflammatory response induced by live rubella vaccine with an impact on subsequent vaccine-induced adaptive immunity.

As the immune response is a multifaceted, dynamic entity and is not dominated by single gene effects, we assessed and identified canonical pathways and gene sets associated with rubella virus stimulation in vaccinees, and particularly functionally different pathways and gene sets (inferred from the differences in gene expression) in high compared to low antibody responders. Two classical canonical pathways that played definitive roles in initiation/regulation of adaptive immune response and in innate antigen-nonspecific defense, the antigen presentation pathway (p = 1.21E^−04^, Table 4) and the complement system pathway (p = 4.67E^−04^, Table 4), had a FDR<0.05 and were considered to be enriched pathways that discriminated between immune extreme antibody phenotypes in our study. Additional insights into the functional relationships between genes and gene expression networks were gained using an innovative gene set analysis approach, assessing 260 gene sets (G2 modules) [32], [33]. This analysis, which better reflects the functional relationship networks between genes and the underlying biology, identified two rubella vaccine response-specific transcriptional gene sets (M4.2 and M8.101, Table 5) that were significantly associated with immune response to rubella virus stimulation and were marginally associated with gene expression differences between the examined immune extreme antibody phenotypes. Most of the individual genes comprising these gene sets have unknown function or function unrelated to antiviral immune response. The toll-like receptor (TLR) family member 5 (*TLR5*, in M4.2) is an important innate pathogen recognition receptor that is involved in the recognition of bacterial flagellin, and is also reported to trigger reactivation of latent HIV-1 infection and activate virus gene expression in T cells [49]. Human IL4I1 (in M8.101) has been reported as a new immunomodulatory enzyme expressed by dendritic cells that is able to inhibit T cell proliferation and thus may directly influence the immune response [50].While the annotation of M8.101 is still unknown, the M4.2 transcriptional pattern has been annotated as an inflammation-related gene set based on its GeneGo, GoStat, and IPA analyses and is linked to “inflammation, cell movement of leukocytes, phagocytes, and granulocytes (IPA) and innate immune response (GoStat)” [32], [33].

Our assessment of host transcriptional changes through blood transciptomics revealed that inflammatory genes and genes related to an early phase of immune response (antigen presentation) are important in typifying and characterizing high and low antibody response to rubella vaccination. Inflammation is the first line of host defense in response to infection, and is characterized by local physiological changes and the release of cytokines/chemokines and other mediators that promote cell interactions, chemotaxis and recruitment of immune cells for priming and orchestration of adaptive immunity [51]. A population-based profiling of cytokine responses in rubella vaccine recipients demonstrated a robust virus-specific inflammatory response rather than Th1/Th2 cytokine patterns 5.7 years after the last (second) immunization, suggesting that longstanding antiviral responses and immune variations may be regulated by inflammatory cytokines [17].

The strengths of our study involve the use of cutting-edge genome-wide technology for simultaneous transcriptional profiling of host and viral gene expression [52] and the application of innovative and appropriate statistical modeling/analysis methodology to assess clinically relevant vaccine-induced immune response phenotypes. As reported in our previous study [53] and acknowledged by others, Next Generation Sequencing is an avant-garde technology with high sensitivity and reproducibility for transcriptional profiling and excellent correlation of differential gene expression with real-time quantitative PCR. However, further replication studies and biological/functional validation of identified gene targets are warranted to confirm our findings and are in progress. There might be a concern that some of the observed estimates of the fold change were relatively small (although consistent between samples) and the thresholds for significance were not stringent enough. Reviews from the current statistical literature consider FDR rates of 10–20% as acceptable for differential expression [54]. In our study we have tried to be more inclusive, rather than miss biologically important information, and have used FDR of up to 30% (in the differential expression analysis comparing high vs. low antibody responders). Our findings are in concert with what is seen when evaluating PBMCs, which contain a variety of cell types and the observed responses may represent a large response in one cell type and no response in another. In addition, small FC in gene expression of key regulatory genes (such as transcription factors) may have disproportionally large functional downstream effects. Finally, biological processes are rarely driven by single genes, but rather by a collection of genes, pathways and gene networks, where small differences in multiple genes may cumulatively have a larger or magnified effect.

In summary, our analysis is the first global mRNA-Seq gene expression profiling after rubella vaccination that presents evidence for unique quantitative transcriptional differences between high and low antibody responders to rubella vaccination. These transcriptional differences and the underlying mechanisms are crucial to understanding the basis of vaccine immune responses, and for development of novel and improved vaccines.

## Supporting Information

Table S1 **Overall response to rubella virus stimulation in PBMC samples of vaccinees (top 1,080 genes).**
(DOC)

Table S2 **Response to rubella virus stimulation in high vs. low antibody responders to rubella vaccination (genes with p<0.05).**
(DOCX)
