# Supplementary material for: Genome-Wide Characterization of Transcriptional Patterns in High and Low Antibody Responders to Rubella Vaccination
Source: PLoS One. 2013 May 1;8(5):e62149. doi: 10.1371/journal.pone.0062149 (PMC3641062; doi:10.1371/journal.pone.0062149)
Supplement: Table S1 — Overall response to rubella virus stimulation in PBMC samples of vaccinees (top 1,080 genes). (DOC) [file pone.0062149.s001.doc]

**Supplemental Table 1**. Overall response to rubella virus stimulation in PBMC samples of vaccinees (top 1,080 genes)

| **Gene symbol**^a^ | **FC**^b^ | **P-value**^c^ | **FDR**^c^ | **FC_low**^d^ | **FC_high**^e^ |
| --- | --- | --- | --- | --- | --- |
| *VCAN* | 3.76 | <1.00E-16 | <1.00E-15 | 4.86 | 2.77 |
| *BAALC* | 0.33 | <1.00E-16 | <1.00E-15 | 0.26 | 0.44 |
| *MLXIPL* | 6.3 | <1.00E-16 | <1.00E-15 | 9.6 | 4 |
| *DPF2* | 0.77 | <1.00E-16 | <1.00E-15 | 0.73 | 0.83 |
| *CCR6* | 0.63 | <1.00E-16 | <1.00E-15 | 0.57 | 0.72 |
| *ZNF823* | 0.57 | <1.00E-16 | <1.00E-15 | 0.5 | 0.66 |
| *NLRP2* | 1.93 | <1.00E-16 | <1.00E-15 | 2.22 | 1.62 |
| *TAGAP* | 0.6 | <1.00E-16 | <1.00E-15 | 0.54 | 0.68 |
| *RNF34* | 0.8 | <1.00E-16 | <1.00E-15 | 0.76 | 0.84 |
| *PDXDC1* | 0.8 | <1.00E-16 | <1.00E-15 | 0.76 | 0.85 |
| *ST14* | 3.18 | <1.00E-16 | <1.00E-15 | 2.71 | 3.89 |
| *HIVEP3* | 2.91 | <1.00E-16 | <1.00E-15 | 3.66 | 2.19 |
| *PPFIBP1* | 2.51 | <1.00E-16 | <1.00E-15 | 3.02 | 2 |
| *LACTB2* | 1.4 | <1.00E-16 | <1.00E-15 | 1.32 | 1.52 |
| *SETD7* | 1.53 | <1.00E-16 | <1.00E-15 | 1.67 | 1.38 |
| *GIMAP6* | 0.73 | <1.00E-16 | <1.00E-15 | 0.69 | 0.79 |
| *CXCL6* | 11.25 | <1.00E-16 | <1.00E-15 | 7.73 | 19.47 |
| *RNFT1* | 1.45 | <1.00E-16 | <1.00E-15 | 1.56 | 1.33 |
| *SERPINF1* | 0.46 | <1.00E-16 | <1.00E-15 | 0.4 | 0.55 |
| *MCM2* | 0.69 | <1.00E-16 | <1.00E-15 | 0.64 | 0.75 |
| *FAM135B* | 14.09 | <1.00E-16 | <1.00E-15 | 23.73 | 9.27 |
| *NAIF1* | 0.73 | <1.00E-16 | <1.00E-15 | 0.7 | 0.78 |
| *CYP2U1* | 0.59 | <1.00E-16 | <1.00E-15 | 0.53 | 0.67 |
| *FAT4* | 0.41 | <1.00E-16 | <1.00E-15 | 0.48 | 0.34 |
| *CECR1* | 0.61 | <1.00E-16 | <1.00E-15 | 0.56 | 0.69 |
| *C12orf45* | 1.55 | <1.00E-16 | <1.00E-15 | 1.67 | 1.41 |
| *XPO6* | 0.79 | <1.00E-16 | <1.00E-15 | 0.76 | 0.83 |
| *KIR3DL1* | 0.36 | <1.00E-16 | <1.00E-15 | 0.41 | 0.3 |
| *GIMAP5* | 0.73 | <1.00E-16 | <1.00E-15 | 0.69 | 0.78 |
| *PPP1R14C* | 4.34 | <1.00E-16 | <1.00E-15 | 5.32 | 3.32 |
| *HIP1* | 2.37 | <1.00E-16 | <1.00E-15 | 2.77 | 1.96 |
| *C17orf67* | 0.39 | <1.00E-16 | <1.00E-15 | 0.33 | 0.46 |
| *TAF8* | 0.77 | <1.00E-16 | <1.00E-15 | 0.73 | 0.81 |
| *SLC25A40* | 1.86 | <1.00E-16 | <1.00E-15 | 2.04 | 1.65 |
| *PARP8* | 0.6 | <1.00E-16 | <1.00E-15 | 0.55 | 0.66 |
| *MGLL* | 0.49 | <1.00E-16 | <1.00E-15 | 0.44 | 0.57 |
| *GIMAP7* | 0.65 | <1.00E-16 | <1.00E-15 | 0.61 | 0.71 |
| *C12orf5* | 1.65 | <1.00E-16 | <1.00E-15 | 1.78 | 1.5 |
| *COL4A3* | 0.47 | <1.00E-16 | <1.00E-15 | 0.53 | 0.41 |
| *TNFAIP8L3* | 5.75 | <1.00E-16 | <1.00E-15 | 7.24 | 4.31 |
| *SHROOM4* | 8.92 | <1.00E-16 | <1.00E-15 | 11.76 | 6.38 |
| *PHF16* | 0.61 | <1.00E-16 | <1.00E-15 | 0.56 | 0.67 |
| *B4GALNT3* | 0.44 | <1.00E-16 | <1.00E-15 | 0.47 | 0.39 |
| *KIAA1166* | 0.63 | <1.00E-16 | <1.00E-15 | 0.59 | 0.68 |
| *S1PR3* | 9.26 | <1.00E-16 | <1.00E-15 | 6.93 | 13.6 |
| *C1QTNF6* | 0.57 | <1.00E-16 | <1.00E-15 | 0.61 | 0.52 |
| *SNAI3* | 2.09 | <1.00E-16 | <1.00E-15 | 2.37 | 1.8 |
| *TMCO6* | 0.61 | <1.00E-16 | <1.00E-15 | 0.56 | 0.68 |
| *LOC170082* | 0.67 | <1.00E-16 | <1.00E-15 | 0.63 | 0.72 |
| *HERPUD2* | 0.79 | <1.00E-16 | <1.00E-15 | 0.76 | 0.83 |
| *HLA-DRB5* | 2.36 | <1.00E-16 | <1.00E-15 | 2.09 | 2.76 |
| *PRR17* | 0.56 | <1.00E-16 | <1.00E-15 | 0.64 | 0.46 |
| *ASPRV1* | 0.52 | <1.00E-16 | <1.00E-15 | 0.47 | 0.59 |
| *DHRS12* | 0.62 | <1.00E-16 | <1.00E-15 | 0.58 | 0.67 |
| *RSPH3* | 1.67 | <1.00E-16 | <1.00E-15 | 1.55 | 1.84 |
| *RGNEF* | 7.1 | <1.00E-16 | <1.00E-15 | 9.34 | 5.07 |
| *SEL1L* | 1.49 | <1.00E-16 | <1.00E-15 | 1.57 | 1.39 |
| *KIAA1598* | 1.85 | <1.00E-16 | <1.00E-15 | 1.72 | 2.04 |
| *WIPF1* | 0.74 | <1.00E-16 | <1.00E-15 | 0.71 | 0.78 |
| *ACRBP* | 0.53 | <1.00E-16 | <1.00E-15 | 0.49 | 0.6 |
| *FGD5* | 16.92 | <1.00E-16 | <1.00E-15 | 25.06 | 10.6 |
| *IHPK2* | 0.74 | <1.00E-16 | <1.00E-15 | 0.7 | 0.78 |
| *PTPRN2* | 0.56 | <1.00E-16 | <1.00E-15 | 0.52 | 0.61 |
| *HAVCR2* | 1.95 | <1.00E-16 | <1.00E-15 | 1.77 | 2.2 |
| *PPIL1* | 1.55 | <1.00E-16 | <1.00E-15 | 1.65 | 1.42 |
| *FHL3* | 2.23 | <1.00E-16 | <1.00E-15 | 2.52 | 1.92 |
| *TJP1* | 3.81 | <1.00E-16 | <1.00E-15 | 4.59 | 3.06 |
| *SIN3A* | 0.73 | <1.00E-16 | <1.00E-15 | 0.7 | 0.77 |
| *ATP9A* | 0.49 | <1.00E-16 | <1.00E-15 | 0.54 | 0.43 |
| *P2RY1* | 2.82 | <1.00E-16 | <1.00E-15 | 3.19 | 2.41 |
| *IFT57* | 0.63 | <1.00E-16 | <1.00E-15 | 0.59 | 0.68 |
| *PAK6* | 0.44 | <1.00E-16 | <1.00E-15 | 0.48 | 0.41 |
| *MYH7B* | 0.48 | <1.00E-16 | <1.00E-15 | 0.53 | 0.43 |
| *MBOAT2* | 1.77 | <1.00E-16 | <1.00E-15 | 1.64 | 1.95 |
| *PAQR8* | 0.55 | <1.00E-16 | <1.00E-15 | 0.51 | 0.61 |
| *GLT25D2* | 0.53 | <1.00E-16 | <1.00E-15 | 0.57 | 0.48 |
| *PIP3-E* | 0.55 | <1.00E-16 | <1.00E-15 | 0.51 | 0.6 |
| *DDX24* | 0.73 | <1.00E-16 | <1.00E-15 | 0.7 | 0.77 |
| *C17orf53* | 0.47 | <1.00E-16 | <1.00E-15 | 0.44 | 0.52 |
| *PNRC1* | 0.64 | <1.00E-16 | <1.00E-15 | 0.61 | 0.69 |
| *FN3K* | 0.61 | <1.00E-16 | <1.00E-15 | 0.65 | 0.57 |
| *ING4* | 0.6 | <1.00E-16 | <1.00E-15 | 0.56 | 0.65 |
| *DDHD1* | 0.7 | <1.00E-16 | <1.00E-15 | 0.67 | 0.74 |
| *NDUFS1* | 1.46 | <1.00E-16 | <1.00E-15 | 1.52 | 1.38 |
| *ADAMDEC1* | 0.14 | <1.00E-16 | <1.00E-15 | 0.11 | 0.18 |
| *RRM2* | 0.48 | <1.00E-16 | <1.00E-15 | 0.44 | 0.54 |
| *TGFA* | 2.2 | <1.00E-16 | <1.00E-15 | 2.39 | 1.99 |
| *HLA-DQB1* | 1.81 | <1.00E-16 | <1.00E-15 | 1.69 | 1.98 |
| *C19orf46* | 0.45 | <1.00E-16 | <1.00E-15 | 0.49 | 0.4 |
| *C9orf46* | 1.53 | <1.00E-16 | <1.00E-15 | 1.45 | 1.63 |
| *PHF15* | 0.58 | <1.00E-16 | <1.00E-15 | 0.54 | 0.63 |
| *TBC1D1* | 1.69 | <1.00E-16 | <1.00E-15 | 1.8 | 1.56 |
| *KIAA1841* | 1.61 | <1.00E-16 | <1.00E-15 | 1.68 | 1.52 |
| *HJURP* | 0.5 | <1.00E-16 | <1.00E-15 | 0.46 | 0.55 |
| *TIFAB* | 0.29 | <1.00E-16 | <1.00E-15 | 0.25 | 0.35 |
| *PGM2* | 2.04 | <1.00E-16 | <1.00E-15 | 2.2 | 1.87 |
| *STK3* | 1.89 | <1.00E-16 | <1.00E-15 | 1.77 | 2.05 |
| *RRP12* | 2.02 | <1.00E-16 | <1.00E-15 | 2.2 | 1.81 |
| *RAPH1* | 2.31 | <1.00E-16 | <1.00E-15 | 2.53 | 2.05 |
| *ZRSR2* | 0.77 | <1.00E-16 | <1.00E-15 | 0.79 | 0.73 |
| *POU2AF1* | 0.56 | <1.00E-16 | <1.00E-15 | 0.52 | 0.61 |
| *KRTCAP3* | 0.57 | <1.00E-16 | <1.00E-15 | 0.61 | 0.52 |
| *ACCS* | 0.51 | <1.00E-16 | <1.00E-15 | 0.47 | 0.55 |
| *STON2* | 3.63 | <1.00E-16 | <1.00E-15 | 4.2 | 3.03 |
| *TBL2* | 1.71 | <1.00E-16 | <1.00E-15 | 1.82 | 1.58 |
| *ARL4C* | 0.38 | <1.00E-16 | <1.00E-15 | 0.34 | 0.44 |
| *ACRC* | 0.49 | <1.00E-16 | <1.00E-15 | 0.52 | 0.45 |
| *DOK7* | 0.5 | <1.00E-16 | <1.00E-15 | 0.53 | 0.46 |
| *CLTC* | 1.36 | <1.00E-16 | <1.00E-15 | 1.41 | 1.3 |
| *LETMD1* | 0.68 | <1.00E-16 | <1.00E-15 | 0.65 | 0.72 |
| *KCNE3* | 1.72 | <1.00E-16 | <1.00E-15 | 1.62 | 1.86 |
| *STARD3NL* | 1.44 | <1.00E-16 | <1.00E-15 | 1.39 | 1.5 |
| *PRKAR1A* | 1.29 | <1.00E-16 | <1.00E-15 | 1.33 | 1.24 |
| *CADM1* | 0.42 | <1.00E-16 | <1.00E-15 | 0.38 | 0.48 |
| *FAM153A* | 0.36 | <1.00E-16 | <1.00E-15 | 0.4 | 0.32 |
| *BHLHB2* | 1.71 | <1.00E-16 | <1.00E-15 | 1.8 | 1.61 |
| *COLQ* | 0.55 | <1.00E-16 | <1.00E-15 | 0.51 | 0.59 |
| *PRF1* | 0.43 | <1.00E-16 | <1.00E-15 | 0.39 | 0.48 |
| *TMEM169* | 0.52 | <1.00E-16 | <1.00E-15 | 0.56 | 0.48 |
| *KLHDC8B* | 0.34 | <1.00E-16 | <1.00E-15 | 0.31 | 0.39 |
| *NDUFAF1* | 1.29 | <1.00E-16 | <1.00E-15 | 1.33 | 1.25 |
| *CERKL* | 0.49 | <1.00E-16 | <1.00E-15 | 0.46 | 0.55 |
| *WHDC1* | 0.77 | <1.00E-16 | <1.00E-15 | 0.75 | 0.8 |
| *SLC38A6* | 1.92 | <1.00E-16 | <1.00E-15 | 1.82 | 2.05 |
| *ARRDC4* | 4.71 | <1.00E-16 | <1.00E-15 | 5.36 | 4.01 |
| *CPNE1* | 0.75 | <1.00E-16 | <1.00E-15 | 0.73 | 0.78 |
| *GPNMB* | 0.4 | <1.00E-16 | <1.00E-15 | 0.36 | 0.45 |
| *LOC158830* | 0.48 | <1.00E-16 | <1.00E-15 | 0.52 | 0.44 |
| *DSC1* | 0.16 | <1.00E-16 | <1.00E-15 | 0.19 | 0.13 |
| *PKD2L1* | 0.16 | <1.00E-16 | <1.00E-15 | 0.13 | 0.19 |
| *APOC2* | 3.86 | <1.00E-16 | <1.00E-15 | 3.37 | 4.58 |
| *TMEM167A* | 1.61 | <1.00E-16 | <1.00E-15 | 1.68 | 1.52 |
| *MST1* | 0.57 | <1.00E-16 | <1.00E-15 | 0.61 | 0.54 |
| *PCBD1* | 1.78 | <1.00E-16 | <1.00E-15 | 1.68 | 1.92 |
| *SMYD4* | 0.66 | <1.00E-16 | <1.00E-15 | 0.64 | 0.7 |
| *NSF* | 1.22 | <1.00E-16 | <1.00E-15 | 1.2 | 1.26 |
| *CDCA7L* | 0.58 | <1.00E-16 | <1.00E-15 | 0.55 | 0.62 |
| *CARD11* | 0.54 | <1.00E-16 | <1.00E-15 | 0.51 | 0.58 |
| *BHLHB9* | 0.63 | <1.00E-16 | <1.00E-15 | 0.65 | 0.59 |
| *LAMA5* | 0.56 | <1.00E-16 | <1.00E-15 | 0.6 | 0.52 |
| *PIR* | 1.71 | <1.00E-16 | <1.00E-15 | 1.65 | 1.8 |
| *BRD2* | 0.76 | <1.00E-16 | <1.00E-15 | 0.74 | 0.79 |
| *ARID3B* | 0.61 | <1.00E-16 | <1.00E-15 | 0.58 | 0.65 |
| *HSPC111* | 1.56 | <1.00E-16 | <1.00E-15 | 1.63 | 1.47 |
| *C20orf12* | 0.63 | <1.00E-16 | <1.00E-15 | 0.66 | 0.6 |
| *MIZF* | 0.71 | <1.00E-16 | <1.00E-15 | 0.69 | 0.74 |
| *PHF1* | 0.56 | <1.00E-16 | <1.00E-15 | 0.52 | 0.6 |
| *CPM* | 2.22 | <1.00E-16 | <1.00E-15 | 2.06 | 2.46 |
| *ZNF8* | 0.69 | <1.00E-16 | <1.00E-15 | 0.67 | 0.73 |
| *ARHGAP25* | 0.57 | <1.00E-16 | <1.00E-15 | 0.54 | 0.6 |
| *SGMS2* | 6.43 | <1.00E-16 | <1.00E-15 | 7.64 | 5.19 |
| *CAMSAP1L1* | 1.9 | <1.00E-16 | <1.00E-15 | 2.04 | 1.74 |
| *CD200* | 0.55 | <1.00E-16 | <1.00E-15 | 0.52 | 0.59 |
| *PLCD4* | 0.62 | <1.00E-16 | <1.00E-15 | 0.59 | 0.65 |
| *CCL18* | 9.12 | <1.00E-16 | <1.00E-15 | 7.52 | 11.66 |
| *C7orf24* | 1.97 | <1.00E-16 | <1.00E-15 | 2.09 | 1.83 |
| *CDCA5* | 0.55 | <1.00E-16 | <1.00E-15 | 0.51 | 0.59 |
| *CYCS* | 1.94 | <1.00E-16 | <1.00E-15 | 2.05 | 1.81 |
| *ARMC9* | 0.2 | <1.00E-16 | <1.00E-15 | 0.17 | 0.24 |
| *BTN3A2* | 0.63 | <1.00E-16 | <1.00E-15 | 0.61 | 0.66 |
| *SIPA1L1* | 0.65 | <1.00E-16 | <1.00E-15 | 0.62 | 0.69 |
| *RGMB* | 0.5 | <1.00E-16 | <1.00E-15 | 0.46 | 0.54 |
| *ZNF394* | 0.72 | <1.00E-16 | <1.00E-15 | 0.7 | 0.75 |
| *DLC1* | 4.69 | <1.00E-16 | <1.00E-15 | 5.23 | 4.11 |
| *UBXD6* | 1.58 | <1.00E-16 | <1.00E-15 | 1.64 | 1.51 |
| *ADAM28* | 0.33 | <1.00E-16 | <1.00E-15 | 0.3 | 0.38 |
| *MED26* | 0.81 | <1.00E-16 | <1.00E-15 | 0.79 | 0.83 |
| *C6orf173* | 2.26 | <1.00E-16 | <1.00E-15 | 2.09 | 2.5 |
| *ATP6V1E1* | 1.41 | <1.00E-16 | <1.00E-15 | 1.37 | 1.47 |
| *SLC40A1* | 0.34 | <1.00E-16 | <1.00E-15 | 0.3 | 0.38 |
| *BRUNOL6* | 0.47 | <1.00E-16 | <1.00E-15 | 0.45 | 0.51 |
| *GJA3* | 0.39 | <1.00E-16 | <1.00E-15 | 0.35 | 0.43 |
| *IKZF5* | 0.71 | <1.00E-16 | <1.00E-15 | 0.68 | 0.74 |
| *KCTD15* | 3.3 | <1.00E-16 | <1.00E-15 | 3.67 | 2.89 |
| *MRPL10* | 0.81 | <1.00E-16 | <1.00E-15 | 0.8 | 0.83 |
| *UBE2D4* | 1.31 | <1.00E-16 | <1.00E-15 | 1.28 | 1.36 |
| *DDEF1* | 2.4 | <1.00E-16 | <1.00E-15 | 2.6 | 2.17 |
| *CLIP3* | 0.58 | <1.00E-16 | <1.00E-15 | 0.61 | 0.54 |
| *PMM2* | 1.43 | <1.00E-16 | <1.00E-15 | 1.47 | 1.37 |
| *SIDT1* | 0.56 | <1.00E-16 | <1.00E-15 | 0.53 | 0.6 |
| *TCFL5* | 0.69 | <1.00E-16 | <1.00E-15 | 0.66 | 0.72 |
| *ATP6V1D* | 1.5 | <1.00E-16 | <1.00E-15 | 1.44 | 1.57 |
| *SERPINE1* | 12.65 | <1.00E-16 | <1.00E-15 | 15.82 | 9.61 |
| *DYRK1B* | 0.56 | <1.00E-16 | <1.00E-15 | 0.54 | 0.6 |
| *RBED1* | 0.75 | <1.00E-16 | <1.00E-15 | 0.74 | 0.78 |
| *HEXIM2* | 0.61 | <1.00E-16 | <1.00E-15 | 0.59 | 0.64 |
| *CCL23* | 16.41 | <1.00E-16 | <1.00E-15 | 19.04 | 13.48 |
| *PPP1R14B* | 1.82 | <1.00E-16 | <1.00E-15 | 1.92 | 1.71 |
| *AGER* | 0.53 | <1.00E-16 | <1.00E-15 | 0.56 | 0.49 |
| *ST6GAL1* | 0.58 | <1.00E-16 | <1.00E-15 | 0.55 | 0.62 |
| *MARCH8* | 0.64 | <1.00E-16 | <1.00E-15 | 0.61 | 0.67 |
| *C2orf7* | 2 | <1.00E-16 | <1.00E-15 | 2.12 | 1.87 |
| *MGAT3* | 0.51 | <1.00E-16 | <1.00E-15 | 0.48 | 0.55 |
| *IFNGR2* | 1.76 | <1.00E-16 | <1.00E-15 | 1.67 | 1.89 |
| *CKAP4* | 2.45 | <1.00E-16 | <1.00E-15 | 2.63 | 2.25 |
| *TCTN2* | 0.73 | <1.00E-16 | <1.00E-15 | 0.71 | 0.75 |
| *ARPC3* | 1.33 | <1.00E-16 | <1.00E-15 | 1.3 | 1.37 |
| *EEPD1* | 0.41 | <1.00E-16 | <1.00E-15 | 0.38 | 0.46 |
| *SLC25A38* | 0.66 | <1.00E-16 | <1.00E-15 | 0.64 | 0.69 |
| *TMEM154* | 0.47 | <1.00E-16 | <1.00E-15 | 0.44 | 0.51 |
| *RBPJ* | 2.02 | <1.00E-16 | <1.00E-15 | 2.13 | 1.89 |
| *KIF20A* | 0.55 | <1.00E-16 | <1.00E-15 | 0.52 | 0.58 |
| *ZNF500* | 0.67 | <1.00E-16 | <1.00E-15 | 0.65 | 0.7 |
| *ACSS1* | 0.67 | <1.00E-16 | <1.00E-15 | 0.64 | 0.7 |
| *PRR5* | 0.56 | <1.00E-16 | <1.00E-15 | 0.53 | 0.59 |
| *CD109* | 2.12 | <1.00E-16 | <1.00E-15 | 2.24 | 1.96 |
| *SKAP1* | 0.48 | <1.00E-16 | <1.00E-15 | 0.45 | 0.52 |
| *C14orf43* | 0.63 | <1.00E-16 | <1.00E-15 | 0.6 | 0.66 |
| *ZADH2* | 0.68 | <1.00E-16 | <1.00E-15 | 0.66 | 0.7 |
| *AOAH* | 0.45 | <1.00E-16 | <1.00E-15 | 0.42 | 0.49 |
| *IKIP* | 1.63 | <1.00E-16 | <1.00E-15 | 1.58 | 1.71 |
| *DLD* | 1.6 | <1.00E-16 | <1.00E-15 | 1.67 | 1.52 |
| *LASS4* | 0.56 | <1.00E-16 | <1.00E-15 | 0.53 | 0.59 |
| *RAD52* | 0.65 | <1.00E-16 | <1.00E-15 | 0.63 | 0.68 |
| *LOC51149* | 0.54 | <1.00E-16 | <1.00E-15 | 0.51 | 0.58 |
| *DYRK1A* | 0.75 | <1.00E-16 | <1.00E-15 | 0.73 | 0.77 |
| *SEMA4D* | 0.58 | <1.00E-16 | <1.00E-15 | 0.56 | 0.62 |
| *IL1RAP* | 2.45 | <1.00E-16 | <1.00E-15 | 2.59 | 2.28 |
| *ARMET* | 1.93 | <1.00E-16 | <1.00E-15 | 2.03 | 1.8 |
| *LCP1* | 1.49 | <1.00E-16 | <1.00E-15 | 1.55 | 1.43 |
| *ZNF549* | 0.62 | <1.00E-16 | <1.00E-15 | 0.59 | 0.65 |
| *FLJ45910* | 0.53 | <1.00E-16 | <1.00E-15 | 0.5 | 0.56 |
| *CD200R1* | 2.29 | <1.00E-16 | <1.00E-15 | 2.12 | 2.51 |
| *TREM1* | 4.69 | <1.00E-16 | <1.00E-15 | 5.22 | 4.07 |
| *LACTB* | 1.75 | <1.00E-16 | <1.00E-15 | 1.67 | 1.86 |
| *OSBPL10* | 0.53 | <1.00E-16 | <1.00E-15 | 0.51 | 0.56 |
| *ENTPD7* | 1.59 | <1.00E-16 | <1.00E-15 | 1.65 | 1.52 |
| *ATP5J* | 1.38 | <1.00E-16 | <1.00E-15 | 1.41 | 1.34 |
| *ADAMTS1* | 0.28 | <1.00E-16 | <1.00E-15 | 0.31 | 0.25 |
| *CCDC88A* | 2.57 | <1.00E-16 | <1.00E-15 | 2.75 | 2.37 |
| *PTGDR* | 0.42 | <1.00E-16 | <1.00E-15 | 0.45 | 0.39 |
| *AP1S2* | 1.57 | <1.00E-16 | <1.00E-15 | 1.51 | 1.64 |
| *TIGD7* | 0.6 | <1.00E-16 | <1.00E-15 | 0.62 | 0.58 |
| *C9orf19* | 2.6 | <1.00E-16 | <1.00E-15 | 2.82 | 2.35 |
| *HLA-DOB* | 0.67 | <1.00E-16 | <1.00E-15 | 0.65 | 0.7 |
| *PPARD* | 1.89 | <1.00E-16 | <1.00E-15 | 1.99 | 1.76 |
| *MCC* | 0.53 | <1.00E-16 | <1.00E-15 | 0.55 | 0.5 |
| *ZNF773* | 0.65 | <1.00E-16 | <1.00E-15 | 0.63 | 0.68 |
| *CD22* | 0.66 | <1.00E-16 | <1.00E-15 | 0.64 | 0.69 |
| *C14orf109* | 1.37 | <1.00E-16 | <1.00E-15 | 1.33 | 1.41 |
| *FLI1* | 0.69 | <1.00E-16 | <1.00E-15 | 0.67 | 0.72 |
| *SCFD2* | 1.5 | <1.00E-16 | <1.00E-15 | 1.54 | 1.44 |
| *ZNF479* | 0.62 | <1.00E-16 | <1.00E-15 | 0.64 | 0.59 |
| *YWHAE* | 1.44 | <1.00E-16 | <1.00E-15 | 1.39 | 1.49 |
| *CHEK2* | 1.36 | <1.00E-16 | <1.00E-15 | 1.33 | 1.4 |
| *CD19* | 0.61 | <1.00E-16 | <1.00E-15 | 0.59 | 0.64 |
| *ZNF395* | 0.55 | <1.00E-16 | <1.00E-15 | 0.52 | 0.58 |
| *ZNF740* | 0.73 | <1.00E-16 | <1.00E-15 | 0.71 | 0.75 |
| *C12orf42* | 0.47 | <1.00E-16 | <1.00E-15 | 0.5 | 0.43 |
| *GLIPR1* | 1.52 | <1.00E-16 | <1.00E-15 | 1.58 | 1.46 |
| *SNX29* | 0.66 | <1.00E-16 | <1.00E-15 | 0.64 | 0.69 |
| *LRRC56* | 0.66 | <1.00E-16 | <1.00E-15 | 0.64 | 0.69 |
| *FUCA2* | 1.93 | <1.00E-16 | <1.00E-15 | 1.83 | 2.06 |
| *NKG7* | 0.43 | <1.00E-16 | <1.00E-15 | 0.41 | 0.47 |
| *KIAA0748* | 0.55 | <1.00E-16 | <1.00E-15 | 0.53 | 0.58 |
| *WASF1* | 0.61 | <1.00E-16 | <1.00E-15 | 0.63 | 0.58 |
| *GNS* | 1.85 | <1.00E-16 | <1.00E-15 | 1.76 | 1.96 |
| *TIMP4* | 6.6 | <1.00E-16 | <1.00E-15 | 7.6 | 5.53 |
| *GALNT6* | 1.93 | <1.00E-16 | <1.00E-15 | 1.83 | 2.05 |
| *EHMT2* | 0.7 | <1.00E-16 | <1.00E-15 | 0.68 | 0.72 |
| *TUT1* | 0.73 | <1.00E-16 | <1.00E-15 | 0.75 | 0.71 |
| *MYLIP* | 0.52 | <1.00E-16 | <1.00E-15 | 0.5 | 0.56 |
| *ZFYVE27* | 0.76 | <1.00E-16 | <1.00E-15 | 0.74 | 0.78 |
| *VDAC1* | 1.96 | <1.00E-16 | <1.00E-15 | 2.06 | 1.85 |
| *CLK3* | 0.73 | <1.00E-16 | <1.00E-15 | 0.71 | 0.75 |
| *LDOC1* | 0.52 | <1.00E-16 | <1.00E-15 | 0.49 | 0.55 |
| *ZNF830* | 0.76 | <1.00E-16 | <1.00E-15 | 0.74 | 0.78 |
| *ZHX2* | 0.61 | <1.00E-16 | <1.00E-15 | 0.59 | 0.64 |
| *NME1* | 2.36 | <1.00E-16 | <1.00E-15 | 2.5 | 2.19 |
| *MRPL12* | 1.76 | <1.00E-16 | <1.00E-15 | 1.83 | 1.67 |
| *GPR120* | 9.12 | <1.00E-16 | <1.00E-15 | 10.8 | 7.43 |
| *PTX3* | 2.55 | <1.00E-16 | <1.00E-15 | 2.73 | 2.35 |
| *CTDSP2* | 0.69 | <1.00E-16 | <1.00E-15 | 0.67 | 0.72 |
| *NDUFB3* | 1.64 | <1.00E-16 | <1.00E-15 | 1.68 | 1.58 |
| *SYTL3* | 0.42 | <1.00E-16 | <1.00E-15 | 0.4 | 0.45 |
| *C6orf211* | 2.02 | <1.00E-16 | <1.00E-15 | 2.12 | 1.9 |
| *AUTS2* | 0.54 | <1.00E-16 | <1.00E-15 | 0.57 | 0.51 |
| *HYOU1* | 1.75 | <1.00E-16 | <1.00E-15 | 1.82 | 1.66 |
| *ITGAV* | 1.84 | <1.00E-16 | <1.00E-15 | 1.77 | 1.95 |
| *VILL* | 0.51 | <1.00E-16 | <1.00E-15 | 0.49 | 0.55 |
| *NAT6* | 0.67 | <1.00E-16 | <1.00E-15 | 0.65 | 0.69 |
| *DNAJC3* | 1.99 | <1.00E-16 | <1.00E-15 | 2.07 | 1.88 |
| *STRAP* | 1.27 | <1.00E-16 | <1.00E-15 | 1.29 | 1.24 |
| *ATP2A2* | 1.75 | <1.00E-16 | <1.00E-15 | 1.83 | 1.66 |
| *ZNF224* | 0.65 | <1.00E-16 | <1.00E-15 | 0.63 | 0.68 |
| *CYP51A1* | 2 | <1.00E-16 | <1.00E-15 | 2.1 | 1.88 |
| *NCALD* | 0.41 | <1.00E-16 | <1.00E-15 | 0.38 | 0.44 |
| *KIAA1754* | 1.53 | <1.00E-16 | <1.00E-15 | 1.58 | 1.47 |
| *BTD* | 0.64 | <1.00E-16 | <1.00E-15 | 0.62 | 0.67 |
| *NOMO1* | 1.53 | <1.00E-16 | <1.00E-15 | 1.58 | 1.47 |
| *KLRC1* | 0.45 | <1.00E-16 | <1.00E-15 | 0.43 | 0.49 |
| *SMARCA2* | 0.72 | <1.00E-16 | <1.00E-15 | 0.74 | 0.7 |
| *CPEB4* | 1.71 | <1.00E-16 | <1.00E-15 | 1.78 | 1.64 |
| *LTA4H* | 1.72 | <1.00E-16 | <1.00E-15 | 1.79 | 1.64 |
| *NETO2* | 3.14 | <1.00E-16 | <1.00E-15 | 3.39 | 2.86 |
| *HNMT* | 2.19 | <1.00E-16 | <1.00E-15 | 2.07 | 2.34 |
| *GIGYF1* | 0.65 | <1.00E-16 | <1.00E-15 | 0.63 | 0.68 |
| *CETN2* | 1.45 | <1.00E-16 | <1.00E-15 | 1.49 | 1.4 |
| *GATA6* | 0.25 | <1.00E-16 | <1.00E-15 | 0.23 | 0.28 |
| *ZNF251* | 0.54 | <1.00E-16 | <1.00E-15 | 0.51 | 0.57 |
| *VIPR2* | 0.53 | <1.00E-16 | <1.00E-15 | 0.55 | 0.51 |
| *YPEL1* | 0.47 | <1.00E-16 | <1.00E-15 | 0.45 | 0.51 |
| *FAR1* | 1.71 | <1.00E-16 | <1.00E-15 | 1.77 | 1.62 |
| *RPE* | 1.23 | <1.00E-16 | <1.00E-15 | 1.25 | 1.21 |
| *MAP3K9* | 0.52 | <1.00E-16 | <1.00E-15 | 0.55 | 0.49 |
| *FAM20A* | 5.19 | <1.00E-16 | <1.00E-15 | 5.84 | 4.49 |
| *C11orf66* | 0.5 | <1.00E-16 | <1.00E-15 | 0.48 | 0.54 |
| *SLC2A3* | 1.48 | <1.00E-16 | <1.00E-15 | 1.52 | 1.43 |
| *ZNF671* | 0.63 | <1.00E-16 | <1.00E-15 | 0.62 | 0.66 |
| *HMGCR* | 1.44 | <1.00E-16 | <1.00E-15 | 1.48 | 1.4 |
| *SPINT1* | 4.49 | <1.00E-16 | <1.00E-15 | 5.01 | 3.92 |
| *SLC24A1* | 0.73 | <1.00E-16 | <1.00E-15 | 0.75 | 0.71 |
| *YWHAG* | 2.09 | <1.00E-16 | <1.00E-15 | 2.2 | 1.96 |
| *SQLE* | 1.47 | <1.00E-16 | <1.00E-15 | 1.43 | 1.52 |
| *CTSK* | 0.32 | <1.00E-16 | <1.00E-15 | 0.3 | 0.36 |
| *PHCA* | 2.55 | <1.00E-16 | <1.00E-15 | 2.69 | 2.38 |
| *CYBB* | 3.09 | <1.00E-16 | <1.00E-15 | 3.36 | 2.78 |
| *C3orf21* | 1.86 | <1.00E-16 | <1.00E-15 | 1.93 | 1.78 |
| *C18orf55* | 1.45 | <1.00E-16 | <1.00E-15 | 1.48 | 1.4 |
| *SETD3* | 1.71 | <1.00E-16 | <1.00E-15 | 1.76 | 1.64 |
| *PDSS1* | 2.62 | <1.00E-16 | <1.00E-15 | 2.81 | 2.41 |
| *CST7* | 0.47 | <1.00E-16 | <1.00E-15 | 0.45 | 0.51 |
| *PHF21A* | 0.72 | <1.00E-16 | <1.00E-15 | 0.7 | 0.74 |
| *TSPYL1* | 0.64 | <1.00E-16 | <1.00E-15 | 0.63 | 0.67 |
| *FAM129C* | 0.51 | <1.00E-16 | <1.00E-15 | 0.49 | 0.54 |
| *UPP1* | 2.63 | <1.00E-16 | <1.00E-15 | 2.8 | 2.44 |
| *HEATR3* | 1.41 | <1.00E-16 | <1.00E-15 | 1.43 | 1.37 |
| *HKDC1* | 0.5 | <1.00E-16 | <1.00E-15 | 0.48 | 0.53 |
| *SPEG* | 0.5 | <1.00E-16 | <1.00E-15 | 0.52 | 0.48 |
| *ING1* | 0.64 | <1.00E-16 | <1.00E-15 | 0.62 | 0.67 |
| *TBX21* | 0.5 | <1.00E-16 | <1.00E-15 | 0.47 | 0.53 |
| *FAM8A1* | 0.61 | <1.00E-16 | <1.00E-15 | 0.59 | 0.64 |
| *ATG3* | 1.56 | <1.00E-16 | <1.00E-15 | 1.52 | 1.62 |
| *TIGD3* | 0.49 | <1.00E-16 | <1.00E-15 | 0.47 | 0.52 |
| *ALS2CR13* | 0.51 | <1.00E-16 | <1.00E-15 | 0.48 | 0.54 |
| *CAMK2G* | 0.68 | <1.00E-16 | <1.00E-15 | 0.66 | 0.71 |
| *FAM65B* | 0.48 | <1.00E-16 | <1.00E-15 | 0.46 | 0.51 |
| *MRPS16* | 1.24 | <1.00E-16 | <1.00E-15 | 1.23 | 1.26 |
| *RHOH* | 0.53 | <1.00E-16 | <1.00E-15 | 0.51 | 0.56 |
| *ZBTB4* | 0.58 | <1.00E-16 | <1.00E-15 | 0.56 | 0.6 |
| *DDIT4* | 0.5 | <1.00E-16 | <1.00E-15 | 0.48 | 0.53 |
| *NUS1* | 1.61 | <1.00E-16 | <1.00E-15 | 1.65 | 1.55 |
| *OSBP2* | 0.57 | <1.00E-16 | <1.00E-15 | 0.56 | 0.6 |
| *LRRN1* | 0.36 | <1.00E-16 | <1.00E-15 | 0.38 | 0.34 |
| *RRAS2* | 0.5 | <1.00E-16 | <1.00E-15 | 0.48 | 0.53 |
| *ZMYND10* | 0.49 | <1.00E-16 | <1.00E-15 | 0.51 | 0.46 |
| *HSPA5* | 2.15 | <1.00E-16 | <1.00E-15 | 2.26 | 2.03 |
| *FOXM1* | 0.56 | <1.00E-16 | <1.00E-15 | 0.54 | 0.58 |
| *ATP5H* | 1.39 | <1.00E-16 | <1.00E-15 | 1.42 | 1.36 |
| *LST1* | 3.24 | <1.00E-16 | <1.00E-15 | 3.5 | 2.95 |
| *BBS1* | 0.64 | <1.00E-16 | <1.00E-15 | 0.62 | 0.66 |
| *SUCLA2* | 1.48 | <1.00E-16 | <1.00E-15 | 1.52 | 1.44 |
| *CBLB* | 0.65 | <1.00E-16 | <1.00E-15 | 0.63 | 0.67 |
| *LYNX1* | 3.15 | <1.00E-16 | <1.00E-15 | 3.4 | 2.87 |
| *TBC1D25* | 0.7 | <1.00E-16 | <1.00E-15 | 0.69 | 0.72 |
| *NCOA2* | 0.7 | <1.00E-16 | <1.00E-15 | 0.69 | 0.72 |
| *KALRN* | 0.52 | <1.00E-16 | <1.00E-15 | 0.54 | 0.49 |
| *NGFRAP1* | 0.54 | <1.00E-16 | <1.00E-15 | 0.52 | 0.57 |
| *COX19* | 0.67 | <1.00E-16 | <1.00E-15 | 0.66 | 0.69 |
| *P2RY13* | 3.47 | <1.00E-16 | <1.00E-15 | 3.74 | 3.16 |
| *HNRNPAB* | 1.35 | <1.00E-16 | <1.00E-15 | 1.37 | 1.32 |
| *PCOLCE2* | 17.28 | <1.00E-16 | <1.00E-15 | 20.29 | 13.92 |
| *AFF3* | 0.49 | <1.00E-16 | <1.00E-15 | 0.47 | 0.52 |
| *TET1* | 0.51 | <1.00E-16 | <1.00E-15 | 0.49 | 0.54 |
| *TLR2* | 2.27 | <1.00E-16 | <1.00E-15 | 2.16 | 2.42 |
| *FN1* | 10.18 | <1.00E-16 | <1.00E-15 | 9.15 | 11.62 |
| *CDGAP* | 3.35 | <1.00E-16 | <1.00E-15 | 3.62 | 3.05 |
| *GZMA* | 0.43 | <1.00E-16 | <1.00E-15 | 0.41 | 0.45 |
| *UTRN* | 0.58 | <1.00E-16 | <1.00E-15 | 0.56 | 0.6 |
| *VMAC* | 0.55 | <1.00E-16 | <1.00E-15 | 0.53 | 0.57 |
| *ZNF528* | 0.59 | <1.00E-16 | <1.00E-15 | 0.61 | 0.56 |
| *SEMA3C* | 3.11 | <1.00E-16 | <1.00E-15 | 2.9 | 3.39 |
| *ZNF346* | 0.65 | <1.00E-16 | <1.00E-15 | 0.63 | 0.67 |
| *TBC1D17* | 0.59 | <1.00E-16 | <1.00E-15 | 0.57 | 0.62 |
| *LOC388969* | 0.74 | <1.00E-16 | <1.00E-15 | 0.72 | 0.76 |
| *NUDT9* | 1.4 | <1.00E-16 | <1.00E-15 | 1.38 | 1.43 |
| *ZNF74* | 0.71 | <1.00E-16 | <1.00E-15 | 0.7 | 0.73 |
| *LIG1* | 0.62 | <1.00E-16 | <1.00E-15 | 0.6 | 0.64 |
| *FGD4* | 3.2 | <1.00E-16 | <1.00E-15 | 3.44 | 2.93 |
| *CLEC4A* | 2.42 | <1.00E-16 | <1.00E-15 | 2.31 | 2.56 |
| *CCDC28A* | 0.67 | <1.00E-16 | <1.00E-15 | 0.66 | 0.69 |
| *SATB1* | 0.53 | <1.00E-16 | <1.00E-15 | 0.51 | 0.55 |
| *HOXB2* | 0.58 | <1.00E-16 | <1.00E-15 | 0.6 | 0.56 |
| *WDR59* | 0.72 | <1.00E-16 | <1.00E-15 | 0.7 | 0.74 |
| *ZNF554* | 0.55 | <1.00E-16 | <1.00E-15 | 0.53 | 0.57 |
| *TIAM2* | 0.6 | <1.00E-16 | <1.00E-15 | 0.62 | 0.58 |
| *ZNF419* | 0.62 | <1.00E-16 | <1.00E-15 | 0.6 | 0.64 |
| *SLC39A8* | 4.34 | <1.00E-16 | <1.00E-15 | 4.65 | 3.98 |
| *PSCD1* | 0.58 | <1.00E-16 | <1.00E-15 | 0.56 | 0.6 |
| *INPP5E* | 0.74 | <1.00E-16 | <1.00E-15 | 0.73 | 0.76 |
| *CRK* | 1.33 | <1.00E-16 | <1.00E-15 | 1.31 | 1.36 |
| *VSTM3* | 0.48 | <1.00E-16 | <1.00E-15 | 0.46 | 0.51 |
| *LARS2* | 1.31 | <1.00E-16 | <1.00E-15 | 1.33 | 1.29 |
| *EPHX2* | 0.47 | <1.00E-16 | <1.00E-15 | 0.45 | 0.5 |
| *TSHZ1* | 0.63 | <1.00E-16 | <1.00E-15 | 0.61 | 0.65 |
| *PLCB1* | 0.59 | <1.00E-16 | <1.00E-15 | 0.61 | 0.57 |
| *AGPS* | 1.46 | <1.00E-16 | <1.00E-15 | 1.49 | 1.42 |
| *IDH3A* | 1.66 | <1.00E-16 | <1.00E-15 | 1.71 | 1.6 |
| *FOXJ2* | 0.77 | <1.00E-16 | <1.00E-15 | 0.76 | 0.78 |
| *FILIP1L* | 3.87 | <1.00E-16 | <1.00E-15 | 4.14 | 3.56 |
| *KRBA1* | 0.63 | <1.00E-16 | <1.00E-15 | 0.61 | 0.65 |
| *TRIM39* | 0.75 | <1.00E-16 | <1.00E-15 | 0.74 | 0.77 |
| *AKAP8* | 0.84 | <1.00E-16 | <1.00E-15 | 0.83 | 0.85 |
| *BCAT1* | 1.79 | <1.00E-16 | <1.00E-15 | 1.85 | 1.72 |
| *ZIK1* | 0.6 | <1.00E-16 | <1.00E-15 | 0.58 | 0.62 |
| *IL32* | 0.69 | <1.00E-16 | <1.00E-15 | 0.67 | 0.71 |
| *ZNF509* | 0.67 | <1.00E-16 | <1.00E-15 | 0.65 | 0.68 |
| *C17orf57* | 0.6 | <1.00E-16 | <1.00E-15 | 0.62 | 0.58 |
| *MCM7* | 0.56 | <1.00E-16 | <1.00E-15 | 0.54 | 0.58 |
| *IL18* | 3.09 | <1.00E-16 | <1.00E-15 | 3.27 | 2.87 |
| *ADAMTS13* | 0.51 | <1.00E-16 | <1.00E-15 | 0.49 | 0.54 |
| *SEC23B* | 1.41 | <1.00E-16 | <1.00E-15 | 1.43 | 1.37 |
| *KIAA0152* | 1.4 | <1.00E-16 | <1.00E-15 | 1.42 | 1.36 |
| *PRKX* | 0.8 | <1.00E-16 | <1.00E-15 | 0.79 | 0.81 |
| *C17orf56* | 0.65 | <1.00E-16 | <1.00E-15 | 0.63 | 0.67 |
| *NARS* | 1.4 | <1.00E-16 | <1.00E-15 | 1.42 | 1.37 |
| *DPAGT1* | 1.5 | <1.00E-16 | <1.00E-15 | 1.47 | 1.53 |
| *KIAA1267* | 0.69 | <1.00E-16 | <1.00E-15 | 0.68 | 0.71 |
| *AADACL1* | 2.81 | <1.00E-16 | <1.00E-15 | 2.96 | 2.64 |
| *ATG16L1* | 0.71 | <1.00E-16 | <1.00E-15 | 0.7 | 0.73 |
| *TLE2* | 0.54 | <1.00E-16 | <1.00E-15 | 0.55 | 0.51 |
| *RBM4* | 0.79 | <1.00E-16 | <1.00E-15 | 0.78 | 0.8 |
| *ZNF333* | 0.68 | <1.00E-16 | <1.00E-15 | 0.67 | 0.7 |
| *KIAA0922* | 0.6 | <1.00E-16 | <1.00E-15 | 0.58 | 0.62 |
| *LYVE1* | 12.75 | <1.00E-16 | <1.00E-15 | 13.77 | 10.69 |
| *STIP1* | 1.61 | <1.00E-16 | <1.00E-15 | 1.65 | 1.56 |
| *MNDA* | 3.28 | <1.00E-16 | <1.00E-15 | 3.49 | 3.03 |
| *TGFBI* | 3.65 | <1.00E-16 | <1.00E-15 | 3.42 | 3.96 |
| *TCF20* | 0.76 | <1.00E-16 | <1.00E-15 | 0.74 | 0.77 |
| *SLC4A10* | 0.43 | <1.00E-16 | <1.00E-15 | 0.45 | 0.41 |
| *TPM4* | 2.19 | <1.00E-16 | <1.00E-15 | 2.28 | 2.09 |
| *ANKRD57* | 2.3 | <1.00E-16 | <1.00E-15 | 2.21 | 2.42 |
| *NAP1L2* | 0.4 | <1.00E-16 | <1.00E-15 | 0.38 | 0.42 |
| *LTK* | 0.51 | <1.00E-16 | <1.00E-15 | 0.49 | 0.53 |
| *FGD3* | 0.51 | <1.00E-16 | <1.00E-15 | 0.49 | 0.53 |
| *ZNF763* | 0.6 | <1.00E-16 | <1.00E-15 | 0.58 | 0.62 |
| *TPK1* | 1.38 | <1.00E-16 | <1.00E-15 | 1.36 | 1.41 |
| *PSMB5* | 1.55 | <1.00E-16 | <1.00E-15 | 1.59 | 1.51 |
| *MRPS28* | 1.56 | <1.00E-16 | <1.00E-15 | 1.59 | 1.52 |
| *HSD17B12* | 1.68 | <1.00E-16 | <1.00E-15 | 1.71 | 1.64 |
| *EIF2S1* | 1.4 | <1.00E-16 | <1.00E-15 | 1.42 | 1.37 |
| *AKNA* | 0.52 | <1.00E-16 | <1.00E-15 | 0.51 | 0.55 |
| *TACC3* | 0.73 | <1.00E-16 | <1.00E-15 | 0.72 | 0.74 |
| *COPB2* | 1.3 | <1.00E-16 | <1.00E-15 | 1.32 | 1.28 |
| *BOLA3* | 1.88 | <1.00E-16 | <1.00E-15 | 1.94 | 1.81 |
| *NMT2* | 0.54 | <1.00E-16 | <1.00E-15 | 0.53 | 0.56 |
| *PSMB7* | 1.46 | <1.00E-16 | <1.00E-15 | 1.44 | 1.5 |
| *NDUFB6* | 1.38 | <1.00E-16 | <1.00E-15 | 1.4 | 1.35 |
| *PDPN* | 6.32 | <1.00E-16 | <1.00E-15 | 5.85 | 6.97 |
| *KSR2* | 0.45 | <1.00E-16 | <1.00E-15 | 0.46 | 0.42 |
| *CCNA2* | 0.54 | <1.00E-16 | <1.00E-15 | 0.53 | 0.56 |
| *PRKAR2B* | 3.03 | <1.00E-16 | <1.00E-15 | 2.9 | 3.21 |
| *TIMM50* | 1.31 | <1.00E-16 | <1.00E-15 | 1.33 | 1.29 |
| *TCL6* | 0.54 | <1.00E-16 | <1.00E-15 | 0.53 | 0.56 |
| *PABPC1L* | 0.57 | <1.00E-16 | <1.00E-15 | 0.59 | 0.56 |
| *EZH1* | 0.58 | <1.00E-16 | <1.00E-15 | 0.56 | 0.6 |
| *ANXA1* | 1.81 | <1.00E-16 | <1.00E-15 | 1.77 | 1.87 |
| *HKR1* | 0.64 | <1.00E-16 | <1.00E-15 | 0.63 | 0.66 |
| *TMEM33* | 1.48 | <1.00E-16 | <1.00E-15 | 1.5 | 1.44 |
| *REEP5* | 1.46 | <1.00E-16 | <1.00E-15 | 1.43 | 1.49 |
| *PEX6* | 0.7 | <1.00E-16 | <1.00E-15 | 0.71 | 0.68 |
| *BMP4* | 0.29 | <1.00E-16 | <1.00E-15 | 0.31 | 0.27 |
| *KLHL14* | 0.39 | <1.00E-16 | <1.00E-15 | 0.38 | 0.41 |
| *PAPSS1* | 2.43 | <1.00E-16 | <1.00E-15 | 2.53 | 2.3 |
| *IL2RB* | 0.63 | <1.00E-16 | <1.00E-15 | 0.65 | 0.62 |
| *ZNF585B* | 0.64 | <1.00E-16 | <1.00E-15 | 0.65 | 0.63 |
| *PDIA3* | 1.73 | <1.00E-16 | <1.00E-15 | 1.69 | 1.78 |
| *LDHA* | 2 | <1.00E-16 | <1.00E-15 | 2.06 | 1.93 |
| *GUCA1B* | 0.53 | <1.00E-16 | <1.00E-15 | 0.54 | 0.51 |
| *KBTBD7* | 0.67 | <1.00E-16 | <1.00E-15 | 0.66 | 0.68 |
| *C14orf93* | 0.68 | <1.00E-16 | <1.00E-15 | 0.67 | 0.69 |
| *CHCHD3* | 1.53 | <1.00E-16 | <1.00E-15 | 1.5 | 1.56 |
| *COX7A2* | 1.62 | <1.00E-16 | <1.00E-15 | 1.65 | 1.58 |
| *GPI* | 1.54 | <1.00E-16 | <1.00E-15 | 1.57 | 1.5 |
| *TMTC1* | 4.21 | <1.00E-16 | <1.00E-15 | 4.07 | 4.41 |
| *CCT5* | 1.6 | <1.00E-16 | <1.00E-15 | 1.63 | 1.55 |
| *PAPSS2* | 1.91 | <1.00E-16 | <1.00E-15 | 1.96 | 1.85 |
| *TPMT* | 2.14 | <1.00E-16 | <1.00E-15 | 2.2 | 2.05 |
| *FKBP4* | 1.64 | <1.00E-16 | <1.00E-15 | 1.68 | 1.6 |
| *PSMD1* | 1.5 | <1.00E-16 | <1.00E-15 | 1.53 | 1.47 |
| *ALG8* | 1.59 | <1.00E-16 | <1.00E-15 | 1.62 | 1.56 |
| *GALNT1* | 1.42 | <1.00E-16 | <1.00E-15 | 1.44 | 1.39 |
| *BRD3* | 0.67 | <1.00E-16 | <1.00E-15 | 0.66 | 0.69 |
| *PDGFD* | 0.49 | <1.00E-16 | <1.00E-15 | 0.48 | 0.51 |
| *HMGB2* | 0.53 | <1.00E-16 | <1.00E-15 | 0.51 | 0.54 |
| *HSP90B1* | 1.85 | <1.00E-16 | <1.00E-15 | 1.89 | 1.8 |
| *RASSF3* | 0.67 | <1.00E-16 | <1.00E-15 | 0.65 | 0.68 |
| *BCAS4* | 0.56 | <1.00E-16 | <1.00E-15 | 0.55 | 0.58 |
| *PHLDB1* | 3.07 | <1.00E-16 | <1.00E-15 | 3.21 | 2.89 |
| *C12orf59* | 9.54 | <1.00E-16 | <1.00E-15 | 10.39 | 8.55 |
| *CCDC88C* | 0.52 | <1.00E-16 | <1.00E-15 | 0.51 | 0.54 |
| *NRP1* | 4.68 | <1.00E-16 | <1.00E-15 | 5 | 4.3 |
| *SQRDL* | 2.46 | <1.00E-16 | <1.00E-15 | 2.36 | 2.59 |
| *FLJ36208* | 0.57 | <1.00E-16 | <1.00E-15 | 0.58 | 0.55 |
| *NDUFA4* | 1.53 | <1.00E-16 | <1.00E-15 | 1.56 | 1.5 |
| *MLL5* | 0.72 | <1.00E-16 | <1.00E-15 | 0.71 | 0.73 |
| *MAPK13* | 1.67 | <1.00E-16 | <1.00E-15 | 1.7 | 1.62 |
| *LASS6* | 1.37 | <1.00E-16 | <1.00E-15 | 1.39 | 1.35 |
| *MDH1* | 1.68 | <1.00E-16 | <1.00E-15 | 1.72 | 1.64 |
| *PDSS2* | 1.48 | <1.00E-16 | <1.00E-15 | 1.5 | 1.45 |
| *CTNND1* | 1.85 | <1.00E-16 | <1.00E-15 | 1.8 | 1.91 |
| *ZNF594* | 0.56 | <1.00E-16 | <1.00E-15 | 0.57 | 0.54 |
| *SLA2* | 0.57 | <1.00E-16 | <1.00E-15 | 0.56 | 0.58 |
| *BACH2* | 0.52 | <1.00E-16 | <1.00E-15 | 0.51 | 0.54 |
| *BAIAP3* | 0.41 | <1.00E-16 | <1.00E-15 | 0.42 | 0.4 |
| *TXNDC5* | 1.53 | <1.00E-16 | <1.00E-15 | 1.56 | 1.5 |
| *IK* | 0.78 | <1.00E-16 | <1.00E-15 | 0.77 | 0.79 |
| *TOX* | 0.62 | <1.00E-16 | <1.00E-15 | 0.6 | 0.63 |
| *ACPP* | 2.89 | <1.00E-16 | <1.00E-15 | 3.02 | 2.74 |
| *TP53I3* | 2.41 | <1.00E-16 | <1.00E-15 | 2.34 | 2.51 |
| *SLC9A3* | 0.53 | <1.00E-16 | <1.00E-15 | 0.55 | 0.52 |
| *SGK269* | 0.68 | <1.00E-16 | <1.00E-15 | 0.67 | 0.7 |
| *APLP2* | 1.92 | <1.00E-16 | <1.00E-15 | 1.96 | 1.86 |
| *PROCR* | 4.64 | <1.00E-16 | <1.00E-15 | 4.93 | 4.31 |
| *TSPYL4* | 0.65 | <1.00E-16 | <1.00E-15 | 0.66 | 0.63 |
| *RNF212* | 0.5 | <1.00E-16 | <1.00E-15 | 0.49 | 0.52 |
| *USP31* | 1.39 | <1.00E-16 | <1.00E-15 | 1.37 | 1.42 |
| *PSMA6* | 1.69 | <1.00E-16 | <1.00E-15 | 1.65 | 1.74 |
| *SNRK* | 0.6 | <1.00E-16 | <1.00E-15 | 0.58 | 0.61 |
| *TMEM42* | 0.67 | <1.00E-16 | <1.00E-15 | 0.66 | 0.68 |
| *MAGT1* | 1.49 | <1.00E-16 | <1.00E-15 | 1.51 | 1.47 |
| *ACSL4* | 2.06 | <1.00E-16 | <1.00E-15 | 2.11 | 2 |
| *AP1G2* | 0.64 | <1.00E-16 | <1.00E-15 | 0.63 | 0.66 |
| *GTF3C6* | 1.73 | <1.00E-16 | <1.00E-15 | 1.77 | 1.69 |
| *ZNF490* | 0.78 | <1.00E-16 | <1.00E-15 | 0.77 | 0.79 |
| *ZNF425* | 0.55 | <1.00E-16 | <1.00E-15 | 0.54 | 0.57 |
| *SPTLC1* | 1.31 | <1.00E-16 | <1.00E-15 | 1.32 | 1.29 |
| *PDLIM5* | 1.58 | <1.00E-16 | <1.00E-15 | 1.6 | 1.55 |
| *SAFB* | 0.8 | <1.00E-16 | <1.00E-15 | 0.79 | 0.8 |
| *EFEMP2* | 0.61 | <1.00E-16 | <1.00E-15 | 0.62 | 0.59 |
| *COG1* | 0.69 | <1.00E-16 | <1.00E-15 | 0.68 | 0.7 |
| *PET112L* | 1.57 | <1.00E-16 | <1.00E-15 | 1.59 | 1.53 |
| *ARHGEF18* | 0.57 | <1.00E-16 | <1.00E-15 | 0.56 | 0.58 |
| *BANF1* | 1.38 | <1.00E-16 | <1.00E-15 | 1.4 | 1.36 |
| *SLC44A2* | 0.62 | <1.00E-16 | <1.00E-15 | 0.61 | 0.64 |
| *POLE4* | 1.38 | <1.00E-16 | <1.00E-15 | 1.4 | 1.36 |
| *PEG10* | 0.37 | <1.00E-16 | <1.00E-15 | 0.39 | 0.36 |
| *SDHD* | 1.49 | <1.00E-16 | <1.00E-15 | 1.5 | 1.46 |
| *PPIC* | 7.83 | <1.00E-16 | <1.00E-15 | 7.29 | 8.84 |
| *FAM80B* | 0.64 | <1.00E-16 | <1.00E-15 | 0.63 | 0.65 |
| *CEL* | 0.44 | <1.00E-16 | <1.00E-15 | 0.43 | 0.46 |
| *JTV1* | 1.64 | <1.00E-16 | <1.00E-15 | 1.67 | 1.61 |
| *OFD1* | 0.59 | <1.00E-16 | <1.00E-15 | 0.58 | 0.6 |
| *PSMD14* | 2 | <1.00E-16 | <1.00E-15 | 2.06 | 1.94 |
| *ME2* | 1.77 | <1.00E-16 | <1.00E-15 | 1.81 | 1.73 |
| *ZNF154* | 0.52 | <1.00E-16 | <1.00E-15 | 0.51 | 0.53 |
| *TRIM2* | 0.52 | <1.00E-16 | <1.00E-15 | 0.53 | 0.5 |
| *ORM2* | 0.41 | <1.00E-16 | <1.00E-15 | 0.43 | 0.4 |
| *GTSE1* | 0.53 | <1.00E-16 | <1.00E-15 | 0.52 | 0.55 |
| *ATP5J2* | 1.43 | <1.00E-16 | <1.00E-15 | 1.41 | 1.45 |
| *YTHDC1* | 0.67 | <1.00E-16 | <1.00E-15 | 0.66 | 0.68 |
| *PTCH1* | 0.44 | <1.00E-16 | <1.00E-15 | 0.43 | 0.46 |
| *PAIP2B* | 0.52 | <1.00E-16 | <1.00E-15 | 0.51 | 0.54 |
| *ZNF211* | 0.62 | <1.00E-16 | <1.00E-15 | 0.61 | 0.63 |
| *LOC285908* | 0.55 | <1.00E-16 | <1.00E-15 | 0.56 | 0.53 |
| *TAS2R5* | 0.45 | <1.00E-16 | <1.00E-15 | 0.46 | 0.44 |
| *CPAMD8* | 0.35 | <1.00E-16 | <1.00E-15 | 0.36 | 0.34 |
| *TMEM80* | 0.62 | <1.00E-16 | <1.00E-15 | 0.61 | 0.63 |
| *FAM134C* | 0.72 | <1.00E-16 | <1.00E-15 | 0.71 | 0.73 |
| *ARF4* | 1.47 | <1.00E-16 | <1.00E-15 | 1.49 | 1.45 |
| *YIPF6* | 1.41 | <1.00E-16 | <1.00E-15 | 1.39 | 1.43 |
| *CDK5* | 1.91 | <1.00E-16 | <1.00E-15 | 1.95 | 1.86 |
| *BRPF3* | 0.76 | <1.00E-16 | <1.00E-15 | 0.75 | 0.77 |
| *SSFA2* | 1.43 | <1.00E-16 | <1.00E-15 | 1.42 | 1.45 |
| *FNBP4* | 0.65 | <1.00E-16 | <1.00E-15 | 0.64 | 0.66 |
| *GNG7* | 0.53 | <1.00E-16 | <1.00E-15 | 0.52 | 0.54 |
| *ZNF135* | 0.54 | <1.00E-16 | <1.00E-15 | 0.53 | 0.56 |
| *ENPP5* | 0.43 | <1.00E-16 | <1.00E-15 | 0.42 | 0.45 |
| *FZD6* | 0.57 | <1.00E-16 | <1.00E-15 | 0.58 | 0.56 |
| *P4HB* | 1.72 | <1.00E-16 | <1.00E-15 | 1.69 | 1.75 |
| *RYR1* | 2.08 | <1.00E-16 | <1.00E-15 | 2.12 | 2.03 |
| *ZNF577* | 0.65 | <1.00E-16 | <1.00E-15 | 0.64 | 0.66 |
| *FANCF* | 0.75 | <1.00E-16 | <1.00E-15 | 0.76 | 0.74 |
| *ZBTB16* | 0.45 | <1.00E-16 | <1.00E-15 | 0.46 | 0.44 |
| *PICALM* | 1.53 | <1.00E-16 | <1.00E-15 | 1.55 | 1.5 |
| *COX17* | 1.59 | <1.00E-16 | <1.00E-15 | 1.61 | 1.57 |
| *TIMM23* | 1.49 | <1.00E-16 | <1.00E-15 | 1.51 | 1.47 |
| *MMRN1* | 0.39 | <1.00E-16 | <1.00E-15 | 0.4 | 0.38 |
| *ARHGEF9* | 0.58 | <1.00E-16 | <1.00E-15 | 0.59 | 0.57 |
| *PSMC2* | 1.53 | <1.00E-16 | <1.00E-15 | 1.51 | 1.55 |
| *M6PR* | 1.55 | <1.00E-16 | <1.00E-15 | 1.53 | 1.58 |
| *HOPX* | 0.42 | <1.00E-16 | <1.00E-15 | 0.41 | 0.43 |
| *STK10* | 0.66 | <1.00E-16 | <1.00E-15 | 0.67 | 0.65 |
| *SERPINA1* | 6.44 | <1.00E-16 | <1.00E-15 | 6.78 | 6.04 |
| *TMED10* | 1.32 | <1.00E-16 | <1.00E-15 | 1.31 | 1.34 |
| *ASPH* | 3.87 | <1.00E-16 | <1.00E-15 | 4.05 | 3.67 |
| *DGKA* | 0.56 | <1.00E-16 | <1.00E-15 | 0.55 | 0.58 |
| *MPPE1* | 0.65 | <1.00E-16 | <1.00E-15 | 0.64 | 0.66 |
| *MESDC2* | 1.28 | <1.00E-16 | <1.00E-15 | 1.29 | 1.27 |
| *OSBPL11* | 1.4 | <1.00E-16 | <1.00E-15 | 1.39 | 1.42 |
| *OSBPL7* | 0.51 | <1.00E-16 | <1.00E-15 | 0.5 | 0.53 |
| *SNX9* | 1.24 | <1.00E-16 | <1.00E-15 | 1.25 | 1.23 |
| *TRIM4* | 0.7 | <1.00E-16 | <1.00E-15 | 0.69 | 0.71 |
| *UGCGL2* | 2.56 | <1.00E-16 | <1.00E-15 | 2.5 | 2.64 |
| *POLG2* | 0.67 | <1.00E-16 | <1.00E-15 | 0.68 | 0.66 |
| *CD44* | 1.64 | <1.00E-16 | <1.00E-15 | 1.67 | 1.62 |
| *ABCA1* | 0.49 | <1.00E-16 | <1.00E-15 | 0.48 | 0.5 |
| *CALU* | 2.28 | <1.00E-16 | <1.00E-15 | 2.32 | 2.23 |
| *ZNF263* | 0.79 | <1.00E-16 | <1.00E-15 | 0.79 | 0.8 |
| *CREBBP* | 0.71 | <1.00E-16 | <1.00E-15 | 0.7 | 0.72 |
| *PTPN4* | 0.54 | <1.00E-16 | <1.00E-15 | 0.55 | 0.53 |
| *CTNS* | 2.04 | <1.00E-16 | <1.00E-15 | 2 | 2.09 |
| *PBX2* | 0.7 | <1.00E-16 | <1.00E-15 | 0.69 | 0.71 |
| *RTN4* | 1.69 | <1.00E-16 | <1.00E-15 | 1.67 | 1.73 |
| *TNRC6A* | 0.75 | <1.00E-16 | <1.00E-15 | 0.74 | 0.76 |
| *UBE3C* | 1.29 | <1.00E-16 | <1.00E-15 | 1.3 | 1.28 |
| *TNK1* | 0.59 | <1.00E-16 | <1.00E-15 | 0.6 | 0.58 |
| *HOXC4* | 0.54 | <1.00E-16 | <1.00E-15 | 0.53 | 0.55 |
| *MCTP2* | 0.58 | <1.00E-16 | <1.00E-15 | 0.59 | 0.57 |
| *DZIP1L* | 3.17 | <1.00E-16 | <1.00E-15 | 3.07 | 3.3 |
| *NUMA1* | 0.65 | <1.00E-16 | <1.00E-15 | 0.66 | 0.64 |
| *ARMCX2* | 0.6 | <1.00E-16 | <1.00E-15 | 0.59 | 0.61 |
| *FAM167A* | 0.52 | <1.00E-16 | <1.00E-15 | 0.52 | 0.5 |
| *PLXDC2* | 2.78 | <1.00E-16 | <1.00E-15 | 2.86 | 2.69 |
| *HMG20A* | 0.74 | <1.00E-16 | <1.00E-15 | 0.74 | 0.75 |
| *PHF17* | 0.59 | <1.00E-16 | <1.00E-15 | 0.58 | 0.6 |
| *C17orf79* | 1.89 | <1.00E-16 | <1.00E-15 | 1.86 | 1.93 |
| *SKAP2* | 1.71 | <1.00E-16 | <1.00E-15 | 1.74 | 1.68 |
| *C14orf156* | 1.57 | <1.00E-16 | <1.00E-15 | 1.55 | 1.59 |
| *RTN3* | 1.47 | <1.00E-16 | <1.00E-15 | 1.46 | 1.49 |
| *CD244* | 0.4 | <1.00E-16 | <1.00E-15 | 0.39 | 0.41 |
| *VASP* | 1.99 | <1.00E-16 | <1.00E-15 | 2.02 | 1.94 |
| *NEFH* | 2.07 | <1.00E-16 | <1.00E-15 | 2.11 | 2.02 |
| *GAS2L3* | 2.18 | <1.00E-16 | <1.00E-15 | 2.13 | 2.24 |
| *FNDC3B* | 2.47 | <1.00E-16 | <1.00E-15 | 2.41 | 2.54 |
| *PIK3CB* | 1.3 | <1.00E-16 | <1.00E-15 | 1.31 | 1.29 |
| *GATS* | 0.51 | <1.00E-16 | <1.00E-15 | 0.5 | 0.52 |
| *PILRB* | 0.6 | <1.00E-16 | <1.00E-15 | 0.59 | 0.61 |
| *TUSC3* | 0.62 | <1.00E-16 | <1.00E-15 | 0.62 | 0.56 |
| *DNAJC10* | 1.59 | <1.00E-16 | <1.00E-15 | 1.6 | 1.56 |
| *DNASE1* | 0.59 | <1.00E-16 | <1.00E-15 | 0.6 | 0.58 |
| *TFAP4* | 0.65 | <1.00E-16 | <1.00E-15 | 0.65 | 0.66 |
| *GLT25D1* | 1.92 | <1.00E-16 | <1.00E-15 | 1.89 | 1.96 |
| *GNLY* | 0.51 | <1.00E-16 | <1.00E-15 | 0.51 | 0.5 |
| *C19orf44* | 0.7 | <1.00E-16 | <1.00E-15 | 0.71 | 0.7 |
| *LRP12* | 3.57 | <1.00E-16 | <1.00E-15 | 3.68 | 3.43 |
| *GOLIM4* | 2.31 | <1.00E-16 | <1.00E-15 | 2.36 | 2.25 |
| *C4orf18* | 1.92 | <1.00E-16 | <1.00E-15 | 1.95 | 1.88 |
| *ZER1* | 0.64 | <1.00E-16 | <1.00E-15 | 0.64 | 0.65 |
| *KIF5B* | 1.38 | <1.00E-16 | <1.00E-15 | 1.39 | 1.37 |
| *PDIA6* | 1.87 | <1.00E-16 | <1.00E-15 | 1.84 | 1.9 |
| *INPP1* | 1.74 | <1.00E-16 | <1.00E-15 | 1.72 | 1.77 |
| *NPR2* | 0.47 | <1.00E-16 | <1.00E-15 | 0.46 | 0.48 |
| *PCDH9* | 0.52 | <1.00E-16 | <1.00E-15 | 0.53 | 0.51 |
| *FANCD2* | 0.58 | <1.00E-16 | <1.00E-15 | 0.57 | 0.59 |
| *FAM113A* | 0.61 | <1.00E-16 | <1.00E-15 | 0.61 | 0.62 |
| *AS3MT* | 0.45 | <1.00E-16 | <1.00E-15 | 0.44 | 0.46 |
| *C17orf65* | 0.64 | <1.00E-16 | <1.00E-15 | 0.65 | 0.63 |
| *TFDP2* | 0.56 | <1.00E-16 | <1.00E-15 | 0.55 | 0.57 |
| *PDIA4* | 2.16 | <1.00E-16 | <1.00E-15 | 2.2 | 2.12 |
| *SDC4* | 3.24 | <1.00E-16 | <1.00E-15 | 3.16 | 3.36 |
| *CANX* | 1.6 | <1.00E-16 | <1.00E-15 | 1.62 | 1.59 |
| *ARL17P1* | 0.58 | <1.00E-16 | <1.00E-15 | 0.57 | 0.59 |
| *FAM120B* | 0.73 | <1.00E-16 | <1.00E-15 | 0.73 | 0.72 |
| *RAB31* | 2.1 | <1.00E-16 | <1.00E-15 | 2.06 | 2.14 |
| *PPAPDC2* | 0.59 | <1.00E-16 | <1.00E-15 | 0.59 | 0.6 |
| *PCMTD2* | 0.57 | <1.00E-16 | <1.00E-15 | 0.56 | 0.58 |
| *KRT72* | 0.26 | <1.00E-16 | <1.00E-15 | 0.27 | 0.25 |
| *KCTD12* | 1.93 | <1.00E-16 | <1.00E-15 | 1.9 | 1.97 |
| *PPARG* | 3.92 | <1.00E-16 | <1.00E-15 | 3.83 | 4.03 |
| *PLAU* | 3.52 | <1.00E-16 | <1.00E-15 | 3.43 | 3.64 |
| *CBFA2T2* | 0.69 | <1.00E-16 | <1.00E-15 | 0.69 | 0.7 |
| *TRIB1* | 2.46 | <1.00E-16 | <1.00E-15 | 2.5 | 2.41 |
| *ZNF839* | 0.7 | <1.00E-16 | <1.00E-15 | 0.69 | 0.7 |
| *PXK* | 1.27 | <1.00E-16 | <1.00E-15 | 1.26 | 1.27 |
| *NCR1* | 0.46 | <1.00E-16 | <1.00E-15 | 0.47 | 0.46 |
| *NDUFAB1* | 1.59 | <1.00E-16 | <1.00E-15 | 1.61 | 1.58 |
| *HIP1R* | 0.62 | <1.00E-16 | <1.00E-15 | 0.61 | 0.62 |
| *MAP3K1* | 0.6 | <1.00E-16 | <1.00E-15 | 0.59 | 0.61 |
| *NLRP1* | 0.51 | <1.00E-16 | <1.00E-15 | 0.51 | 0.5 |
| *NDC80* | 0.54 | <1.00E-16 | <1.00E-15 | 0.53 | 0.55 |
| *DGKD* | 0.52 | <1.00E-16 | <1.00E-15 | 0.51 | 0.53 |
| *OSTM1* | 1.53 | <1.00E-16 | <1.00E-15 | 1.52 | 1.55 |
| *PREP* | 1.35 | <1.00E-16 | <1.00E-15 | 1.36 | 1.34 |
| *TM9SF2* | 1.42 | <1.00E-16 | <1.00E-15 | 1.41 | 1.43 |
| *CENPK* | 0.44 | <1.00E-16 | <1.00E-15 | 0.44 | 0.45 |
| *NLRC4* | 2.44 | <1.00E-16 | <1.00E-15 | 2.41 | 2.49 |
| *MSRA* | 1.77 | <1.00E-16 | <1.00E-15 | 1.75 | 1.79 |
| *SPG21* | 1.58 | <1.00E-16 | <1.00E-15 | 1.57 | 1.6 |
| *IL18RAP* | 0.48 | <1.00E-16 | <1.00E-15 | 0.49 | 0.47 |
| *CIT* | 0.56 | <1.00E-16 | <1.00E-15 | 0.55 | 0.56 |
| *PDE6G* | 0.44 | <1.00E-16 | <1.00E-15 | 0.45 | 0.43 |
| *LIMS1* | 2.05 | <1.00E-16 | <1.00E-15 | 2.08 | 2.01 |
| *C10orf33* | 0.48 | <1.00E-16 | <1.00E-15 | 0.48 | 0.47 |
| *ZSCAN18* | 0.52 | <1.00E-16 | <1.00E-15 | 0.52 | 0.51 |
| *AMOT* | 0.49 | <1.00E-16 | <1.00E-15 | 0.5 | 0.48 |
| *C3orf19* | 0.71 | <1.00E-16 | <1.00E-15 | 0.72 | 0.71 |
| *PLEKHH2* | 0.32 | <1.00E-16 | <1.00E-15 | 0.33 | 0.31 |
| *KLRF1* | 0.47 | <1.00E-16 | <1.00E-15 | 0.46 | 0.48 |
| *ZNF610* | 0.47 | <1.00E-16 | <1.00E-15 | 0.48 | 0.47 |
| *DTX3* | 0.58 | <1.00E-16 | <1.00E-15 | 0.59 | 0.57 |
| *RBM6* | 0.69 | <1.00E-16 | <1.00E-15 | 0.7 | 0.69 |
| *ADAM23* | 0.43 | <1.00E-16 | <1.00E-15 | 0.42 | 0.44 |
| *SOCS6* | 1.74 | <1.00E-16 | <1.00E-15 | 1.75 | 1.72 |
| *UBOX5* | 0.74 | <1.00E-16 | <1.00E-15 | 0.74 | 0.75 |
| *CD300LF* | 2.5 | <1.00E-16 | <1.00E-15 | 2.54 | 2.45 |
| *NCAPD2* | 0.7 | <1.00E-16 | <1.00E-15 | 0.7 | 0.69 |
| *TYSND1* | 0.73 | <1.00E-16 | <1.00E-15 | 0.72 | 0.73 |
| *DAB2* | 2.38 | <1.00E-16 | <1.00E-15 | 2.34 | 2.42 |
| *LCN10* | 0.5 | <1.00E-16 | <1.00E-15 | 0.51 | 0.49 |
| *GSDML* | 0.54 | <1.00E-16 | <1.00E-15 | 0.54 | 0.53 |
| *STX16* | 0.73 | <1.00E-16 | <1.00E-15 | 0.72 | 0.73 |
| *C14orf138* | 0.68 | <1.00E-16 | <1.00E-15 | 0.69 | 0.68 |
| *HSP90AA1* | 1.5 | <1.00E-16 | <1.00E-15 | 1.51 | 1.49 |
| *WDR22* | 0.75 | <1.00E-16 | <1.00E-15 | 0.74 | 0.75 |
| *WSB2* | 1.95 | <1.00E-16 | <1.00E-15 | 1.97 | 1.93 |
| *GNG12* | 4.04 | <1.00E-16 | <1.00E-15 | 3.95 | 4.18 |
| *NDUFV2* | 1.55 | <1.00E-16 | <1.00E-15 | 1.54 | 1.56 |
| *ROBO1* | 0.47 | <1.00E-16 | <1.00E-15 | 0.47 | 0.46 |
| *ADAM9* | 2.44 | <1.00E-16 | <1.00E-15 | 2.41 | 2.49 |
| *OCM* | 0.38 | <1.00E-16 | <1.00E-15 | 0.38 | 0.37 |
| *LOC129293* | 0.51 | <1.00E-16 | <1.00E-15 | 0.51 | 0.52 |
| *POLM* | 0.72 | <1.00E-16 | <1.00E-15 | 0.71 | 0.72 |
| *PPP4R1* | 1.41 | <1.00E-16 | <1.00E-15 | 1.42 | 1.4 |
| *TRIM52* | 0.63 | <1.00E-16 | <1.00E-15 | 0.62 | 0.63 |
| *CHD6* | 0.62 | <1.00E-16 | <1.00E-15 | 0.62 | 0.63 |
| *DYNLL1* | 1.4 | <1.00E-16 | <1.00E-15 | 1.39 | 1.41 |
| *PVR* | 2.82 | <1.00E-16 | <1.00E-15 | 2.86 | 2.77 |
| *ARGLU1* | 0.62 | <1.00E-16 | <1.00E-15 | 0.62 | 0.61 |
| *CCR2* | 3.85 | <1.00E-16 | <1.00E-15 | 3.92 | 3.76 |
| *ATP5C1* | 1.48 | <1.00E-16 | <1.00E-15 | 1.49 | 1.47 |
| *C5orf43* | 1.45 | <1.00E-16 | <1.00E-15 | 1.44 | 1.46 |
| *PXDN* | 0.46 | <1.00E-16 | <1.00E-15 | 0.46 | 0.45 |
| *ZNF544* | 0.67 | <1.00E-16 | <1.00E-15 | 0.68 | 0.67 |
| *U2AF1L4* | 0.69 | <1.00E-16 | <1.00E-15 | 0.69 | 0.69 |
| *KLC4* | 0.63 | <1.00E-16 | <1.00E-15 | 0.62 | 0.63 |
| *SLC35B1* | 1.81 | <1.00E-16 | <1.00E-15 | 1.82 | 1.79 |
| *ARFGAP3* | 1.41 | <1.00E-16 | <1.00E-15 | 1.42 | 1.4 |
| *UBE2W* | 1.53 | <1.00E-16 | <1.00E-15 | 1.54 | 1.52 |
| *TIMP1* | 2.11 | <1.00E-16 | <1.00E-15 | 2.12 | 2.08 |
| *ZNF167* | 0.61 | <1.00E-16 | <1.00E-15 | 0.61 | 0.6 |
| *ZDHHC5* | 1.25 | <1.00E-16 | <1.00E-15 | 1.25 | 1.24 |
| *PRDX3* | 1.66 | <1.00E-16 | <1.00E-15 | 1.65 | 1.68 |
| *SYNE1* | 0.52 | <1.00E-16 | <1.00E-15 | 0.53 | 0.52 |
| *MAP3K12* | 0.6 | <1.00E-16 | <1.00E-15 | 0.59 | 0.6 |
| *RBM33* | 0.65 | <1.00E-16 | <1.00E-15 | 0.65 | 0.66 |
| *DNAJB11* | 1.99 | <1.00E-16 | <1.00E-15 | 1.98 | 2.01 |
| *C14orf2* | 1.3 | <1.00E-16 | <1.00E-15 | 1.29 | 1.3 |
| *PLA2G6* | 0.5 | <1.00E-16 | <1.00E-15 | 0.49 | 0.5 |
| *STYXL1* | 1.48 | <1.00E-16 | <1.00E-15 | 1.48 | 1.47 |
| *MYL6* | 1.59 | <1.00E-16 | <1.00E-15 | 1.58 | 1.6 |
| *PTPN12* | 1.8 | <1.00E-16 | <1.00E-15 | 1.81 | 1.79 |
| *COMMD10* | 1.5 | <1.00E-16 | <1.00E-15 | 1.5 | 1.51 |
| *NRIP2* | 0.56 | <1.00E-16 | <1.00E-15 | 0.56 | 0.55 |
| *ATP1B3* | 2.11 | <1.00E-16 | <1.00E-15 | 2.13 | 2.09 |
| *DNAJB6* | 1.51 | <1.00E-16 | <1.00E-15 | 1.52 | 1.5 |
| *KRT73* | 0.29 | <1.00E-16 | <1.00E-15 | 0.29 | 0.29 |
| *SLC25A26* | 0.8 | <1.00E-16 | <1.00E-15 | 0.79 | 0.8 |
| *SLC31A1* | 2 | <1.00E-16 | <1.00E-15 | 2.02 | 1.98 |
| *ZNF767* | 0.57 | <1.00E-16 | <1.00E-15 | 0.58 | 0.57 |
| *PI16* | 0.52 | <1.00E-16 | <1.00E-15 | 0.52 | 0.52 |
| *CARD8* | 0.63 | <1.00E-16 | <1.00E-15 | 0.63 | 0.64 |
| *TMED2* | 1.39 | <1.00E-16 | <1.00E-15 | 1.4 | 1.39 |
| *TSHZ3* | 2.93 | <1.00E-16 | <1.00E-15 | 2.97 | 2.89 |
| *CSDA* | 1.45 | <1.00E-16 | <1.00E-15 | 1.45 | 1.46 |
| *FLJ12529* | 0.78 | <1.00E-16 | <1.00E-15 | 0.78 | 0.78 |
| *SIRT3* | 0.61 | <1.00E-16 | <1.00E-15 | 0.61 | 0.61 |
| *PLEKHB2* | 1.62 | <1.00E-16 | <1.00E-15 | 1.61 | 1.63 |
| *C9orf37* | 0.68 | <1.00E-16 | <1.00E-15 | 0.68 | 0.68 |
| *ZNF831* | 0.44 | <1.00E-16 | <1.00E-15 | 0.44 | 0.44 |
| *CCDC47* | 1.56 | <1.00E-16 | <1.00E-15 | 1.57 | 1.55 |
| *UBTF* | 0.71 | <1.00E-16 | <1.00E-15 | 0.71 | 0.71 |
| *KIAA1529* | 0.51 | <1.00E-16 | <1.00E-15 | 0.5 | 0.51 |
| *FLJ45909* | 0.63 | <1.00E-16 | <1.00E-15 | 0.64 | 0.63 |
| *RAB1A* | 1.66 | <1.00E-16 | <1.00E-15 | 1.67 | 1.65 |
| *MAP2K6* | 0.53 | <1.00E-16 | <1.00E-15 | 0.53 | 0.52 |
| *MAGEF1* | 0.71 | <1.00E-16 | <1.00E-15 | 0.7 | 0.71 |
| *ERCC6* | 0.56 | <1.00E-16 | <1.00E-15 | 0.56 | 0.56 |
| *CHP* | 1.49 | <1.00E-16 | <1.00E-15 | 1.48 | 1.49 |
| *GHITM* | 1.39 | <1.00E-16 | <1.00E-15 | 1.39 | 1.38 |
| *MTO1* | 0.77 | <1.00E-16 | <1.00E-15 | 0.77 | 0.77 |
| *LAT2* | 1.66 | <1.00E-16 | <1.00E-15 | 1.67 | 1.65 |
| *CALCOCO1* | 0.57 | <1.00E-16 | <1.00E-15 | 0.57 | 0.57 |
| *FLYWCH1* | 0.65 | <1.00E-16 | <1.00E-15 | 0.65 | 0.65 |
| *BCL7A* | 0.61 | <1.00E-16 | <1.00E-15 | 0.61 | 0.61 |
| *CDK2AP1* | 1.73 | <1.00E-16 | <1.00E-15 | 1.74 | 1.72 |
| *EVPL* | 0.43 | <1.00E-16 | <1.00E-15 | 0.43 | 0.42 |
| *CD300A* | 0.54 | <1.00E-16 | <1.00E-15 | 0.54 | 0.55 |
| *ZNF276* | 0.7 | <1.00E-16 | <1.00E-15 | 0.7 | 0.7 |
| *DBI* | 1.59 | <1.00E-16 | <1.00E-15 | 1.59 | 1.58 |
| *CSAD* | 0.58 | <1.00E-16 | <1.00E-15 | 0.58 | 0.57 |
| *FBXO32* | 0.51 | <1.00E-16 | <1.00E-15 | 0.51 | 0.51 |
| *KIAA0831* | 0.71 | <1.00E-16 | <1.00E-15 | 0.72 | 0.71 |
| *ZNF570* | 0.65 | <1.00E-16 | <1.00E-15 | 0.65 | 0.65 |
| *APOBEC3D* | 0.7 | <1.00E-16 | <1.00E-15 | 0.7 | 0.7 |
| *GPATCH8* | 0.75 | <1.00E-16 | <1.00E-15 | 0.75 | 0.75 |
| *ZNF835* | 0.63 | <1.00E-16 | <1.00E-15 | 0.63 | 0.63 |
| *MGC33894* | 0.54 | <1.00E-16 | <1.00E-15 | 0.54 | 0.55 |
| *RPN2* | 1.65 | <1.00E-16 | <1.00E-15 | 1.64 | 1.66 |
| *PSMA1* | 1.44 | <1.00E-16 | <1.00E-15 | 1.44 | 1.45 |
| *ZNF673* | 0.61 | <1.00E-16 | <1.00E-15 | 0.61 | 0.62 |
| *ATP6V1G2* | 0.53 | <1.00E-16 | <1.00E-15 | 0.53 | 0.54 |
| *PCSK4* | 0.52 | <1.00E-16 | <1.00E-15 | 0.52 | 0.52 |
| *C4orf30* | 0.66 | <1.00E-16 | <1.00E-15 | 0.66 | 0.66 |
| *FLVCR2* | 3.54 | <1.00E-16 | <1.00E-15 | 3.51 | 3.57 |
| *UGDH* | 1.41 | <1.00E-16 | <1.00E-15 | 1.42 | 1.41 |
| *UQCRFS1* | 1.6 | <1.00E-16 | <1.00E-15 | 1.6 | 1.61 |
| *MTCH2* | 1.63 | <1.00E-16 | <1.00E-15 | 1.64 | 1.63 |
| *OLR1* | 9.9 | <1.00E-16 | <1.00E-15 | 9.98 | 9.81 |
| *ARHGAP18* | 3.54 | <1.00E-16 | <1.00E-15 | 3.56 | 3.51 |
| *ENPP2* | 6.17 | <1.00E-16 | <1.00E-15 | 6.11 | 6.24 |
| *KDELR2* | 1.49 | <1.00E-16 | <1.00E-15 | 1.48 | 1.49 |
| *POMP* | 2.03 | <1.00E-16 | <1.00E-15 | 2.02 | 2.04 |
| *DSE* | 3.75 | <1.00E-16 | <1.00E-15 | 3.72 | 3.78 |
| *ACSL3* | 1.59 | <1.00E-16 | <1.00E-15 | 1.59 | 1.58 |
| *LDLRAD3* | 3.64 | <1.00E-16 | <1.00E-15 | 3.62 | 3.67 |
| *CSTA* | 1.88 | <1.00E-16 | <1.00E-15 | 1.87 | 1.89 |
| *SEC11C* | 1.48 | <1.00E-16 | <1.00E-15 | 1.48 | 1.49 |
| *PSPC1* | 0.75 | <1.00E-16 | <1.00E-15 | 0.74 | 0.75 |
| *PARP15* | 0.5 | <1.00E-16 | <1.00E-15 | 0.5 | 0.51 |
| *ETF1* | 1.45 | <1.00E-16 | <1.00E-15 | 1.45 | 1.45 |
| *PDE6B* | 0.66 | <1.00E-16 | <1.00E-15 | 0.66 | 0.66 |
| *CHD2* | 0.63 | <1.00E-16 | <1.00E-15 | 0.63 | 0.64 |
| *TMEM192* | 1.3 | <1.00E-16 | <1.00E-15 | 1.3 | 1.3 |
| *DCUN1D3* | 2.34 | <1.00E-16 | <1.00E-15 | 2.35 | 2.33 |
| *RAD23B* | 1.34 | <1.00E-16 | <1.00E-15 | 1.34 | 1.34 |
| *PON2* | 1.59 | <1.00E-16 | <1.00E-15 | 1.59 | 1.58 |
| *TMEM165* | 1.71 | <1.00E-16 | <1.00E-15 | 1.71 | 1.72 |
| *PNPO* | 1.42 | <1.00E-16 | <1.00E-15 | 1.42 | 1.42 |
| *ATP2C1* | 1.76 | <1.00E-16 | <1.00E-15 | 1.76 | 1.76 |
| *GPRASP1* | 0.47 | <1.00E-16 | <1.00E-15 | 0.47 | 0.47 |
| *SRPRB* | 1.31 | <1.00E-16 | <1.00E-15 | 1.31 | 1.31 |
| *TTC12* | 0.6 | <1.00E-16 | <1.00E-15 | 0.6 | 0.6 |
| *DDX11* | 0.71 | <1.00E-16 | <1.00E-15 | 0.71 | 0.71 |
| *C2orf34* | 0.72 | <1.00E-16 | <1.00E-15 | 0.72 | 0.72 |
| *PACSIN3* | 0.45 | <1.00E-16 | <1.00E-15 | 0.45 | 0.45 |
| *SLC23A3* | 0.54 | <1.00E-16 | <1.00E-15 | 0.54 | 0.55 |
| *IL1B* | 5.94 | <1.00E-16 | <1.00E-15 | 5.97 | 5.91 |
| *RCAN1* | 1.51 | <1.00E-16 | <1.00E-15 | 1.52 | 1.51 |
| *FLJ10404* | 0.61 | <1.00E-16 | <1.00E-15 | 0.61 | 0.6 |
| *ANKZF1* | 0.55 | <1.00E-16 | <1.00E-15 | 0.55 | 0.55 |
| *HSPA1L* | 0.66 | <1.00E-16 | <1.00E-15 | 0.66 | 0.66 |
| *NSUN5B* | 0.6 | <1.00E-16 | <1.00E-15 | 0.59 | 0.6 |
| *ARID1B* | 0.72 | <1.00E-16 | <1.00E-15 | 0.72 | 0.73 |
| *B3GNT5* | 4.68 | <1.00E-16 | <1.00E-15 | 4.7 | 4.65 |
| *ZNF212* | 0.74 | <1.00E-16 | <1.00E-15 | 0.74 | 0.74 |
| *ZNF275* | 0.72 | <1.00E-16 | <1.00E-15 | 0.72 | 0.73 |
| *C15orf52* | 0.61 | <1.00E-16 | <1.00E-15 | 0.61 | 0.61 |
| *GSTO1* | 1.83 | <1.00E-16 | <1.00E-15 | 1.83 | 1.84 |
| *ADAM17* | 1.4 | <1.00E-16 | <1.00E-15 | 1.4 | 1.4 |
| *NAT5* | 1.44 | <1.00E-16 | <1.00E-15 | 1.44 | 1.44 |
| *XPC* | 0.7 | <1.00E-16 | <1.00E-15 | 0.7 | 0.7 |
| *SLC14A1* | 0.37 | <1.00E-16 | <1.00E-15 | 0.37 | 0.37 |
| *FOXO4* | 0.66 | <1.00E-16 | <1.00E-15 | 0.67 | 0.66 |
| *POLH* | 0.71 | <1.00E-16 | <1.00E-15 | 0.71 | 0.71 |
| *REEP3* | 1.84 | <1.00E-16 | <1.00E-15 | 1.84 | 1.84 |
| *OGT* | 0.58 | <1.00E-16 | <1.00E-15 | 0.58 | 0.58 |
| *THRA* | 0.59 | <1.00E-16 | <1.00E-15 | 0.59 | 0.59 |
| *MICALL2* | 3.02 | <1.00E-16 | <1.00E-15 | 3.01 | 3.02 |
| *SH3BGRL* | 1.44 | <1.00E-16 | <1.00E-15 | 1.44 | 1.44 |
| *PARP16* | 0.65 | <1.00E-16 | <1.00E-15 | 0.65 | 0.65 |
| *CCDC102B* | 2.93 | <1.00E-16 | <1.00E-15 | 2.93 | 2.94 |
| *CRB3* | 0.51 | <1.00E-16 | <1.00E-15 | 0.51 | 0.51 |
| *MPHOSPH6* | 1.8 | <1.00E-16 | <1.00E-15 | 1.8 | 1.8 |
| *COL5A3* | 0.43 | <1.00E-16 | <1.00E-15 | NA | NA |
| *FAM118A* | 0.68 | <1.00E-16 | <1.00E-15 | NA | NA |
| *TCL1A* | 0.55 | 2.22E-16 | 3.40E-15 | 0.49 | 0.65 |
| *ATP6V0D2* | 0.27 | 2.22E-16 | 3.40E-15 | 0.22 | 0.37 |
| *SHF* | 0.58 | 2.22E-16 | 3.40E-15 | 0.63 | 0.51 |
| *FLJ23834* | 0.55 | 2.22E-16 | 3.40E-15 | 0.6 | 0.5 |
| *GZMK* | 0.61 | 2.22E-16 | 3.40E-15 | 0.57 | 0.66 |
| *SCML4* | 0.63 | 2.22E-16 | 3.40E-15 | 0.59 | 0.68 |
| *IQGAP2* | 0.68 | 2.22E-16 | 3.40E-15 | 0.65 | 0.73 |
| *C14orf149* | 0.74 | 2.22E-16 | 3.40E-15 | 0.71 | 0.78 |
| *RNF38* | 0.75 | 2.22E-16 | 3.40E-15 | 0.72 | 0.79 |
| *SIGLEC11* | 6.15 | 2.22E-16 | 3.40E-15 | 7.8 | 4.61 |
| *NDUFS4* | 1.41 | 2.22E-16 | 3.40E-15 | 1.35 | 1.49 |
| *SMAD4* | 0.73 | 2.22E-16 | 3.40E-15 | 0.7 | 0.77 |
| *BCL2L11* | 0.65 | 2.22E-16 | 3.40E-15 | 0.62 | 0.7 |
| *MEPCE* | 0.74 | 2.22E-16 | 3.40E-15 | 0.72 | 0.77 |
| *ZNF586* | 0.68 | 2.22E-16 | 3.40E-15 | 0.65 | 0.72 |
| *GLT1D1* | 2.55 | 2.22E-16 | 3.40E-15 | 2.31 | 2.89 |
| *PPT2* | 0.52 | 2.22E-16 | 3.40E-15 | 0.56 | 0.48 |
| *NDUFV3* | 1.3 | 2.22E-16 | 3.40E-15 | 1.33 | 1.26 |
| *DECR1* | 1.58 | 2.22E-16 | 3.40E-15 | 1.64 | 1.49 |
| *RFFL* | 0.73 | 2.22E-16 | 3.40E-15 | 0.71 | 0.76 |
| *HSPA9* | 1.41 | 2.22E-16 | 3.40E-15 | 1.45 | 1.36 |
| *C7orf58* | 2.08 | 2.22E-16 | 3.40E-15 | 2.2 | 1.93 |
| *XPNPEP1* | 1.58 | 2.22E-16 | 3.40E-15 | 1.64 | 1.51 |
| *SP4* | 0.58 | 2.22E-16 | 3.40E-15 | 0.56 | 0.61 |
| *BTN3A1* | 0.62 | 2.22E-16 | 3.40E-15 | 0.6 | 0.65 |
| *CEP68* | 0.55 | 2.22E-16 | 3.40E-15 | 0.53 | 0.58 |
| *FKBP1A* | 2.04 | 2.22E-16 | 3.40E-15 | 2.15 | 1.92 |
| *FAM114A1* | 1.76 | 2.22E-16 | 3.40E-15 | 1.69 | 1.85 |
| *ATP5B* | 1.66 | 2.22E-16 | 3.40E-15 | 1.72 | 1.59 |
| *RBM5* | 0.7 | 2.22E-16 | 3.40E-15 | 0.68 | 0.72 |
| *TNFRSF12A* | 5.84 | 2.22E-16 | 3.40E-15 | 6.55 | 5.07 |
| *SLC22A5* | 1.77 | 2.22E-16 | 3.40E-15 | 1.84 | 1.69 |
| *KIAA0892* | 0.74 | 2.22E-16 | 3.40E-15 | 0.73 | 0.76 |
| *MRPS7* | 1.51 | 2.22E-16 | 3.40E-15 | 1.55 | 1.46 |
| *COX5B* | 1.51 | 2.22E-16 | 3.40E-15 | 1.48 | 1.56 |
| *VSIG4* | 6.22 | 2.22E-16 | 3.40E-15 | 6.89 | 5.47 |
| *MRPS23* | 1.33 | 2.22E-16 | 3.40E-15 | 1.34 | 1.3 |
| *HEMK1* | 0.68 | 2.22E-16 | 3.40E-15 | 0.69 | 0.66 |
| *PIK3IP1* | 0.58 | 2.22E-16 | 3.40E-15 | 0.56 | 0.6 |
| *ZNF785* | 0.6 | 2.22E-16 | 3.40E-15 | 0.61 | 0.58 |
| *SFI1* | 0.59 | 2.22E-16 | 3.40E-15 | 0.58 | 0.61 |
| *RAP2A* | 1.86 | 2.22E-16 | 3.40E-15 | 1.91 | 1.79 |
| *RAD51L3* | 0.73 | 2.22E-16 | 3.40E-15 | 0.72 | 0.74 |
| *NFATC2* | 0.57 | 2.22E-16 | 3.40E-15 | 0.58 | 0.55 |
| *UBE2J1* | 1.86 | 2.22E-16 | 3.40E-15 | 1.91 | 1.8 |
| *LGR4* | 0.51 | 2.22E-16 | 3.40E-15 | 0.52 | 0.5 |
| *HOXB4* | 0.71 | 2.22E-16 | 3.40E-15 | 0.72 | 0.7 |
| *UNC5CL* | 0.53 | 2.22E-16 | 3.40E-15 | 0.54 | 0.51 |
| *GTF2A2* | 1.38 | 2.22E-16 | 3.40E-15 | 1.37 | 1.4 |
| *DHRS13* | 0.57 | 2.22E-16 | 3.40E-15 | 0.58 | 0.56 |
| *MAPRE1* | 1.33 | 2.22E-16 | 3.40E-15 | 1.32 | 1.34 |
| *DENND1C* | 0.67 | 2.22E-16 | 3.40E-15 | 0.68 | 0.66 |
| *TPM2* | 0.41 | 2.22E-16 | 3.40E-15 | 0.42 | 0.39 |
| *KLRG1* | 0.51 | 2.22E-16 | 3.40E-15 | 0.5 | 0.52 |
| *FADS1* | 2.55 | 2.22E-16 | 3.40E-15 | 2.61 | 2.47 |
| *ZMAT1* | 0.52 | 2.22E-16 | 3.40E-15 | 0.53 | 0.51 |
| *PGK1* | 1.7 | 2.22E-16 | 3.40E-15 | 1.73 | 1.67 |
| *AP3B1* | 1.28 | 2.22E-16 | 3.40E-15 | 1.29 | 1.27 |
| *LOC144983* | 0.62 | 2.22E-16 | 3.40E-15 | 0.63 | 0.61 |
| *ANKRD44* | 0.7 | 2.22E-16 | 3.40E-15 | 0.69 | 0.71 |
| *ERCC5* | 0.72 | 2.22E-16 | 3.40E-15 | 0.72 | 0.71 |
| *ARHGAP24* | 0.51 | 2.22E-16 | 3.40E-15 | 0.5 | 0.52 |
| *KIT* | 0.48 | 2.22E-16 | 3.40E-15 | 0.47 | 0.48 |
| *ZNF793* | 0.54 | 2.22E-16 | 3.40E-15 | 0.53 | 0.55 |
| *UNC13B* | 1.97 | 2.22E-16 | 3.40E-15 | 1.94 | 2 |
| *NPTN* | 1.81 | 2.22E-16 | 3.40E-15 | 1.83 | 1.79 |
| *CEP110* | 0.68 | 2.22E-16 | 3.40E-15 | 0.68 | 0.67 |
| *GCA* | 2.1 | 2.22E-16 | 3.40E-15 | 2.13 | 2.07 |
| *PTGR1* | 2.34 | 2.22E-16 | 3.40E-15 | 2.31 | 2.38 |
| *BRD8* | 0.73 | 2.22E-16 | 3.40E-15 | 0.73 | 0.72 |
| *SLFN11* | 1.59 | 2.22E-16 | 3.40E-15 | 1.58 | 1.6 |
| *ROBO3* | 0.6 | 2.22E-16 | 3.40E-15 | 0.6 | 0.61 |
| *C16orf54* | 0.59 | 2.22E-16 | 3.40E-15 | 0.59 | 0.58 |
| *HADHB* | 1.38 | 2.22E-16 | 3.40E-15 | 1.38 | 1.38 |
| *MYOM1* | 0.62 | 2.22E-16 | 3.40E-15 | 0.62 | 0.62 |
| *FAM73B* | 0.69 | 2.22E-16 | 3.40E-15 | 0.69 | 0.7 |
| *WDR41* | 1.51 | 2.22E-16 | 3.40E-15 | 1.51 | 1.51 |
| *MGEA5* | 0.61 | 2.22E-16 | 3.40E-15 | 0.61 | 0.6 |
| *PLAC8* | 0.66 | 4.44E-16 | 6.33E-15 | 0.59 | 0.75 |
| *FCRLB* | 2.4 | 4.44E-16 | 6.33E-15 | 2.89 | 1.91 |
| *KIAA0652* | 0.83 | 4.44E-16 | 6.33E-15 | 0.8 | 0.88 |
| *CEP72* | 0.68 | 4.44E-16 | 6.33E-15 | 0.64 | 0.73 |
| *ZNF766* | 0.66 | 4.44E-16 | 6.33E-15 | 0.63 | 0.71 |
| *LRRC37B* | 0.61 | 4.44E-16 | 6.33E-15 | 0.57 | 0.66 |
| *SMARCD1* | 0.8 | 4.44E-16 | 6.33E-15 | 0.78 | 0.83 |
| *TAF13* | 2.05 | 4.44E-16 | 6.33E-15 | 2.23 | 1.84 |
| *ORMDL3* | 0.68 | 4.44E-16 | 6.33E-15 | 0.65 | 0.72 |
| *SNAP23* | 1.3 | 4.44E-16 | 6.33E-15 | 1.34 | 1.26 |
| *CLPB* | 2.01 | 4.44E-16 | 6.33E-15 | 2.18 | 1.83 |
| *WNT10B* | 0.5 | 4.44E-16 | 6.33E-15 | 0.54 | 0.45 |
| *KCTD10* | 1.32 | 4.44E-16 | 6.33E-15 | 1.36 | 1.27 |
| *HSF4* | 0.59 | 4.44E-16 | 6.33E-15 | 0.56 | 0.63 |
| *CLEC2D* | 0.51 | 4.44E-16 | 6.33E-15 | 0.48 | 0.54 |
| *GAS7* | 0.51 | 4.44E-16 | 6.33E-15 | 0.48 | 0.55 |
| *PRKRIP1* | 0.75 | 4.44E-16 | 6.33E-15 | 0.73 | 0.77 |
| *BST1* | 2.07 | 4.44E-16 | 6.33E-15 | 1.95 | 2.23 |
| *CBY1* | 0.61 | 4.44E-16 | 6.33E-15 | 0.58 | 0.64 |
| *SPRED1* | 5.05 | 4.44E-16 | 6.33E-15 | 5.73 | 4.33 |
| *CASP2* | 0.71 | 4.44E-16 | 6.33E-15 | 0.69 | 0.74 |
| *B4GALT2* | 2.04 | 4.44E-16 | 6.33E-15 | 2.15 | 1.91 |
| *ATP5G3* | 1.9 | 4.44E-16 | 6.33E-15 | 1.99 | 1.79 |
| *PTPRO* | 3.14 | 4.44E-16 | 6.33E-15 | 3.41 | 2.84 |
| *TM7SF3* | 1.26 | 4.44E-16 | 6.33E-15 | 1.24 | 1.28 |
| *ABHD4* | 0.66 | 4.44E-16 | 6.33E-15 | 0.65 | 0.68 |
| *L3MBTL3* | 0.68 | 4.44E-16 | 6.33E-15 | 0.66 | 0.7 |
| *TMEFF1* | 2.03 | 4.44E-16 | 6.33E-15 | 2.11 | 1.93 |
| *ERLIN1* | 1.31 | 4.44E-16 | 6.33E-15 | 1.33 | 1.29 |
| *GPR114* | 0.58 | 4.44E-16 | 6.33E-15 | 0.6 | 0.56 |
| *ADHFE1* | 0.43 | 4.44E-16 | 6.33E-15 | 0.41 | 0.45 |
| *ATHL1* | 0.49 | 4.44E-16 | 6.33E-15 | 0.47 | 0.51 |
| *ZSCAN2* | 0.65 | 4.44E-16 | 6.33E-15 | 0.63 | 0.67 |
| *MYST4* | 0.64 | 4.44E-16 | 6.33E-15 | 0.65 | 0.62 |
| *NDUFB5* | 1.33 | 4.44E-16 | 6.33E-15 | 1.35 | 1.31 |
| *TUBGCP6* | 0.65 | 4.44E-16 | 6.33E-15 | 0.64 | 0.67 |
| *SNED1* | 0.5 | 4.44E-16 | 6.33E-15 | 0.49 | 0.52 |
| *MRPS35* | 1.5 | 4.44E-16 | 6.33E-15 | 1.53 | 1.46 |
| *ACIN1* | 0.79 | 4.44E-16 | 6.33E-15 | 0.8 | 0.78 |
| *ASXL1* | 0.72 | 4.44E-16 | 6.33E-15 | 0.71 | 0.73 |
| *DNM1L* | 1.33 | 4.44E-16 | 6.33E-15 | 1.34 | 1.31 |
| *MAP2K5* | 0.76 | 4.44E-16 | 6.33E-15 | 0.77 | 0.75 |
| *CCT6A* | 1.43 | 4.44E-16 | 6.33E-15 | 1.44 | 1.4 |
| *TLR1* | 2.11 | 4.44E-16 | 6.33E-15 | 2.06 | 2.17 |
| *IDH1* | 2.4 | 4.44E-16 | 6.33E-15 | 2.34 | 2.48 |
| *MRPL15* | 1.5 | 4.44E-16 | 6.33E-15 | 1.52 | 1.48 |
| *FRAT1* | 0.58 | 4.44E-16 | 6.33E-15 | 0.57 | 0.59 |
| *ZNF827* | 0.57 | 4.44E-16 | 6.33E-15 | 0.58 | 0.56 |
| *ANKRD10* | 0.75 | 4.44E-16 | 6.33E-15 | 0.75 | 0.76 |
| *ZNF512* | 0.7 | 4.44E-16 | 6.33E-15 | 0.7 | 0.71 |
| *MGAT2* | 1.26 | 4.44E-16 | 6.33E-15 | 1.25 | 1.27 |
| *RGL4* | 0.6 | 4.44E-16 | 6.33E-15 | 0.59 | 0.6 |
| *TXK* | 0.56 | 4.44E-16 | 6.33E-15 | 0.56 | 0.57 |
| *KIAA1407* | 0.64 | 4.44E-16 | 6.33E-15 | 0.64 | 0.63 |
| *STK38* | 0.6 | 4.44E-16 | 6.33E-15 | 0.59 | 0.61 |
| *CCDC69* | 0.71 | 4.44E-16 | 6.33E-15 | 0.71 | 0.72 |
| *CLK4* | 0.59 | 4.44E-16 | 6.33E-15 | 0.59 | 0.6 |
| *DPP3* | 1.63 | 4.44E-16 | 6.33E-15 | 1.62 | 1.65 |
| *VPS52* | 0.84 | 4.44E-16 | 6.33E-15 | 0.84 | 0.84 |
| *ZNF514* | 0.64 | 4.44E-16 | 6.33E-15 | 0.64 | 0.65 |
| *MYST2* | 0.73 | 4.44E-16 | 6.33E-15 | 0.73 | 0.73 |
| *AKAP8L* | 0.72 | 4.44E-16 | 6.33E-15 | 0.72 | 0.73 |
| *SEC61A1* | 1.43 | 4.44E-16 | 6.33E-15 | 1.43 | 1.42 |
| *PELI2* | 0.53 | 4.44E-16 | 6.33E-15 | 0.53 | 0.54 |
| *PRR3* | 0.74 | 4.44E-16 | 6.33E-15 | 0.73 | 0.74 |
| *MECP2* | 0.64 | 4.44E-16 | 6.33E-15 | 0.64 | 0.64 |
| *POFUT2* | 0.74 | 4.44E-16 | 6.33E-15 | 0.74 | 0.74 |
| *ATP6V1A* | 1.76 | 4.44E-16 | 6.33E-15 | 1.76 | 1.76 |
| *KIAA0515* | 0.61 | 4.44E-16 | 6.33E-15 | 0.61 | 0.6 |
| *NXF1* | 0.74 | 4.44E-16 | 6.33E-15 | 0.73 | 0.74 |
| *CAPRIN2* | 0.64 | 4.44E-16 | 6.33E-15 | 0.64 | 0.64 |
| *ALOX5AP* | 2.44 | 6.66E-16 | 9.21E-15 | 2.98 | 1.92 |
| *FLJ44968* | 0.62 | 6.66E-16 | 9.21E-15 | 0.66 | 0.57 |
| *GIMAP1* | 0.62 | 6.66E-16 | 9.21E-15 | 0.58 | 0.66 |
| *GDPD3* | 0.5 | 6.66E-16 | 9.21E-15 | 0.53 | 0.46 |
| *MAML1* | 0.73 | 6.66E-16 | 9.21E-15 | 0.7 | 0.76 |
| *DEXI* | 0.76 | 6.66E-16 | 9.21E-15 | 0.75 | 0.79 |
| *ACTR2* | 1.34 | 6.66E-16 | 9.21E-15 | 1.37 | 1.3 |
| *ARRDC2* | 0.52 | 6.66E-16 | 9.21E-15 | 0.49 | 0.56 |
| *CLEC1A* | 4.39 | 6.66E-16 | 9.21E-15 | 4.9 | 3.82 |
| *CABLES1* | 0.31 | 6.66E-16 | 9.21E-15 | 0.28 | 0.34 |
| *ZNF304* | 0.71 | 6.66E-16 | 9.21E-15 | 0.69 | 0.73 |
| *ADAMTS10* | 0.5 | 6.66E-16 | 9.21E-15 | 0.52 | 0.47 |
| *C17orf58* | 2.19 | 6.66E-16 | 9.21E-15 | 2.31 | 2.04 |
| *ANKRA2* | 0.67 | 6.66E-16 | 9.21E-15 | 0.65 | 0.69 |
| *SIRPG* | 0.49 | 6.66E-16 | 9.21E-15 | 0.47 | 0.51 |
| *ATG16L2* | 0.51 | 6.66E-16 | 9.21E-15 | 0.49 | 0.52 |
| *LTBP3* | 0.56 | 6.66E-16 | 9.21E-15 | 0.58 | 0.55 |
| *SFRS5* | 0.68 | 6.66E-16 | 9.21E-15 | 0.67 | 0.69 |
| *TUBG2* | 0.55 | 6.66E-16 | 9.21E-15 | 0.54 | 0.57 |
| *ANXA5* | 2.12 | 6.66E-16 | 9.21E-15 | 2.06 | 2.19 |
| *SAMD3* | 0.51 | 6.66E-16 | 9.21E-15 | 0.49 | 0.52 |
| *CCDC65* | 0.4 | 6.66E-16 | 9.21E-15 | 0.39 | 0.41 |
| *CD160* | 0.46 | 6.66E-16 | 9.21E-15 | 0.46 | 0.47 |
| *TROAP* | 0.5 | 6.66E-16 | 9.21E-15 | 0.51 | 0.5 |
| *HIGD1A* | 1.76 | 6.66E-16 | 9.21E-15 | 1.74 | 1.79 |
| *NCR3* | 0.5 | 6.66E-16 | 9.21E-15 | 0.49 | 0.51 |
| *DBP* | 0.48 | 6.66E-16 | 9.21E-15 | 0.49 | 0.47 |
| *TERF2* | 0.76 | 6.66E-16 | 9.21E-15 | 0.75 | 0.76 |
| *CHD3* | 0.61 | 6.66E-16 | 9.21E-15 | 0.61 | 0.6 |
| *UBE2D1* | 1.76 | 6.66E-16 | 9.21E-15 | 1.78 | 1.75 |
| *MAX* | 0.86 | 8.88E-16 | 1.18E-14 | 0.83 | 0.91 |
| *BTN3A3* | 0.63 | 8.88E-16 | 1.18E-14 | 0.59 | 0.7 |
| *ARHGAP20* | 3.36 | 8.88E-16 | 1.18E-14 | 4.06 | 2.65 |
| *LIM2* | 0.46 | 8.88E-16 | 1.18E-14 | 0.51 | 0.4 |
| *IGBP1* | 0.71 | 8.88E-16 | 1.18E-14 | 0.68 | 0.75 |
| *NUDT7* | 0.6 | 8.88E-16 | 1.18E-14 | 0.57 | 0.66 |
| *CD79A* | 0.55 | 8.88E-16 | 1.18E-14 | 0.51 | 0.6 |
| *PCIF1* | 0.7 | 8.88E-16 | 1.18E-14 | 0.67 | 0.73 |
| *PSAT1* | 0.52 | 8.88E-16 | 1.18E-14 | 0.48 | 0.57 |
| *CCNDBP1* | 0.74 | 8.88E-16 | 1.18E-14 | 0.72 | 0.78 |
| *TIPARP* | 0.49 | 8.88E-16 | 1.18E-14 | 0.45 | 0.53 |
| *NRF1* | 0.74 | 8.88E-16 | 1.18E-14 | 0.72 | 0.77 |
| *CNOT8* | 0.76 | 8.88E-16 | 1.18E-14 | 0.74 | 0.79 |
| *KLHL2* | 1.31 | 8.88E-16 | 1.18E-14 | 1.35 | 1.27 |
| *TRAT1* | 0.49 | 8.88E-16 | 1.18E-14 | 0.46 | 0.54 |
| *SART3* | 0.83 | 8.88E-16 | 1.18E-14 | 0.81 | 0.84 |
| *AQP9* | 8.53 | 8.88E-16 | 1.18E-14 | 10.3 | 6.75 |
| *COL19A1* | 0.47 | 8.88E-16 | 1.18E-14 | 0.51 | 0.44 |
| *NCF2* | 2.7 | 8.88E-16 | 1.18E-14 | 2.94 | 2.44 |
| *C5orf41* | 0.55 | 8.88E-16 | 1.18E-14 | 0.53 | 0.58 |
| *ATF7IP* | 0.67 | 8.88E-16 | 1.18E-14 | 0.65 | 0.69 |
| *ANKMY1* | 0.62 | 8.88E-16 | 1.18E-14 | 0.64 | 0.6 |
| *MAP2K7* | 0.74 | 8.88E-16 | 1.18E-14 | 0.73 | 0.76 |
| *ZNF548* | 0.6 | 8.88E-16 | 1.18E-14 | 0.62 | 0.58 |
| *RBL2* | 0.62 | 8.88E-16 | 1.18E-14 | 0.6 | 0.63 |
| *UHRF2* | 0.67 | 8.88E-16 | 1.18E-14 | 0.67 | 0.69 |
| *BLK* | 0.51 | 8.88E-16 | 1.18E-14 | 0.5 | 0.53 |
| *SLC25A3* | 1.31 | 8.88E-16 | 1.18E-14 | 1.33 | 1.3 |
| *MTERFD2* | 0.6 | 8.88E-16 | 1.18E-14 | 0.58 | 0.61 |
| *SIRT4* | 0.58 | 8.88E-16 | 1.18E-14 | 0.59 | 0.57 |
| *CMTM6* | 1.65 | 8.88E-16 | 1.18E-14 | 1.67 | 1.62 |
| *FLJ21438* | 0.6 | 8.88E-16 | 1.18E-14 | 0.6 | 0.62 |
| *ACACB* | 0.51 | 8.88E-16 | 1.18E-14 | 0.52 | 0.5 |
| *CDR2* | 0.66 | 8.88E-16 | 1.18E-14 | 0.65 | 0.67 |
| *UGP2* | 1.34 | 8.88E-16 | 1.18E-14 | 1.34 | 1.33 |
| *ZNF783* | 0.75 | 8.88E-16 | 1.18E-14 | 0.75 | 0.74 |
| *ITSN2* | 0.76 | 8.88E-16 | 1.18E-14 | 0.76 | 0.75 |
| *SURF4* | 1.43 | 8.88E-16 | 1.18E-14 | 1.44 | 1.42 |
| *C5orf4* | 0.37 | 8.88E-16 | 1.18E-14 | 0.37 | 0.38 |
| *CLDN15* | 0.59 | 8.88E-16 | 1.18E-14 | 0.59 | 0.6 |
| *PLAG1* | 0.55 | 8.88E-16 | 1.18E-14 | 0.56 | 0.55 |
| *SETD5* | 0.73 | 8.88E-16 | 1.18E-14 | 0.74 | 0.73 |
| *ZBTB3* | 0.76 | 8.88E-16 | 1.18E-14 | 0.76 | 0.76 |
| *ARHGEF1* | 0.63 | 8.88E-16 | 1.18E-14 | 0.63 | 0.63 |
| *XCL2* | 0.58 | 8.88E-16 | 1.18E-14 | NA | NA |

^a^Gene symbol is provided for gene identification,

^b^Fold change for overall response to stimulation analysis (all stimulated samples vs. all unstimulated samples; Stim/Unstim, S/U)

^c^P-value and false discovery rate (FDR) for the overall analysis

^d^Fold change for High responders, stimulated vs. unstimulated samples (HS/HU)

^e^Fold change for Low responders, stimulated vs. unstimulated samples (LS/LU)
