# Supplementary material for: Genome-Wide Characterization of Transcriptional Patterns in High and Low Antibody Responders to Rubella Vaccination
Source: PLoS One. 2013 May 1;8(5):e62149. doi: 10.1371/journal.pone.0062149 (PMC3641062; doi:10.1371/journal.pone.0062149)
Supplement: Table S2 — Response to rubella virus stimulation in high vs. low antibody responders to rubella vaccination (genes with p<0.05). (DOCX) [file pone.0062149.s002.docx]

**Supplemental Table 2**. Response to rubella virus stimulation in high vs. low antibody responders to rubella vaccination (genes with p<0.05)

| **Gene symbol**^a^ | **FC_int**^b^ | **P-value_int**^c^ | **FDR_int**^c^ | **FC_low**^d^ | **FC_high**^e^ |
| --- | --- | --- | --- | --- | --- |
| *EMR3* | 0.18 | 1.46E-08 | 0.0002 | 2.29 | 0.41 |
| *HIPK4* | 0.46 | 3.49E-07 | 0.002 | 1.46 | 0.67 |
| *C11orf9* | 0.76 | 2.40E-06 | 0.009 | 0.7 | 0.53 |
| *RAMP1* | 0.67 | 2.49E-06 | 0.009 | 1.13 | 0.76 |
| *RHEBL1* | 1.57 | 7.53E-06 | 0.022 | 0.53 | 0.83 |
| *TRPV6* | 0.45 | 3.05E-05 | 0.065 | 0.76 | 0.34 |
| *MKRN1* | 1.19 | 3.17E-05 | 0.065 | 0.78 | 0.93 |
| *NINJ1* | 1.67 | 7.90E-05 | 0.141 | 0.58 | 0.97 |
| *S100A13* | 0.52 | 9.89E-05 | 0.151 | 1.64 | 0.86 |
| *HLA-A* | 1.48 | 0.0001 | 0.151 | 0.59 | 0.87 |
| *ASS1* | 0.28 | 0.0002 | 0.197 | 1.45 | 0.41 |
| *DLL1* | 2.14 | 0.0002 | 0.198 | 0.27 | 0.57 |
| *MYL5* | 0.77 | 0.0002 | 0.212 | 1.14 | 0.87 |
| *B2M* | 1.3 | 0.0002 | 0.222 | 0.71 | 0.92 |
| *WDR45L* | 1.13 | 0.0003 | 0.263 | 0.9 | 1.02 |
| *TRAPPC4* | 1.15 | 0.0003 | 0.265 | 0.9 | 1.03 |
| *TEAD3* | 0.55 | 0.0003 | 0.265 | 1.54 | 0.85 |
| *TMEM176A* | 2.26 | 0.0003 | 0.265 | 0.95 | 2.14 |
| *ASAH1* | 1.23 | 0.0004 | 0.265 | 0.8 | 0.98 |
| *GLI3* | 0.41 | 0.0004 | 0.265 | 1.26 | 0.52 |
| *LOC401399* | 2.18 | 0.0004 | 0.273 | 0.52 | 1.13 |
| *MEFV* | 2.72 | 0.0004 | 0.273 | 0.49 | 1.34 |
| *HLA-B* | 1.35 | 0.001 | 0.294 | 0.78 | 1.06 |
| *EGLN3* | 1.67 | 0.001 | 0.294 | 0.66 | 1.11 |
| *FLJ45422* | 1.51 | 0.001 | 0.294 | 0.71 | 1.07 |
| *NWD1* | 0.22 | 0.001 | 0.294 | 7.35 | 1.65 |
| *TMEM176B* | 2.09 | 0.001 | 0.294 | 0.83 | 1.73 |
| *PTH2R* | 4.64 | 0.001 | 0.303 | 0.47 | 2.18 |
| *C17orf78* | 4.96 | 0.001 | 0.303 | 0.31 | 1.55 |
| *NOS1* | 0.31 | 0.001 | 0.305 | 0.93 | 0.29 |
| *TRPM2* | 1.85 | 0.001 | 0.305 | 0.47 | 0.86 |
| *HIPK2* | 0.76 | 0.001 | 0.32 | 1.2 | 0.91 |
| *CLP1* | 1.15 | 0.001 | 0.32 | 0.95 | 1.1 |
| *TMEM60* | 1.24 | 0.001 | 0.351 | 0.9 | 1.11 |
| *ACOT4* | 0.62 | 0.001 | 0.351 | 1.4 | 0.87 |
| *MGAM* | 0.41 | 0.001 | 0.372 | 4.5 | 1.83 |
| *VCAN* | 0.57 | 0.001 | 0.372 | 4.86 | 2.77 |
| *IFI27* | 14.11 | 0.001 | 0.372 | 0.17 | 2.38 |
| *C20orf26* | 0.21 | 0.001 | 0.372 | 1.86 | 0.38 |
| *ADAMTS2* | 2.14 | 0.001 | 0.372 | 0.78 | 1.68 |
| *TMCC3* | 0.71 | 0.001 | 0.372 | 1.32 | 0.94 |
| *PIP5KL1* | 0.4 | 0.001 | 0.372 | 1.2 | 0.48 |
| *ZDHHC4* | 1.11 | 0.001 | 0.372 | 0.91 | 1.01 |
| *PRH2* | 0.31 | 0.001 | 0.374 | 0.94 | 0.29 |
| *HLA-C* | 1.42 | 0.001 | 0.382 | 0.74 | 1.06 |
| *TCN2* | 2.01 | 0.001 | 0.39 | 0.38 | 0.77 |
| *HINT3* | 1.2 | 0.001 | 0.413 | 1.08 | 1.29 |
| *GPM6A* | 1.84 | 0.002 | 0.455 | 0.43 | 0.79 |
| *SLC35D2* | 1.18 | 0.002 | 0.455 | 0.92 | 1.08 |
| *GPBAR1* | 2.03 | 0.002 | 0.507 | 0.45 | 0.92 |
| *SLC5A9* | 0.28 | 0.002 | 0.514 | 1.18 | 0.33 |
| *GPRC5D* | 2.93 | 0.002 | 0.528 | 0.3 | 0.89 |
| *XCR1* | 3.99 | 0.002 | 0.534 | 0.42 | 1.68 |
| *SPATA6* | 0.55 | 0.002 | 0.553 | 1.1 | 0.61 |
| *GRAMD1B* | 1.36 | 0.002 | 0.553 | 0.58 | 0.79 |
| *HLA-E* | 1.3 | 0.002 | 0.553 | 0.67 | 0.88 |
| *LPIN3* | 1.74 | 0.002 | 0.567 | 0.84 | 1.46 |
| *LCP2* | 1.18 | 0.002 | 0.567 | 0.86 | 1.02 |
| *BPGM* | 1.21 | 0.002 | 0.567 | 0.9 | 1.09 |
| *PSMA8* | 0.42 | 0.002 | 0.578 | 0.51 | 0.22 |
| *INTU* | 2.03 | 0.002 | 0.578 | 0.76 | 1.55 |
| *GREB1* | 0.58 | 0.003 | 0.578 | 1 | 0.58 |
| *C1R* | 2.02 | 0.003 | 0.589 | 0.65 | 1.32 |
| *EME1* | 1.36 | 0.003 | 0.598 | 0.69 | 0.94 |
| *LOC440567* | 0.68 | 0.003 | 0.612 | 1.63 | 1.12 |
| *APOE* | 1.7 | 0.003 | 0.612 | 0.63 | 1.08 |
| *PRRC1* | 0.86 | 0.003 | 0.612 | 1.2 | 1.03 |
| *GRN* | 1.52 | 0.003 | 0.628 | 0.73 | 1.11 |
| *HLA-F* | 1.37 | 0.003 | 0.637 | 0.67 | 0.91 |
| *IRF2* | 1.26 | 0.003 | 0.637 | 0.74 | 0.93 |
| *FSTL1* | 0.37 | 0.003 | 0.637 | 5.03 | 1.84 |
| *C16orf75* | 1.39 | 0.003 | 0.655 | 0.78 | 1.09 |
| *KIAA1024* | 0.66 | 0.003 | 0.662 | 1.59 | 1.05 |
| *GLTPD2* | 1.57 | 0.004 | 0.682 | 1.07 | 1.67 |
| *CADM4* | 1.4 | 0.004 | 0.705 | 0.84 | 1.17 |
| *ADAT3* | 1.39 | 0.004 | 0.723 | 0.71 | 0.99 |
| *FAM59A* | 1.86 | 0.004 | 0.73 | 1.06 | 1.98 |
| *CCR8* | 2.11 | 0.004 | 0.737 | 0.35 | 0.73 |
| *GNG4* | 0.35 | 0.004 | 0.747 | 0.84 | 0.3 |
| *ABI3* | 1.53 | 0.004 | 0.747 | 0.49 | 0.75 |
| *RBM11* | 1.6 | 0.004 | 0.747 | 0.45 | 0.71 |
| *CCDC150* | 2.77 | 0.004 | 0.747 | 0.41 | 1.15 |
| *RECQL4* | 1.28 | 0.004 | 0.747 | 0.62 | 0.79 |
| *KIAA1244* | 0.26 | 0.004 | 0.747 | 10.37 | 2.71 |
| *PLA2G2D* | 0.37 | 0.004 | 0.747 | 0.59 | 0.22 |
| *GPRIN3* | 0.74 | 0.004 | 0.747 | 1.81 | 1.35 |
| *LINCR* | 4.79 | 0.005 | 0.758 | 0.2 | 0.95 |
| *WDR66* | 0.41 | 0.005 | 0.758 | 1.26 | 0.52 |
| *SECTM1* | 2.19 | 0.005 | 0.758 | 0.58 | 1.27 |
| *SCIN* | 1.74 | 0.005 | 0.758 | 0.65 | 1.13 |
| *SSTR2* | 3.03 | 0.005 | 0.766 | 0.36 | 1.09 |
| *UTF1* | 2.32 | 0.005 | 0.766 | 1.43 | 3.31 |
| *LDLRAD2* | 0.49 | 0.005 | 0.787 | 1.12 | 0.54 |
| *APOBEC3B* | 3.04 | 0.005 | 0.788 | 0.71 | 2.15 |
| *ADPRHL1* | 0.73 | 0.005 | 0.803 | 0.99 | 0.72 |
| *LOC440836* | 2.4 | 0.005 | 0.803 | 0.58 | 1.4 |
| *NTAN1* | 1.26 | 0.005 | 0.803 | 0.71 | 0.89 |
| *MRP63* | 1.1 | 0.006 | 0.811 | 0.97 | 1.07 |
| *LOC150763* | 0.7 | 0.006 | 0.822 | 1.73 | 1.21 |
| *FKBPL* | 1.22 | 0.006 | 0.822 | 0.85 | 1.04 |
| *DIABLO* | 1.11 | 0.006 | 0.822 | 1.04 | 1.15 |
| *USP42* | 1.15 | 0.006 | 0.822 | 0.82 | 0.94 |
| *SLC45A4* | 1.42 | 0.006 | 0.822 | 0.66 | 0.93 |
| *GCH1* | 1.65 | 0.006 | 0.822 | 0.9 | 1.49 |
| *C9orf91* | 1.36 | 0.006 | 0.822 | 0.56 | 0.76 |
| *GSTK1* | 1.12 | 0.006 | 0.822 | 0.88 | 0.99 |
| *TSPAN3* | 1.14 | 0.006 | 0.822 | 0.94 | 1.07 |
| *NR1H3* | 1.79 | 0.006 | 0.822 | 0.46 | 0.82 |
| *NAV2* | 0.7 | 0.006 | 0.822 | 1.17 | 0.81 |
| *RRAGA* | 1.09 | 0.006 | 0.822 | 0.96 | 1.05 |
| *TRIP10* | 1.23 | 0.006 | 0.826 | 0.83 | 1.02 |
| *SERPING1* | 5.03 | 0.006 | 0.826 | 0.55 | 2.75 |
| *RNF114* | 1.12 | 0.007 | 0.885 | 0.83 | 0.93 |
| *NFE2L3* | 1.52 | 0.007 | 0.888 | 0.91 | 1.38 |
| *FZD2* | 1.89 | 0.007 | 0.888 | 0.56 | 1.07 |
| *WNK4* | 0.42 | 0.007 | 0.898 | 0.89 | 0.38 |
| *GADD45B* | 1.29 | 0.007 | 0.898 | 0.61 | 0.79 |
| *C2* | 2 | 0.007 | 0.898 | 0.95 | 1.9 |
| *SLC4A9* | 0.28 | 0.007 | 0.898 | 1.44 | 0.4 |
| *PLXNA4* | 0.67 | 0.008 | 0.902 | 1.21 | 0.81 |
| *AMDHD2* | 1.52 | 0.008 | 0.902 | 0.53 | 0.81 |
| *SLC25A10* | 1.37 | 0.008 | 0.902 | 0.67 | 0.92 |
| *FCER2* | 0.52 | 0.008 | 0.902 | 2.99 | 1.55 |
| *CR1* | 0.54 | 0.008 | 0.902 | 1 | 0.54 |
| *LPCAT3* | 1.13 | 0.008 | 0.902 | 0.98 | 1.1 |
| *FAM90A1* | 2.5 | 0.008 | 0.902 | 0.75 | 1.87 |
| *MAX* | 1.09 | 0.008 | 0.902 | 0.83 | 0.91 |
| *IL4I1* | 2.59 | 0.008 | 0.902 | 0.66 | 1.71 |
| *ACP2* | 1.5 | 0.008 | 0.902 | 1.01 | 1.52 |
| *GFI1B* | 0.48 | 0.008 | 0.902 | 1.29 | 0.61 |
| *SIGLEC1* | 3.05 | 0.008 | 0.902 | 0.56 | 1.72 |
| *ADCY1* | 2.13 | 0.008 | 0.902 | 0.33 | 0.7 |
| *TMEM132E* | 0.59 | 0.008 | 0.902 | 0.8 | 0.48 |
| *SAMD4A* | 1.99 | 0.008 | 0.902 | 0.48 | 0.95 |
| *IFITM3* | 5.55 | 0.009 | 0.916 | 0.43 | 2.41 |
| *KIF18A* | 1.84 | 0.009 | 0.916 | 0.51 | 0.94 |
| *EIF2C1* | 0.72 | 0.009 | 0.92 | 1.28 | 0.92 |
| *LOC198437* | 0.39 | 0.009 | 0.92 | 1.29 | 0.5 |
| *PRSS35* | 0.65 | 0.009 | 0.92 | 0.76 | 0.5 |
| *FAM125B* | 1.57 | 0.009 | 0.92 | 0.48 | 0.74 |
| *SPN* | 0.74 | 0.009 | 0.92 | 1.48 | 1.1 |
| *PTPRG* | 0.3 | 0.009 | 0.92 | 0.92 | 0.28 |
| *GALNT8* | 2.02 | 0.009 | 0.92 | 0.63 | 1.27 |
| *NARF* | 1.16 | 0.01 | 0.92 | 0.78 | 0.91 |
| *CC2D2A* | 0.53 | 0.01 | 0.92 | 0.89 | 0.47 |
| *FAM132A* | 0.51 | 0.01 | 0.92 | 0.74 | 0.37 |
| *LYSMD2* | 1.28 | 0.01 | 0.92 | 0.95 | 1.22 |
| *GNGT2* | 1.36 | 0.01 | 0.92 | 0.66 | 0.9 |
| *OTOF* | 5.5 | 0.01 | 0.92 | 0.21 | 1.14 |
| *SAT1* | 1.34 | 0.01 | 0.92 | 0.61 | 0.82 |
| *ABHD12* | 1.41 | 0.01 | 0.92 | 0.68 | 0.97 |
| *CORIN* | 2.48 | 0.01 | 0.92 | 0.15 | 0.37 |
| *CBWD6* | 0.55 | 0.01 | 0.92 | 1.08 | 0.6 |
| *ITGB3* | 0.5 | 0.01 | 0.925 | 2.98 | 1.49 |
| *XIRP1* | 2.79 | 0.01 | 0.925 | 1.11 | 3.1 |
| *FOXC1* | 2.1 | 0.01 | 0.929 | 0.49 | 1.02 |
| *LOC402057* | 0.58 | 0.01 | 0.929 | 1.03 | 0.59 |
| *S100A1* | 0.55 | 0.011 | 0.929 | 1.04 | 0.57 |
| *SLC38A5* | 1.55 | 0.011 | 0.929 | 0.55 | 0.86 |
| *PIWIL4* | 1.41 | 0.011 | 0.929 | 0.76 | 1.08 |
| *CFB* | 5.81 | 0.011 | 0.929 | 0.56 | 3.24 |
| *KIAA1881* | 0.74 | 0.011 | 0.929 | 0.85 | 0.62 |
| *ATG10* | 1.28 | 0.011 | 0.929 | 0.89 | 1.13 |
| *NOS2A* | 2.15 | 0.011 | 0.929 | 0.35 | 0.76 |
| *FAM108C1* | 1.39 | 0.011 | 0.929 | 0.71 | 0.99 |
| *BOK* | 0.67 | 0.011 | 0.929 | 0.9 | 0.6 |
| *MLANA* | 0.56 | 0.011 | 0.929 | 0.77 | 0.43 |
| *IQGAP3* | 0.57 | 0.011 | 0.929 | 1.72 | 0.97 |
| *RHOD* | 0.4 | 0.011 | 0.929 | 1.01 | 0.41 |
| *UVRAG* | 1.18 | 0.011 | 0.929 | 0.79 | 0.93 |
| *FSCN3* | 2.57 | 0.011 | 0.929 | 0.4 | 1.02 |
| *TMEM180* | 1.31 | 0.012 | 0.929 | 0.73 | 0.95 |
| *OLIG2* | 3.97 | 0.012 | 0.929 | 0.38 | 1.51 |
| *ZNF330* | 1.11 | 0.012 | 0.929 | 1.01 | 1.12 |
| *FAM23A* | 0.11 | 0.012 | 0.929 | 32.89 | 3.57 |
| *GPR175* | 1.36 | 0.012 | 0.929 | 0.56 | 0.76 |
| *C19orf23* | 0.75 | 0.012 | 0.929 | 1.54 | 1.16 |
| *TMPRSS9* | 1.52 | 0.012 | 0.929 | 0.59 | 0.9 |
| *PAX8* | 2.37 | 0.012 | 0.929 | 0.33 | 0.78 |
| *LUZP1* | 0.74 | 0.012 | 0.929 | 1.17 | 0.86 |
| *ATOX1* | 1.34 | 0.012 | 0.929 | 0.89 | 1.19 |
| *KRT79* | 0.29 | 0.012 | 0.929 | 9.13 | 2.61 |
| *GDE1* | 1.17 | 0.012 | 0.929 | 1.13 | 1.32 |
| *ANKRD27* | 1.14 | 0.012 | 0.929 | 0.78 | 0.89 |
| *ANXA4* | 1.34 | 0.012 | 0.929 | 0.98 | 1.31 |
| *PNKD* | 1.37 | 0.012 | 0.929 | 1.09 | 1.5 |
| *F8A1* | 1.24 | 0.013 | 0.929 | 0.79 | 0.98 |
| *ACOX2* | 0.17 | 0.013 | 0.929 | 39.95 | 6.92 |
| *EIF4E3* | 1.23 | 0.013 | 0.929 | 0.87 | 1.08 |
| *COQ7* | 0.88 | 0.013 | 0.929 | 1.12 | 0.98 |
| *FAM83D* | 1.45 | 0.013 | 0.929 | 0.51 | 0.74 |
| *PCBP1* | 1.11 | 0.013 | 0.929 | 0.86 | 0.95 |
| *SLC17A3* | 3.34 | 0.013 | 0.929 | 0.32 | 1.08 |
| *BAALC* | 1.72 | 0.013 | 0.929 | 0.26 | 0.44 |
| *KLHDC7B* | 1.97 | 0.013 | 0.929 | 0.7 | 1.38 |
| *CEACAM19* | 0.7 | 0.013 | 0.929 | 1.41 | 0.99 |
| *MST150* | 1.2 | 0.013 | 0.929 | 1 | 1.21 |
| *CPE* | 0.35 | 0.013 | 0.929 | 11.15 | 3.88 |
| *CD1B* | 0.09 | 0.013 | 0.929 | 69.23 | 6.16 |
| *CTSH* | 1.36 | 0.013 | 0.929 | 1.22 | 1.66 |
| *CPLX3* | 0.22 | 0.013 | 0.929 | 2.31 | 0.51 |
| *HLA-G* | 1.46 | 0.013 | 0.929 | 0.72 | 1.05 |
| *RNF130* | 1.25 | 0.013 | 0.929 | 0.95 | 1.18 |
| *S100A5* | 0.46 | 0.014 | 0.929 | 3.97 | 1.83 |
| *SYNGR2* | 1.34 | 0.014 | 0.929 | 0.83 | 1.11 |
| *TSNAXIP1* | 0.63 | 0.014 | 0.929 | 1.07 | 0.68 |
| *KLHDC9* | 0.38 | 0.014 | 0.929 | 0.79 | 0.3 |
| *ARHGAP22* | 1.32 | 0.014 | 0.929 | 0.96 | 1.27 |
| *ADH5* | 1.16 | 0.014 | 0.929 | 0.81 | 0.93 |
| *HIST1H2BG* | 0.52 | 0.014 | 0.929 | 0.49 | 0.26 |
| *MADCAM1* | 0.54 | 0.014 | 0.929 | 1.23 | 0.66 |
| *PSAP* | 1.26 | 0.014 | 0.929 | 0.94 | 1.18 |
| *PPIL5* | 1.15 | 0.014 | 0.929 | 1.03 | 1.19 |
| *TRADD* | 1.18 | 0.014 | 0.929 | 0.76 | 0.89 |
| *CACNA1E* | 0.38 | 0.014 | 0.929 | 0.67 | 0.26 |
| *UCK1* | 1.18 | 0.014 | 0.929 | 0.77 | 0.9 |
| *TNFSF10* | 2.49 | 0.014 | 0.929 | 0.62 | 1.55 |
| *GPRIN1* | 1.53 | 0.014 | 0.929 | 0.94 | 1.44 |
| *DOCK3* | 0.61 | 0.015 | 0.929 | 2.52 | 1.52 |
| *IRF8* | 1.23 | 0.015 | 0.929 | 0.76 | 0.94 |
| *SLC29A1* | 1.32 | 0.015 | 0.929 | 0.66 | 0.88 |
| *COL15A1* | 0.51 | 0.015 | 0.929 | 3.46 | 1.75 |
| *GFER* | 1.13 | 0.015 | 0.929 | 0.84 | 0.94 |
| *COPA* | 0.73 | 0.015 | 0.929 | 1.47 | 1.07 |
| *IFITM2* | 1.92 | 0.015 | 0.929 | 0.5 | 0.96 |
| *ARL6IP6* | 1.12 | 0.015 | 0.929 | 1 | 1.13 |
| *SLC9A7* | 0.62 | 0.015 | 0.929 | 2.63 | 1.63 |
| *CYBASC3* | 1.46 | 0.015 | 0.929 | 0.49 | 0.71 |
| *APOA2* | 0.44 | 0.015 | 0.929 | 1.3 | 0.57 |
| *CDS2* | 1.13 | 0.015 | 0.929 | 0.8 | 0.9 |
| *IL18BP* | 1.55 | 0.015 | 0.929 | 0.65 | 1 |
| *COP1* | 1.33 | 0.015 | 0.929 | 0.66 | 0.87 |
| *IFI30* | 1.47 | 0.015 | 0.929 | 1.05 | 1.54 |
| *EIF4G3* | 0.73 | 0.015 | 0.929 | 1.72 | 1.25 |
| *LGALS3BP* | 2.56 | 0.016 | 0.929 | 0.63 | 1.61 |
| *NAV1* | 0.64 | 0.016 | 0.929 | 2 | 1.28 |
| *DIO1* | 0.5 | 0.016 | 0.929 | 1.25 | 0.63 |
| *ZNF622* | 1.12 | 0.016 | 0.929 | 0.93 | 1.05 |
| *SLC35E3* | 1.21 | 0.016 | 0.929 | 0.83 | 1.01 |
| *SLC17A5* | 1.14 | 0.016 | 0.929 | 1.12 | 1.28 |
| *TOR1AIP2* | 0.72 | 0.016 | 0.929 | 1.49 | 1.07 |
| *LAG3* | 1.99 | 0.016 | 0.929 | 0.49 | 0.98 |
| *OLIG1* | 4.15 | 0.016 | 0.929 | 0.1 | 0.4 |
| *TCHH* | 0.36 | 0.016 | 0.929 | 4.08 | 1.48 |
| *MLXIPL* | 0.42 | 0.016 | 0.929 | 9.6 | 4 |
| *PANK2* | 1.14 | 0.016 | 0.929 | 0.9 | 1.02 |
| *AXL* | 4.16 | 0.016 | 0.929 | 0.37 | 1.52 |
| *CXCL13* | 2.6 | 0.016 | 0.929 | 0.43 | 1.11 |
| *DPF2* | 1.14 | 0.016 | 0.929 | 0.73 | 0.83 |
| *LOC642587* | 0.48 | 0.016 | 0.929 | 0.87 | 0.42 |
| *PLAC8* | 1.26 | 0.017 | 0.929 | 0.59 | 0.75 |
| *HEXA* | 1.25 | 0.017 | 0.929 | 0.72 | 0.9 |
| *VSIG8* | 0.54 | 0.017 | 0.929 | 0.71 | 0.39 |
| *ZNRF3* | 0.78 | 0.017 | 0.929 | 1.55 | 1.21 |
| *C1orf187* | 0.59 | 0.017 | 0.929 | 1.09 | 0.64 |
| *PTPN7* | 0.74 | 0.017 | 0.929 | 1.38 | 1.02 |
| *LPGAT1* | 0.73 | 0.017 | 0.929 | 1.53 | 1.11 |
| *ANAPC13* | 1.18 | 0.017 | 0.929 | 0.88 | 1.05 |
| *STX5* | 1.13 | 0.017 | 0.929 | 0.83 | 0.94 |
| *LMAN1* | 0.85 | 0.017 | 0.929 | 1.32 | 1.12 |
| *FKBP11* | 1.21 | 0.017 | 0.929 | 0.66 | 0.8 |
| *CKB* | 1.42 | 0.017 | 0.929 | 0.61 | 0.87 |
| *SCAMP5* | 1.65 | 0.017 | 0.929 | 1.07 | 1.77 |
| *CXCL11* | 4.31 | 0.017 | 0.929 | 0.79 | 3.39 |
| *APOC1* | 1.61 | 0.017 | 0.929 | 0.76 | 1.22 |
| *PRB3* | 2.59 | 0.017 | 0.932 | 0.31 | 0.81 |
| *POMC* | 1.28 | 0.017 | 0.932 | 0.64 | 0.82 |
| *SIGLEC14* | 1.44 | 0.018 | 0.935 | 0.9 | 1.29 |
| *LCA5* | 0.5 | 0.018 | 0.935 | 0.94 | 0.47 |
| *C14orf83* | 1.3 | 0.018 | 0.935 | 0.82 | 1.06 |
| *THBS1* | 1.86 | 0.018 | 0.948 | 0.33 | 0.61 |
| *SEMA3B* | 1.92 | 0.018 | 0.955 | 0.59 | 1.12 |
| *TRIM72* | 0.49 | 0.018 | 0.955 | 4.64 | 2.3 |
| *STAP1* | 1.38 | 0.018 | 0.955 | 0.49 | 0.68 |
| *SP140* | 1.28 | 0.018 | 0.955 | 0.64 | 0.81 |
| *IL20RB* | 0.74 | 0.018 | 0.957 | 1.13 | 0.83 |
| *COL9A3* | 1.53 | 0.019 | 0.957 | 0.41 | 0.63 |
| *GRM4* | 2.51 | 0.019 | 0.957 | 0.55 | 1.38 |
| *FLJ43692* | 2.45 | 0.019 | 0.957 | 0.45 | 1.11 |
| *FAM46A* | 1.43 | 0.019 | 0.972 | 0.67 | 0.96 |
| *POU3F1* | 0.61 | 0.019 | 0.972 | 1.53 | 0.93 |
| *SIAH2* | 1.15 | 0.019 | 0.972 | 0.74 | 0.85 |
| *TACSTD2* | 0.11 | 0.019 | 0.972 | 55.76 | 6.4 |
| *ITPR1* | 0.82 | 0.019 | 0.972 | 1.04 | 0.86 |
| *C15orf39* | 1.23 | 0.019 | 0.972 | 0.8 | 0.99 |
| *ATP1B2* | 0.23 | 0.02 | 0.972 | 17.15 | 3.93 |
| *C2orf18* | 1.25 | 0.02 | 0.972 | 0.89 | 1.12 |
| *FAM3D* | 2.88 | 0.02 | 0.972 | 0.13 | 0.37 |
| *KIRREL2* | 0.6 | 0.02 | 0.972 | 1.24 | 0.74 |
| *LAMC1* | 0.68 | 0.02 | 0.972 | 2.18 | 1.48 |
| *ZDHHC19* | 0.61 | 0.02 | 0.972 | 0.87 | 0.53 |
| *FXYD6* | 1.84 | 0.02 | 0.972 | 0.31 | 0.57 |
| *NAPRT1* | 1.31 | 0.02 | 0.972 | 0.71 | 0.93 |
| *FLJ13137* | 0.51 | 0.02 | 0.972 | 1.4 | 0.71 |
| *RRAGB* | 1.14 | 0.02 | 0.972 | 0.81 | 0.92 |
| *GRIP1* | 1.74 | 0.021 | 0.972 | 0.51 | 0.88 |
| *PDE1B* | 0.67 | 0.021 | 0.972 | 2.1 | 1.41 |
| *GPR15* | 1.6 | 0.021 | 0.972 | 0.37 | 0.59 |
| *ATP1A1* | 0.69 | 0.021 | 0.972 | 1.7 | 1.17 |
| *UACA* | 1.4 | 0.021 | 0.972 | 1.42 | 2 |
| *TCP10L* | 0.48 | 0.021 | 0.972 | 1.18 | 0.57 |
| *MRPL28* | 1.21 | 0.021 | 0.972 | 0.98 | 1.19 |
| *PSMG4* | 1.18 | 0.021 | 0.972 | 0.74 | 0.88 |
| *U2AF1* | 1.12 | 0.021 | 0.972 | 0.93 | 1.04 |
| *KIAA0090* | 0.72 | 0.021 | 0.972 | 1.66 | 1.2 |
| *CDK8* | 1.11 | 0.021 | 0.972 | 0.97 | 1.08 |
| *ZNRF2* | 1.2 | 0.021 | 0.972 | 0.94 | 1.12 |
| *LEPR* | 0.72 | 0.021 | 0.972 | 1.55 | 1.12 |
| *OCLM* | 0.45 | 0.021 | 0.972 | 1.09 | 0.49 |
| *TMEM140* | 1.46 | 0.021 | 0.972 | 0.74 | 1.08 |
| *GHDC* | 1.28 | 0.021 | 0.972 | 0.61 | 0.79 |
| *GOSR1* | 1.09 | 0.021 | 0.972 | 0.97 | 1.05 |
| *DACH1* | 0.62 | 0.021 | 0.972 | 0.8 | 0.49 |
| *MED17* | 1.14 | 0.022 | 0.972 | 0.81 | 0.92 |
| *STAT5A* | 1.15 | 0.022 | 0.972 | 0.83 | 0.95 |
| *MIF4GD* | 1.18 | 0.022 | 0.972 | 0.75 | 0.89 |
| *DEPDC4* | 2.71 | 0.022 | 0.972 | 0.58 | 1.58 |
| *MANBA* | 1.16 | 0.022 | 0.972 | 1.01 | 1.17 |
| *LOC441476* | 0.52 | 0.022 | 0.972 | 0.51 | 0.26 |
| *CHST12* | 1.33 | 0.022 | 0.972 | 0.68 | 0.91 |
| *CDC42BPA* | 0.6 | 0.022 | 0.972 | 2.43 | 1.47 |
| *MED28* | 1.15 | 0.022 | 0.972 | 0.74 | 0.85 |
| *ZFAND3* | 1.11 | 0.022 | 0.972 | 1.03 | 1.14 |
| *LOC144097* | 1.17 | 0.022 | 0.973 | 0.81 | 0.95 |
| *KCNMB3* | 0.7 | 0.022 | 0.976 | 0.83 | 0.58 |
| *TFB2M* | 0.68 | 0.022 | 0.982 | 1.33 | 0.91 |
| *CHD1L* | 0.73 | 0.023 | 0.987 | 1.33 | 0.97 |
| *H3F3B* | 1.13 | 0.023 | 0.987 | 1 | 1.13 |
| *C15orf37* | 0.74 | 0.023 | 0.987 | 0.98 | 0.73 |
| *GNMT* | 1.6 | 0.023 | 0.987 | 0.5 | 0.8 |
| *C1orf141* | 0.67 | 0.023 | 0.987 | 0.96 | 0.64 |
| *HAS1* | 0.38 | 0.023 | 0.987 | 0.96 | 0.36 |
| *NEBL* | 1.8 | 0.023 | 0.993 | 0.21 | 0.38 |
| *ZNF385C* | 1.82 | 0.023 | 0.993 | 0.64 | 1.17 |
| *PAWR* | 1.4 | 0.023 | 0.993 | 0.52 | 0.73 |
| *TPSAB1* | 3.71 | 0.023 | 0.998 | 0.25 | 0.94 |
| *RAD51L1* | 1.19 | 0.024 | 0.998 | 0.74 | 0.88 |
| *SORBS3* | 1.24 | 0.024 | 0.998 | 0.74 | 0.92 |
| *KIAA1199* | 1.41 | 0.024 | 0.999 | 0.62 | 0.88 |
| *TRAPPC5* | 1.32 | 0.024 | 0.999 | 0.94 | 1.24 |
| *DHX58* | 2.16 | 0.024 | 0.999 | 0.69 | 1.49 |
| *C11orf16* | 0.48 | 0.024 | 0.999 | 1.56 | 0.75 |
| *APOL3* | 1.32 | 0.024 | 0.999 | 0.79 | 1.05 |
| *IL12RB1* | 1.27 | 0.024 | 0.999 | 0.66 | 0.83 |
| *RAP1GAP* | 0.21 | 0.024 | 0.999 | 19.25 | 4.08 |
| *TM2D3* | 1.1 | 0.024 | 0.999 | 0.81 | 0.89 |
| *RIPK4* | 0.7 | 0.025 | 0.999 | 0.61 | 0.42 |
| *RPP40* | 0.8 | 0.025 | 0.999 | 1.17 | 0.93 |
| *SCHIP1* | 2.55 | 0.025 | 0.999 | 0.84 | 2.15 |
| *HLA-A29-1* | 1.32 | 0.025 | 0.999 | 0.62 | 0.82 |
| *RIN1* | 1.65 | 0.025 | 0.999 | 0.73 | 1.2 |
| *THEM5* | 0.62 | 0.025 | 0.999 | 0.72 | 0.45 |
| *NR1I2* | 1.6 | 0.025 | 0.999 | 0.53 | 0.84 |
| *FCGRT* | 1.41 | 0.025 | 0.999 | 0.86 | 1.22 |
| *VANGL2* | 0.56 | 0.026 | 0.999 | 1.77 | 0.98 |
| *PILRA* | 1.47 | 0.026 | 0.999 | 1.1 | 1.62 |
| *SFT2D2* | 0.69 | 0.026 | 0.999 | 1.5 | 1.03 |
| *DARS2* | 0.72 | 0.026 | 0.999 | 1.84 | 1.32 |
| *KIAA1614* | 0.66 | 0.026 | 0.999 | 1.04 | 0.68 |
| *NFKBID* | 0.85 | 0.026 | 0.999 | 1.24 | 1.06 |
| *C16orf85* | 0.74 | 0.026 | 0.999 | 0.79 | 0.58 |
| *PCDHGA7* | 0.5 | 0.026 | 0.999 | 0.83 | 0.41 |
| *HOXA10* | 0.56 | 0.026 | 0.999 | 1.18 | 0.66 |
| *VANGL1* | 0.77 | 0.026 | 0.999 | 1.23 | 0.94 |
| *LIN52* | 0.78 | 0.026 | 0.999 | 0.93 | 0.73 |
| *PTGER2* | 1.5 | 0.026 | 0.999 | 0.91 | 1.36 |
| *HESX1* | 5.15 | 0.026 | 0.999 | 0.52 | 2.66 |
| *TMEM123* | 1.22 | 0.026 | 0.999 | 0.69 | 0.84 |
| *C1QTNF1* | 3.06 | 0.026 | 0.999 | 0.36 | 1.09 |
| *SCD* | 1.36 | 0.026 | 0.999 | 1.15 | 1.57 |
| *LHX4* | 0.66 | 0.026 | 0.999 | 0.76 | 0.5 |
| *SLC22A18* | 1.36 | 0.026 | 0.999 | 0.7 | 0.96 |
| *CACNA1A* | 1.84 | 0.027 | 0.999 | 0.69 | 1.26 |
| *TYROBP* | 1.42 | 0.027 | 0.999 | 1.04 | 1.48 |
| *PAPD4* | 1.15 | 0.027 | 0.999 | 0.93 | 1.07 |
| *TNFSF8* | 1.18 | 0.027 | 0.999 | 0.96 | 1.14 |
| *RILPL2* | 1.16 | 0.027 | 0.999 | 0.86 | 1 |
| *VMO1* | 2.38 | 0.027 | 0.999 | 0.58 | 1.39 |
| *C11orf17* | 1.24 | 0.027 | 0.999 | 1.23 | 1.51 |
| *CCR6* | 1.27 | 0.027 | 0.999 | 0.57 | 0.72 |
| *MAPK12* | 1.71 | 0.027 | 0.999 | 0.53 | 0.9 |
| *HTATIP2* | 1.13 | 0.027 | 0.999 | 1.17 | 1.32 |
| *ITM2B* | 1.23 | 0.028 | 0.999 | 0.64 | 0.79 |
| *TMEM149* | 1.24 | 0.028 | 0.999 | 0.82 | 1.01 |
| *ZBED1* | 1.31 | 0.028 | 0.999 | 0.59 | 0.77 |
| *ARMC10* | 1.11 | 0.028 | 0.999 | 1.04 | 1.16 |
| *KCTD3* | 0.67 | 0.028 | 0.999 | 1.68 | 1.12 |
| *MLPH* | 2.73 | 0.028 | 0.999 | 0.26 | 0.72 |
| *C7orf42* | 1.09 | 0.028 | 0.999 | 0.88 | 0.96 |
| *OPN1SW* | 0.65 | 0.028 | 0.999 | 1.49 | 0.96 |
| *MRFAP1* | 1.06 | 0.028 | 0.999 | 0.92 | 0.98 |
| *TMEM187* | 1.25 | 0.029 | 0.999 | 0.72 | 0.89 |
| *ZSCAN20* | 0.72 | 0.029 | 0.999 | 1.32 | 0.95 |
| *DBR1* | 1.12 | 0.029 | 0.999 | 0.97 | 1.09 |
| *C14orf166* | 1.14 | 0.029 | 0.999 | 0.98 | 1.11 |
| *GNG8* | 1.87 | 0.029 | 0.999 | 0.44 | 0.83 |
| *DAPP1* | 1.26 | 0.029 | 0.999 | 0.81 | 1.02 |
| *GIMAP2* | 1.15 | 0.029 | 0.999 | 0.72 | 0.83 |
| *TOM1* | 1.25 | 0.029 | 0.999 | 1.01 | 1.26 |
| *PPP1R15A* | 1.21 | 0.029 | 0.999 | 0.91 | 1.1 |
| *FRAP1* | 0.75 | 0.029 | 0.999 | 1.07 | 0.81 |
| *TNS1* | 1.38 | 0.029 | 0.999 | 1.01 | 1.4 |
| *ZNF823* | 1.32 | 0.029 | 0.999 | 0.5 | 0.66 |
| *PPM1J* | 0.7 | 0.03 | 0.999 | 1.59 | 1.11 |
| *NLRP2* | 0.73 | 0.03 | 0.999 | 2.22 | 1.62 |
| *MEIS1* | 0.58 | 0.03 | 0.999 | 1.14 | 0.67 |
| *JAG2* | 1.31 | 0.03 | 0.999 | 0.74 | 0.96 |
| *ZNF221* | 1.43 | 0.03 | 0.999 | 0.59 | 0.84 |
| *CENTA1* | 1.29 | 0.03 | 0.999 | 0.93 | 1.2 |
| *GNE* | 1.12 | 0.03 | 0.999 | 0.9 | 1.01 |
| *SLC47A1* | 1.49 | 0.03 | 0.999 | 0.51 | 0.75 |
| *MED10* | 1.12 | 0.03 | 0.999 | 0.81 | 0.9 |
| *ALOX5AP* | 0.64 | 0.03 | 0.999 | 2.98 | 1.92 |
| *OSBPL1A* | 1.34 | 0.03 | 0.999 | 0.82 | 1.1 |
| *PLEKHA7* | 1.46 | 0.03 | 0.999 | 1.09 | 1.59 |
| *LYSMD1* | 0.72 | 0.03 | 0.999 | 0.67 | 0.48 |
| *MPP1* | 1.34 | 0.031 | 0.999 | 0.91 | 1.22 |
| *SIGLEC5* | 1.39 | 0.031 | 0.999 | 1.08 | 1.5 |
| *MRPL24* | 0.72 | 0.031 | 0.999 | 1.72 | 1.23 |
| *SEMA4A* | 1.73 | 0.031 | 0.999 | 0.38 | 0.66 |
| *DAZAP2* | 1.1 | 0.031 | 0.999 | 0.91 | 1 |
| *GPR173* | 0.47 | 0.031 | 0.999 | 0.69 | 0.32 |
| *BSCL2* | 1.21 | 0.031 | 0.999 | 0.89 | 1.08 |
| *C16orf80* | 1.11 | 0.031 | 0.999 | 0.86 | 0.96 |
| *LOC201175* | 1.66 | 0.031 | 0.999 | 0.77 | 1.28 |
| *RPUSD1* | 1.16 | 0.032 | 0.999 | 0.97 | 1.13 |
| *DDI2* | 0.7 | 0.032 | 0.999 | 1.21 | 0.85 |
| *DHRS9* | 1.44 | 0.032 | 0.999 | 0.6 | 0.86 |
| *DNAH2* | 0.29 | 0.032 | 0.999 | 1.06 | 0.31 |
| *ITGA10* | 0.7 | 0.032 | 0.999 | 0.62 | 0.43 |
| *NUDT17* | 0.72 | 0.032 | 0.999 | 1.09 | 0.78 |
| *MYOM2* | 0.65 | 0.032 | 0.999 | 0.68 | 0.44 |
| *PCDHGB5* | 0.65 | 0.032 | 0.999 | 1.53 | 0.99 |
| *SNX33* | 1.26 | 0.032 | 0.999 | 0.94 | 1.18 |
| *TAGAP* | 1.27 | 0.032 | 0.999 | 0.54 | 0.68 |
| *PGRMC1* | 1.13 | 0.032 | 0.999 | 1.18 | 1.33 |
| *ATP10A* | 1.26 | 0.032 | 0.999 | 0.61 | 0.77 |
| *ARSA* | 1.22 | 0.033 | 0.999 | 0.75 | 0.92 |
| *MATK* | 0.66 | 0.033 | 0.999 | 2.16 | 1.42 |
| *CTSA* | 1.27 | 0.033 | 0.999 | 1.02 | 1.3 |
| *LRRC7* | 0.49 | 0.033 | 0.999 | 0.64 | 0.31 |
| *KIAA0133* | 0.71 | 0.033 | 0.999 | 1.28 | 0.92 |
| *BAHCC1* | 1.61 | 0.033 | 0.999 | 0.56 | 0.9 |
| *SPSB2* | 1.23 | 0.033 | 0.999 | 0.71 | 0.87 |
| *AHCYL1* | 0.74 | 0.033 | 0.999 | 1.62 | 1.19 |
| *PPFIBP2* | 1.2 | 0.033 | 0.999 | 0.81 | 0.97 |
| *H1F0* | 1.68 | 0.033 | 0.999 | 0.76 | 1.27 |
| *HSD17B14* | 1.51 | 0.034 | 0.999 | 0.39 | 0.59 |
| *CD68* | 1.33 | 0.034 | 0.999 | 1.1 | 1.46 |
| *GRM1* | 0.26 | 0.034 | 0.999 | 3.89 | 1.02 |
| *TRAIP* | 1.29 | 0.034 | 0.999 | 0.64 | 0.83 |
| *MRPS18A* | 1.18 | 0.034 | 0.999 | 0.98 | 1.16 |
| *SH3BP5* | 0.81 | 0.034 | 0.999 | 1.35 | 1.09 |
| *ANGPTL4* | 0.67 | 0.034 | 0.999 | 0.84 | 0.57 |
| *TMEM87A* | 1.1 | 0.034 | 0.999 | 1.03 | 1.14 |
| *SMTNL1* | 2.13 | 0.034 | 0.999 | 0.71 | 1.51 |
| *C7orf57* | 2.11 | 0.035 | 0.999 | 0.96 | 2.02 |
| *BAD* | 1.22 | 0.035 | 0.999 | 0.93 | 1.14 |
| *S100P* | 0.43 | 0.035 | 0.999 | 0.76 | 0.33 |
| *MARVELD3* | 2.54 | 0.035 | 0.999 | 0.39 | 1 |
| *NCF1* | 1.64 | 0.035 | 0.999 | 0.66 | 1.08 |
| *RNF34* | 1.12 | 0.035 | 0.999 | 0.76 | 0.84 |
| *GTF2F2* | 1.16 | 0.035 | 0.999 | 1.01 | 1.18 |
| *BZRAP1* | 0.78 | 0.035 | 0.999 | 0.7 | 0.54 |
| *TMEM14C* | 1.13 | 0.035 | 0.999 | 1.06 | 1.19 |
| *AATK* | 1.59 | 0.035 | 0.999 | 0.75 | 1.19 |
| *YY1* | 1.08 | 0.035 | 0.999 | 0.87 | 0.94 |
| *C10orf26* | 1.18 | 0.035 | 0.999 | 0.72 | 0.84 |
| *LIX1* | 0.39 | 0.035 | 0.999 | 0.77 | 0.3 |
| *CD38* | 2.33 | 0.035 | 0.999 | 0.55 | 1.29 |
| *BLOC1S2* | 1.13 | 0.035 | 0.999 | 0.99 | 1.12 |
| *LSM2* | 1.1 | 0.036 | 0.999 | 0.97 | 1.07 |
| *THOC3* | 1.22 | 0.036 | 0.999 | 0.81 | 0.99 |
| *MRTO4* | 0.69 | 0.036 | 0.999 | 1.98 | 1.37 |
| *PDXDC1* | 1.12 | 0.036 | 0.999 | 0.76 | 0.85 |
| *STAMBP* | 1.07 | 0.036 | 0.999 | 0.94 | 1.01 |
| *BBS5* | 1.25 | 0.036 | 0.999 | 0.87 | 1.09 |
| *ZNF555* | 1.17 | 0.036 | 0.999 | 0.83 | 0.97 |
| *AIRE* | 1.74 | 0.036 | 0.999 | 0.38 | 0.66 |
| *GAL3ST2* | 2.5 | 0.036 | 0.999 | 0.74 | 1.85 |
| *SSTR3* | 1.66 | 0.036 | 0.999 | 0.53 | 0.87 |
| *NEK8* | 1.27 | 0.036 | 0.999 | 0.6 | 0.77 |
| *FLJ40142* | 1.2 | 0.037 | 0.999 | 0.69 | 0.83 |
| *SNF1LK* | 1.29 | 0.037 | 0.999 | 0.59 | 0.76 |
| *HDAC3* | 1.1 | 0.037 | 0.999 | 0.91 | 1 |
| *ST14* | 1.44 | 0.037 | 0.999 | 2.71 | 3.89 |
| *ARNT2* | 5.76 | 0.037 | 0.999 | 1.31 | 7.56 |
| *LOC388199* | 0.49 | 0.037 | 0.999 | 1 | 0.49 |
| *CTPS* | 0.68 | 0.037 | 0.999 | 1.78 | 1.22 |
| *MDK* | 4.05 | 0.037 | 0.999 | 0.32 | 1.3 |
| *BBS12* | 1.27 | 0.037 | 0.999 | 0.79 | 1.01 |
| *DSCR3* | 1.1 | 0.038 | 0.999 | 1.02 | 1.12 |
| *ISG20* | 1.92 | 0.038 | 0.999 | 0.53 | 1.03 |
| *PLTP* | 2.54 | 0.038 | 0.999 | 1.75 | 4.44 |
| *MPPED2* | 2.23 | 0.038 | 0.999 | 0.42 | 0.94 |
| *PIB5PA* | 0.52 | 0.038 | 0.999 | 1.39 | 0.72 |
| *TCL1A* | 1.32 | 0.038 | 0.999 | 0.49 | 0.65 |
| *FAM100B* | 1.14 | 0.038 | 0.999 | 0.78 | 0.89 |
| *DNAJC11* | 0.75 | 0.038 | 0.999 | 1.53 | 1.15 |
| *NOB1* | 1.09 | 0.038 | 0.999 | 0.82 | 0.89 |
| *GRIN2D* | 1.82 | 0.038 | 0.999 | 0.99 | 1.8 |
| *RET* | 1.74 | 0.038 | 0.999 | 0.83 | 1.43 |
| *FFAR2* | 2.9 | 0.038 | 0.999 | 0.96 | 2.77 |
| *MECR* | 0.73 | 0.038 | 0.999 | 1.46 | 1.07 |
| *CHODL* | 2.28 | 0.039 | 0.999 | 0.48 | 1.1 |
| *RPUSD3* | 1.12 | 0.039 | 0.999 | 0.85 | 0.95 |
| *HIST1H3H* | 1.91 | 0.039 | 0.999 | 0.47 | 0.89 |
| *SRGAP3* | 1.44 | 0.039 | 0.999 | 0.41 | 0.59 |
| *THRB* | 0.62 | 0.039 | 0.999 | 2.71 | 1.68 |
| *ZNF471* | 0.69 | 0.039 | 0.999 | 0.93 | 0.64 |
| *HS3ST1* | 1.74 | 0.039 | 0.999 | 1.43 | 2.49 |
| *XPR1* | 0.77 | 0.039 | 0.999 | 1.4 | 1.09 |
| *HPSE* | 1.62 | 0.04 | 0.999 | 0.53 | 0.87 |
| *RGS10* | 1.14 | 0.04 | 0.999 | 0.82 | 0.93 |
| *INTS9* | 1.11 | 0.04 | 0.999 | 0.94 | 1.04 |
| *KCNT1* | 0.58 | 0.04 | 0.999 | 0.7 | 0.4 |
| *CAMKK2* | 1.17 | 0.04 | 0.999 | 0.74 | 0.86 |
| *IGSF9* | 0.55 | 0.04 | 0.999 | 0.57 | 0.31 |
| *RAB3IL1* | 2.08 | 0.04 | 0.999 | 1.22 | 2.52 |
| *MACF1* | 0.71 | 0.04 | 0.999 | 0.89 | 0.63 |
| *MARCH10* | 1.94 | 0.04 | 0.999 | 0.42 | 0.81 |
| *ILDR1* | 1.7 | 0.04 | 0.999 | 0.59 | 1.01 |
| *CD1E* | 0.16 | 0.04 | 0.999 | 52.39 | 8.12 |
| *GSTM4* | 0.74 | 0.041 | 0.999 | 1.48 | 1.09 |
| *CCR10* | 1.45 | 0.041 | 0.999 | 0.68 | 0.99 |
| *COL6A6* | 0.51 | 0.041 | 0.999 | 0.87 | 0.45 |
| *PARP6* | 1.11 | 0.041 | 0.999 | 0.85 | 0.95 |
| *TDRD3* | 1.36 | 0.041 | 0.999 | 0.86 | 1.17 |
| *CMTM4* | 0.79 | 0.041 | 0.999 | 1.71 | 1.35 |
| *TSEN34* | 1.19 | 0.041 | 0.999 | 0.7 | 0.83 |
| *TAS2R44* | 0.51 | 0.041 | 0.999 | 0.57 | 0.29 |
| *MYLPF* | 0.57 | 0.041 | 0.999 | 1.09 | 0.62 |
| *C2orf27* | 1.78 | 0.041 | 0.999 | 0.87 | 1.55 |
| *BTN2A3* | 1.32 | 0.041 | 0.999 | 0.7 | 0.92 |
| *FCRLB* | 0.66 | 0.041 | 0.999 | 2.89 | 1.91 |
| *SEPT4* | 1.63 | 0.041 | 0.999 | 0.54 | 0.89 |
| *CTF1* | 1.62 | 0.041 | 0.999 | 0.58 | 0.94 |
| *GNAI1* | 1.64 | 0.042 | 0.999 | 0.35 | 0.57 |
| *OAZ2* | 1.18 | 0.042 | 0.999 | 1.01 | 1.19 |
| *DHDH* | 0.59 | 0.042 | 0.999 | 0.69 | 0.4 |
| *GSTP1* | 1.18 | 0.042 | 0.999 | 0.81 | 0.96 |
| *KIAA0652* | 1.1 | 0.042 | 0.999 | 0.8 | 0.88 |
| *GGT5* | 3.19 | 0.042 | 0.999 | 0.82 | 2.6 |
| *GNB3* | 1.68 | 0.042 | 0.999 | 0.44 | 0.75 |
| *TNC* | 0.26 | 0.042 | 0.999 | 23.99 | 6.34 |
| *GPR89A* | 0.67 | 0.042 | 0.999 | 1.18 | 0.79 |
| *DIP2C* | 1.3 | 0.042 | 0.999 | 0.55 | 0.72 |
| *LOC647174* | 0.47 | 0.042 | 0.999 | 0.8 | 0.38 |
| *HIVEP3* | 0.6 | 0.042 | 0.999 | 3.66 | 2.19 |
| *SLC37A2* | 1.21 | 0.043 | 0.999 | 1.01 | 1.23 |
| *DAB1* | 0.48 | 0.043 | 0.999 | 0.85 | 0.41 |
| *ZFP106* | 0.87 | 0.043 | 0.999 | 1.32 | 1.15 |
| *LMCD1* | 0.62 | 0.043 | 0.999 | 1.01 | 0.62 |
| *RBM9* | 0.69 | 0.043 | 0.999 | 1.41 | 0.97 |
| *SMPD3* | 1.29 | 0.043 | 0.999 | 0.63 | 0.81 |
| *LIMA1* | 0.76 | 0.043 | 0.999 | 1.92 | 1.46 |
| *PPFIBP1* | 0.66 | 0.043 | 0.999 | 3.02 | 2 |
| *LACTB2* | 1.14 | 0.043 | 0.999 | 1.32 | 1.52 |
| *CLIC2* | 1.36 | 0.043 | 0.999 | 0.69 | 0.94 |
| *B4GALT6* | 0.64 | 0.044 | 0.999 | 1.5 | 0.97 |
| *RAB9A* | 1.2 | 0.044 | 0.999 | 0.69 | 0.84 |
| *SETD7* | 0.83 | 0.044 | 0.999 | 1.67 | 1.38 |
| *CCDC21* | 0.76 | 0.044 | 0.999 | 1.17 | 0.89 |
| *C1S* | 2.6 | 0.044 | 0.999 | 1.12 | 2.91 |
| *PDE6D* | 1.12 | 0.044 | 0.999 | 0.94 | 1.06 |
| *DNAJC6* | 0.41 | 0.044 | 0.999 | 5.76 | 2.36 |
| *FBXL12* | 1.12 | 0.044 | 0.999 | 0.84 | 0.94 |
| *C6orf186* | 0.53 | 0.044 | 0.999 | 0.52 | 0.28 |
| *GIMAP6* | 1.15 | 0.044 | 0.999 | 0.69 | 0.79 |
| *TLE1* | 0.83 | 0.044 | 0.999 | 0.83 | 0.69 |
| *SLC5A2* | 1.65 | 0.044 | 0.999 | 0.49 | 0.8 |
| *CDCA4* | 1.1 | 0.044 | 0.999 | 0.81 | 0.89 |
| *FEZ1* | 1.42 | 0.044 | 0.999 | 0.51 | 0.73 |
| *EGR1* | 0.59 | 0.044 | 0.999 | 2.85 | 1.68 |
| *SQSTM1* | 1.12 | 0.045 | 0.999 | 0.9 | 1.02 |
| *GPRC5B* | 3.37 | 0.045 | 0.999 | 0.1 | 0.33 |
| *RRAS* | 1.38 | 0.045 | 0.999 | 0.92 | 1.27 |
| *FLT1* | 0.25 | 0.045 | 0.999 | 22.47 | 5.71 |
| *DDEFL1* | 0.64 | 0.045 | 0.999 | 0.69 | 0.44 |
| *GTPBP1* | 1.3 | 0.045 | 0.999 | 0.75 | 0.97 |
| *GPR126* | 0.59 | 0.045 | 0.999 | 1.85 | 1.09 |
| *ANKRD35* | 0.48 | 0.045 | 0.999 | 2.19 | 1.06 |
| *RGPD1* | 0.58 | 0.045 | 0.999 | 0.99 | 0.58 |
| *MYOZ1* | 0.24 | 0.045 | 0.999 | 15.69 | 3.78 |
| *UBA7* | 1.23 | 0.045 | 0.999 | 0.78 | 0.97 |
| *FBXL14* | 1.14 | 0.045 | 0.999 | 0.77 | 0.88 |
| *LANCL3* | 0.39 | 0.045 | 0.999 | 1.25 | 0.49 |
| *DENND1B* | 0.71 | 0.045 | 0.999 | 1.62 | 1.14 |
| *IFI6* | 3.97 | 0.046 | 0.999 | 0.64 | 2.56 |
| *DBF4B* | 1.3 | 0.046 | 0.999 | 0.72 | 0.94 |
| *IL4R* | 0.89 | 0.046 | 0.999 | 1.07 | 0.95 |
| *SOD1* | 1.12 | 0.046 | 0.999 | 0.86 | 0.96 |
| *ACSBG2* | 0.57 | 0.046 | 0.999 | 0.79 | 0.45 |
| *SLC37A1* | 1.26 | 0.046 | 0.999 | 0.92 | 1.16 |
| *UNC93B1* | 1.66 | 0.046 | 0.999 | 0.92 | 1.52 |
| *PDCD6* | 1.09 | 0.046 | 0.999 | 0.91 | 1 |
| *CREBL2* | 1.18 | 0.046 | 0.999 | 0.73 | 0.86 |
| *EXOC2* | 1.11 | 0.046 | 0.999 | 0.81 | 0.91 |
| *NAAA* | 1.19 | 0.046 | 0.999 | 0.88 | 1.05 |
| *TNFRSF6B* | 1.27 | 0.046 | 0.999 | 0.84 | 1.06 |
| *NAGLU* | 1.17 | 0.046 | 0.999 | 0.95 | 1.11 |
| *ADA* | 1.4 | 0.046 | 0.999 | 0.45 | 0.64 |
| *ARID1A* | 0.76 | 0.047 | 0.999 | 0.94 | 0.72 |
| *PRKAR2A* | 0.91 | 0.047 | 0.999 | 1.25 | 1.13 |
| *ZNF358* | 1.27 | 0.047 | 0.999 | 0.66 | 0.84 |
| *SFXN2* | 0.88 | 0.047 | 0.999 | 0.95 | 0.83 |
| *GGA3* | 1.13 | 0.047 | 0.999 | 0.8 | 0.91 |
| *HHIPL1* | 0.54 | 0.047 | 0.999 | 0.56 | 0.3 |
| *CXorf39* | 0.92 | 0.047 | 0.999 | 1.03 | 0.95 |
| *PEBP1* | 1.15 | 0.047 | 0.999 | 0.74 | 0.85 |
| *HIST1H2BH* | 0.53 | 0.047 | 0.999 | 0.78 | 0.41 |
| *BCL2L13* | 1.09 | 0.047 | 0.999 | 1.04 | 1.13 |
| *CDK2* | 1.11 | 0.047 | 0.999 | 0.87 | 0.97 |
| *C11orf67* | 1.33 | 0.047 | 0.999 | 0.67 | 0.89 |
| *NUDT5* | 1.09 | 0.047 | 0.999 | 1.02 | 1.11 |
| *RENBP* | 1.33 | 0.048 | 0.999 | 0.91 | 1.21 |
| *DYSF* | 1.7 | 0.048 | 0.999 | 0.57 | 0.96 |
| *ADAD2* | 0.38 | 0.048 | 0.999 | 0.58 | 0.22 |
| *FH* | 0.72 | 0.048 | 0.999 | 1.89 | 1.35 |
| *PHF11* | 1.27 | 0.048 | 0.999 | 0.93 | 1.18 |
| *DCLK2* | 0.39 | 0.048 | 0.999 | 8.52 | 3.36 |
| *C17orf39* | 1.16 | 0.048 | 0.999 | 0.72 | 0.84 |
| *UBE4B* | 0.78 | 0.048 | 0.999 | 1.1 | 0.85 |
| *WSCD1* | 0.61 | 0.048 | 0.999 | 2.03 | 1.25 |
| *ASMT* | 0.58 | 0.048 | 0.999 | 0.8 | 0.46 |
| *LYPD5* | 1.92 | 0.048 | 0.999 | 0.88 | 1.69 |
| *KIAA1257* | 1.81 | 0.048 | 0.999 | 0.6 | 1.09 |
| *EPC2* | 1.16 | 0.048 | 0.999 | 0.74 | 0.86 |
| *PLEKHA6* | 0.62 | 0.048 | 0.999 | 0.71 | 0.44 |
| *GLDC* | 1.88 | 0.048 | 0.999 | 0.84 | 1.58 |
| *CORO2A* | 0.85 | 0.048 | 0.999 | 1.01 | 0.86 |
| *P2RY6* | 1.68 | 0.048 | 0.999 | 0.98 | 1.65 |
| *ME3* | 1.17 | 0.049 | 0.999 | 1.04 | 1.22 |
| *GFPT1* | 0.88 | 0.049 | 0.999 | 1.17 | 1.03 |
| *HEXDC* | 1.19 | 0.049 | 0.999 | 0.72 | 0.86 |
| *GLB1L3* | 0.58 | 0.049 | 0.999 | 0.74 | 0.43 |
| *EFR3B* | 0.65 | 0.049 | 0.999 | 1.68 | 1.1 |
| *B3GALNT2* | 0.76 | 0.049 | 0.999 | 1.37 | 1.04 |
| *INTS3* | 0.8 | 0.049 | 0.999 | 1.1 | 0.88 |
| *CXCL6* | 2.52 | 0.049 | 0.999 | 7.73 | 19.47 |
| *SLC9A1* | 0.77 | 0.049 | 0.999 | 1.33 | 1.02 |
| *OR52B6* | 2.74 | 0.049 | 0.999 | 0.51 | 1.39 |
| *RRAGD* | 1.24 | 0.049 | 0.999 | 1.06 | 1.31 |
| *SLC12A7* | 1.25 | 0.049 | 0.999 | 0.79 | 0.98 |
| *WARS2* | 0.73 | 0.049 | 0.999 | 1.29 | 0.94 |
| *PCDH1* | 0.66 | 0.05 | 0.999 | 0.52 | 0.34 |
| *DHX9* | 0.75 | 0.05 | 0.999 | 1.11 | 0.83 |

^a^Gene symbol is provided for gene identification

^b^Fold change for the interaction (HS/HU)**/**(LS/LU)

^c^P-value and false discovery rate for the interaction

^d^Fold change in High responders, stimulated vs. unstimulated samples (HS/HU)

^e^Fold change in Low responders, stimulated vs. unstimulated samples (LS/LU)
